# Supplementary material for: CDKAL1 dysfunction impairs lysine codon translation in podocytes and accelerates chronic kidney disease
Source: EMBO J. 2026 Mar 28;45(9):3206–29. doi: 10.1038/s44318-026-00759-3 (PMC13144697; doi:10.1038/s44318-026-00759-3)
Supplement: Supplementary file 1 — Appendix [file 44318_2026_759_MOESM1_ESM.pdf]

Appendix

CDKAL1 dysfunction impairs lysine codon translation in podocytes and accelerates chronic kidney disease

Hiroko Nagata, Yu Nagayoshi, Takeshi Chujo, Hitomi Kaneko, Kayo Nishiguchi, Yutaka Kakizoe, Hiroko Ijima, Korin Sakakida, Takeshi Masuda, Sumio Ohtsuki, Fan-Yan Wei, Yukie Takahashi, Takaichi Fukuda, Hideaki Jinnouchi, Yuki Adachi, Ryosuke Yamamura, Koki Matsushita, Masataka Adachi, Hideki Yokoi, Kimitoshi Nakamura, Hitoshi Nakazato, and Kazuhito Tomizawa

Appendix Fig. S1: 2

Appendix Fig. S2: 2

Appendix Fig. S3: 3

Appendix Fig. S4: 4

Appendix Fig. S5: 5

Appendix Fig. S6: 6

Appendix Fig. S7: 7

Appendix Fig. S8: 7

Appendix Fig. S9: 8-10

Appendix Fig.S10: 10

Appendix Fig.S11: 10

Appendix Table S1: 11

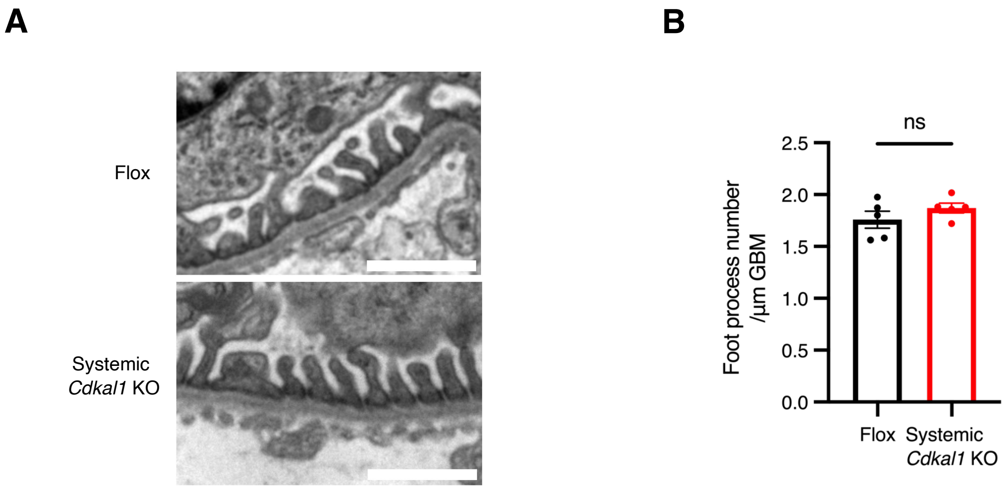

Appendix Figure S1. No foot process effacement in eight-week-old systemic *Cdkal1* KO mice. (**A**) Scanning electron microscopy images of glomeruli of 8-week-old systemic *Cdkal1* KO mice. Scale bars, 1 µm. (**B**) Foot process number/µm GBM of systemic *Cdkal1* KO mice. $n = 5$ each. Data are presented as mean ± SEM. Statistical significance was assessed using the Mann–Whitney *U* test.

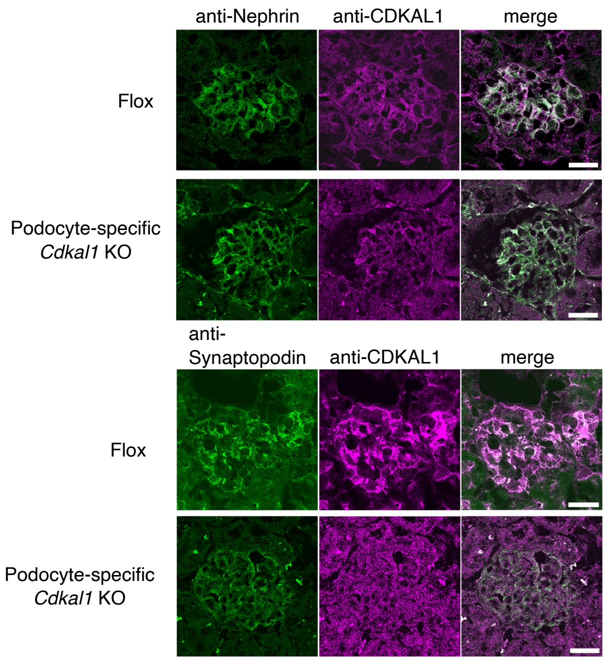

Appendix Figure S2. Reduction of podocyte-specific proteins in immunofluorescence staining of podocyte-specific *Cdkal1* KO mice. (**A**) Double immunofluorescence staining for nephrin and synaptopodin (green) with CDKAL1 (magenta) in the glomeruli of 20-week-old male mice. Nephrin and synaptopodin are the podocyte markers. Scale bars, 20 µm.

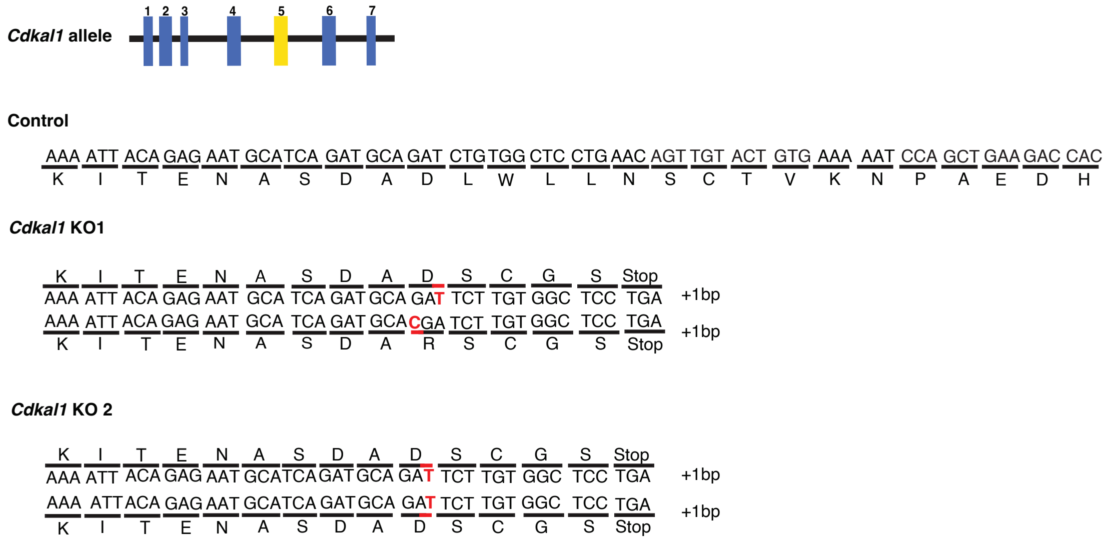

Appendix Figure S3. *Cdkal1* alleles in *Cdkal1* KO E11 podocyte cell lines. The encoded amino acids are indicated below or above the corresponding codons. Red letters in the mutated alleles indicate base insertions.

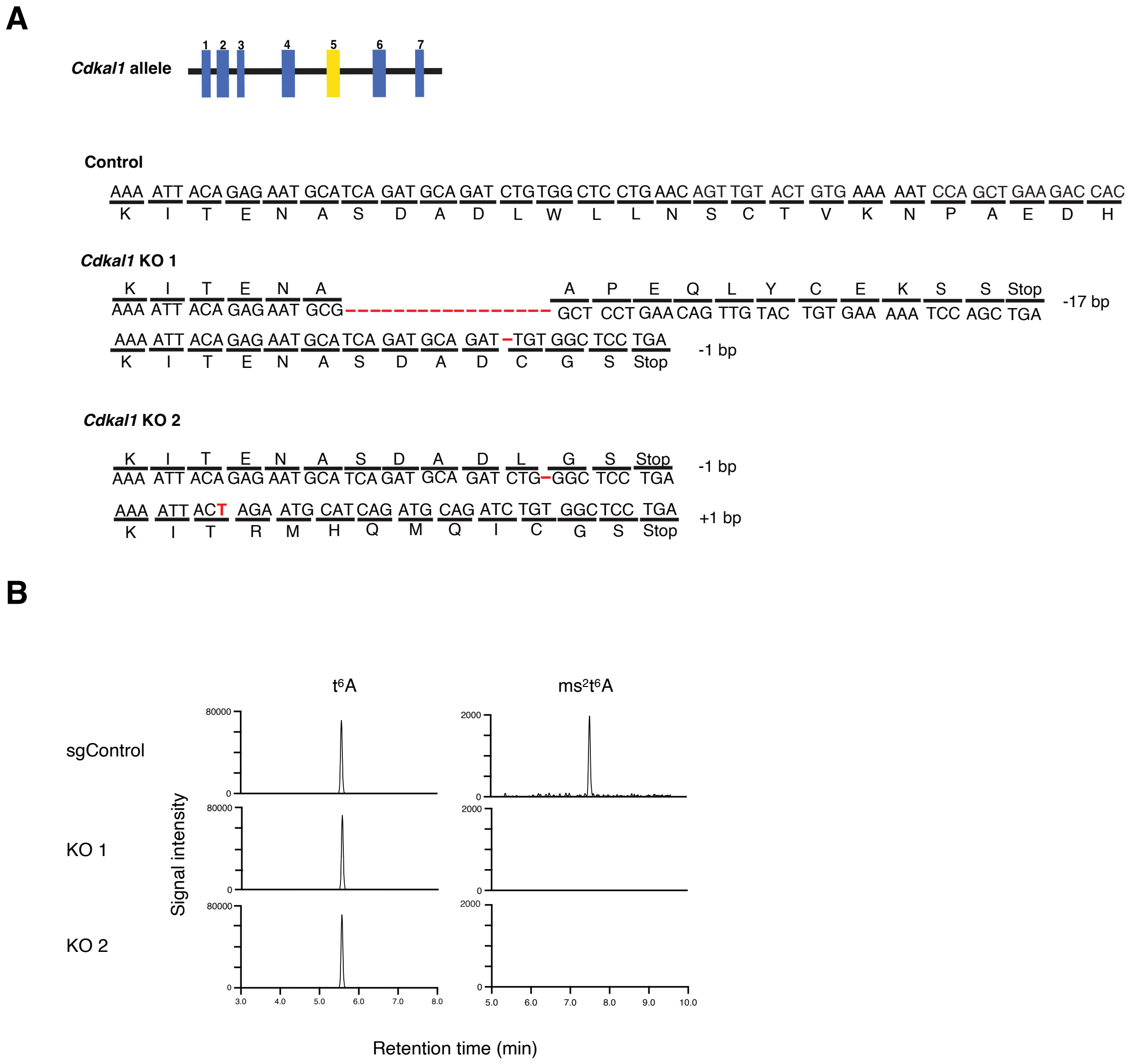

Appendix Figure S4. Generation of *Cdkal1* KO SVI podocyte cell lines. (**A**) *Cdkal1* alleles in *Cdkal1* KO SVI. The encoded amino acids are indicated below or above the corresponding codons. Red letter in the mutated allele indicates base insertion, and red lines indicate base deletions. (**B**) Lack of ms^{2}t^{6}A modification within total RNA of *Cdkal1* KO SVI podocyte cells, as confirmed by LC–MS.

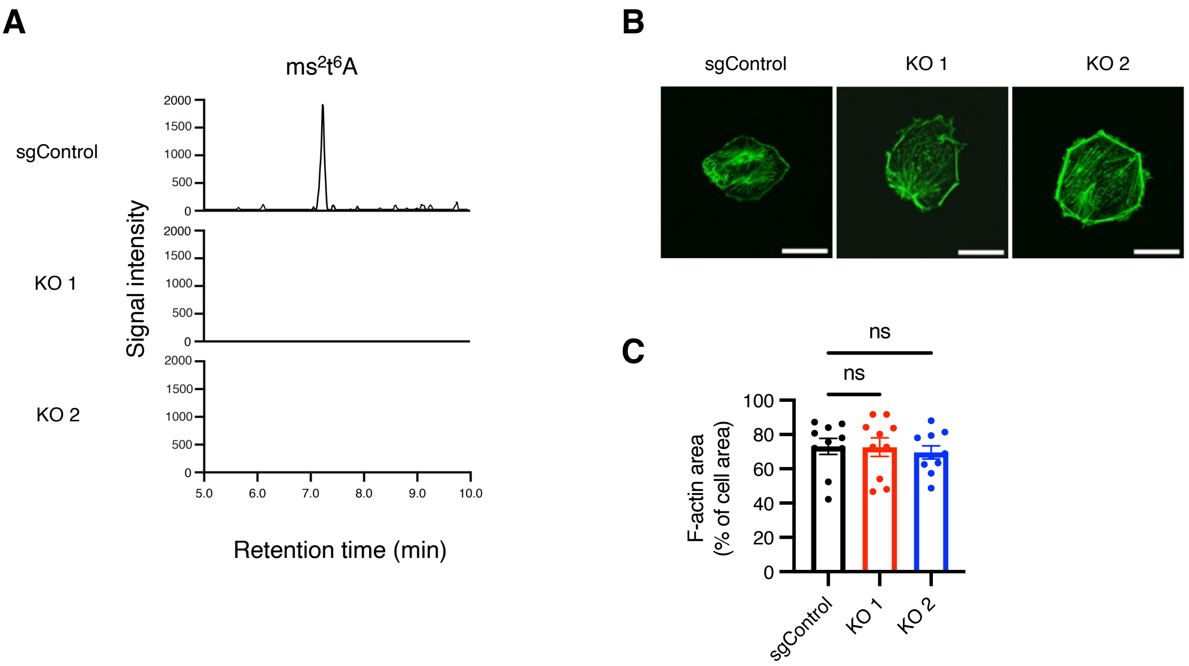

Appendix Figure S5. *CDKAL1* KO has no effect on F-actin organization in HuH-7 hepatocellular carcinoma cells. (**A**) Loss of ms^{2}t^{6}A modification within total RNA of *CDKAL1* KO HuH-7 cells, as confirmed by LC–MS. (**B**) Representative images of F-actin stained with phalloidin in C*DKAL1* KO HuH-7 cells. Scale bar, 20 µm. (**C**) Quantification of the cellular area occupied by F-actin. $n = 10$; data are presented as the mean ± SEM; n.s., not significant by one-way ANOVA, followed by Dunnett's correction.

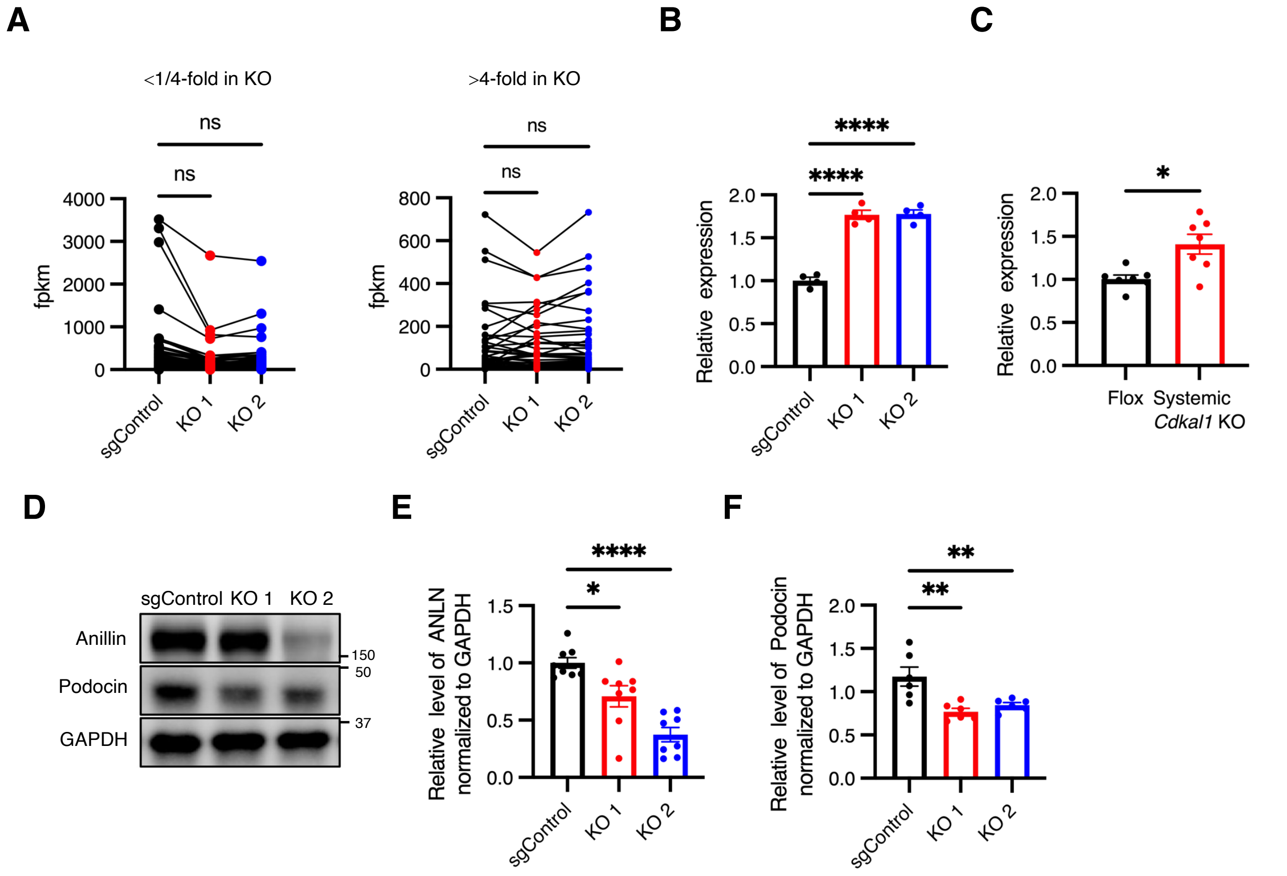

Appendix Figure S6. Post-transcriptional dysregulation of podocyte structural proteins in *Cdkal1* KO podocytes and glomeruli. (**A**) mRNA quantification using mRNA-seq results for the proteins shown in Fig. 4A. n.s., not significant by the Kruskal–Wallis test and Dunn's multiple comparison test. (**B**) RT-qPCR analysis of *Cd2ap* mRNA in *Cdkal1* KO SVI. $n = 4$. Data are presented as the mean ± SEM. ****$P < 0.0001$ by one-way ANOVA followed by Dunnett correction. (**C**) RT-qPCR analysis of *Cd2ap* mRNA in the mouse sieved glomeruli of systemic *Cdkal1* KO mice. $n = 6$. Data are presented as the mean ± SEM. *$P = 0.0180$ by Student's *t*-test. (**D**) Representative western blot images of anillin and podocin (encoded by *NPHS2*), lysine-rich protein in *Cdkal1* KO, and control SVI podocytes. (**E, F**) Quantification of anillin (**E**) and podocin (**F**) levels normalized to GAPDH in *Cdkal1* KO and control SVI podocytes. $n = 6–8$ each. Data are presented as the mean ± SEM. ****$P < 0.0001$, **$P < 0.01$, and *$P < 0.05$ by one-way ANOVA, followed by Dunnett's correction.

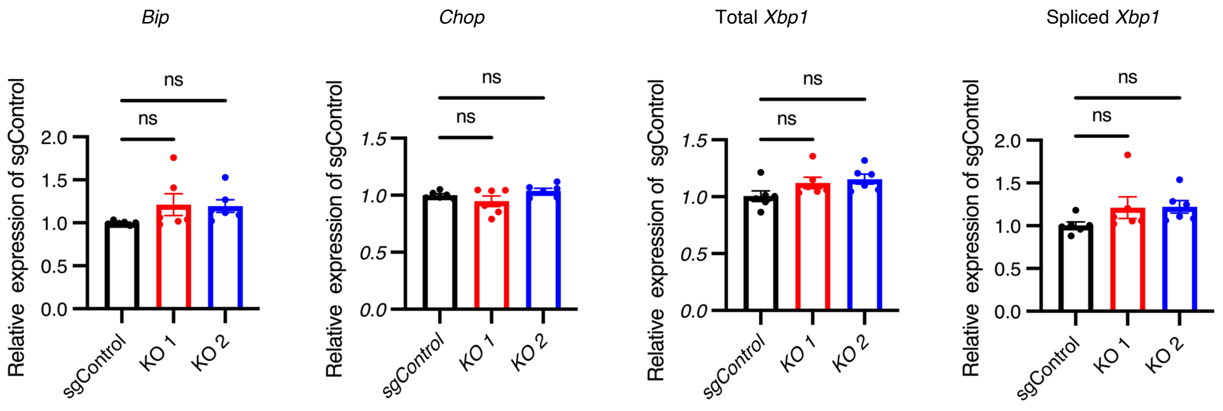

Appendix Figure S7. ER stress marker mRNA levels in *Cdkal1* KO E11. RT-qPCR of ER stress-related genes in *Cdkal1* KO and sgControl mice. n.s., not significant by the Kruskal–Wallis test and Dunn's multiple comparison test.

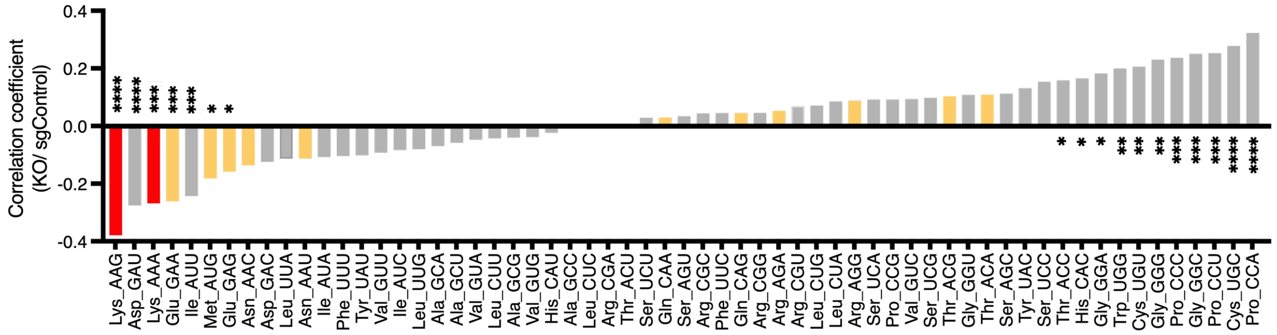

Appendix Figure S8. Pearson correlation between codon-usage frequency and protein level changes among upregulated or downregulated proteins in *Cdkal1* KO cells. Lysine codons are indicated in red and their near-cognate codons are indicated in yellow. ****$P < 0.0001$ (Lys_AAG), ****$P < 0.0001$ (Asp_GAU), ***$P = 0.0001$ (Lys_AAA), ***$P = 0.0002$ (Glu_GAA), ***$P = 0.0066$ (Ile_AUU), *$P = 0.0106$ (Met_AUG), *$P = 0.0263$ (Glu_GAG), *$P = 0.0265$ (Thr_ACC), *$P = 0.0203$ (His_CAC), *$P = 0.0103$ (Gly_GGA), **$P = 0.0049$ (Trp_UGG), **$P = 0.0037$ (Cys_UGU), **$P = 0.0012$ (Gly_GGG), ***$P = 0.0008$ (Pro_CCC), ***$P = 0.0004$ (Gly_GGC), ***$P = 0.0003$ (Pro_CCU), ****$P < 0.0001$ (Cys_UGC), ****$P < 0.0001$ (Pro_CCA) by Pearson correlation test. No significant changes were observed in unindicated codons.

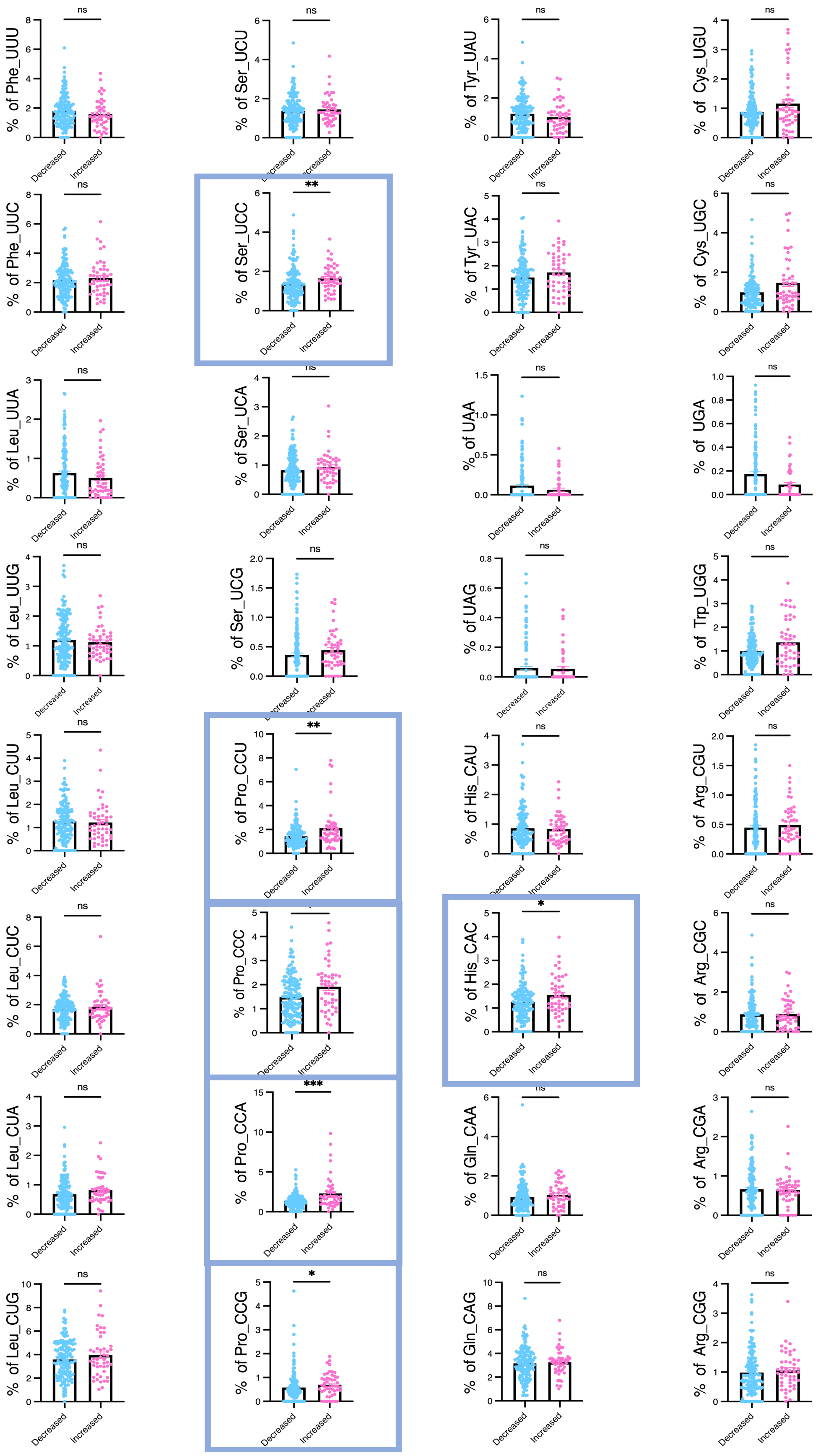

(This figure continues on the following page.)

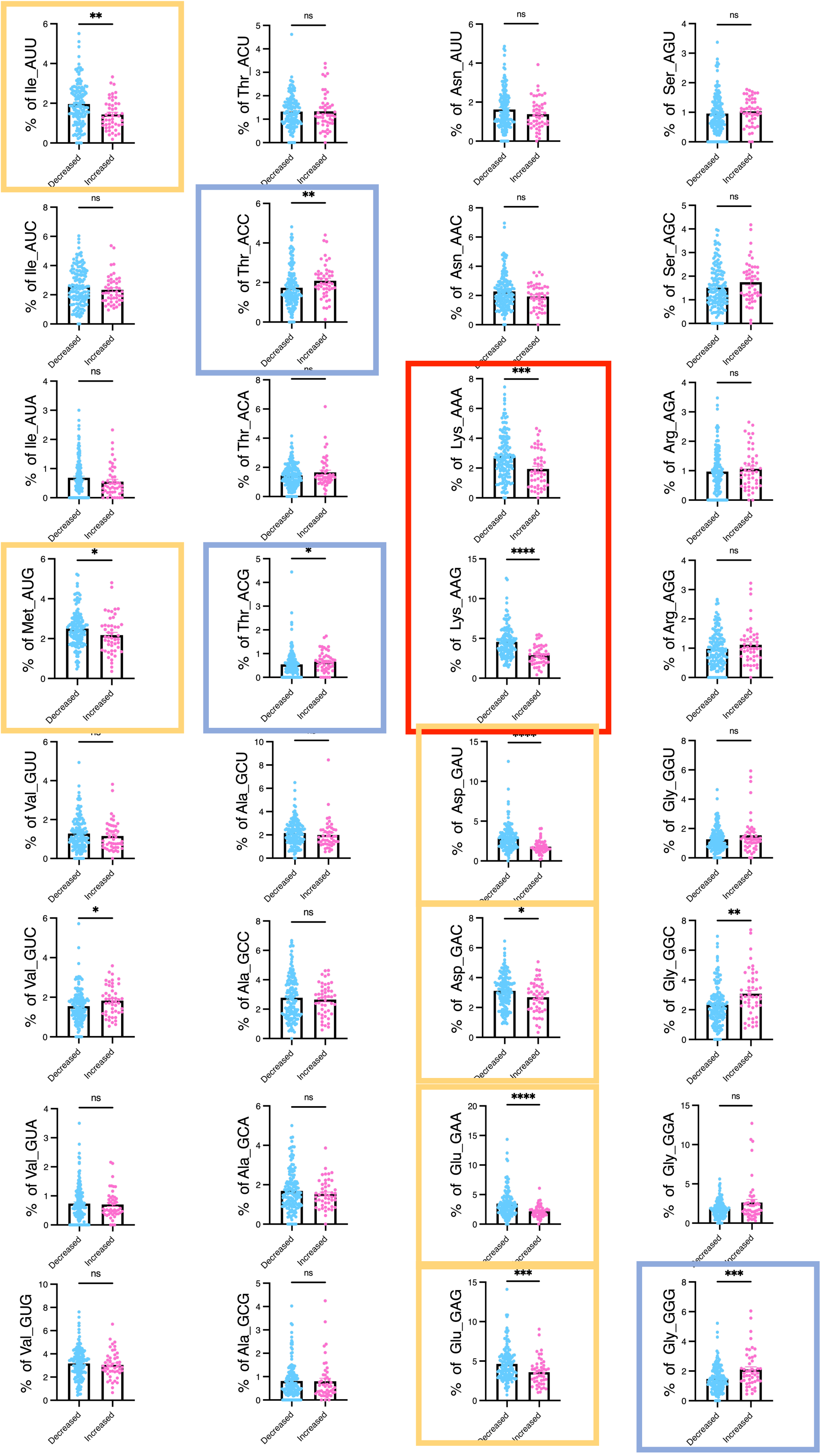

**
% of Ile_AUU
ns
% of Ile_AUC
ns
% of Ile_AUA
*
% of Met_AUG
ns
% of Val_GUU
*
% of Val_GUC
ns
% of Val_GUA
ns
% of Val_GUG
ns
% of Thr_ACU
**
% of Thr_ACC
ns
% of Thr_ACA
*
% of Thr_ACG
% of Ala_GCU
ns
% of Ala_GCC
ns
% of Ala_GCA
ns
% of Ala_GCG
ns
% of Asn_AUU
ns
% of Asn_AAC
***
% of Lys_AAA
****
% of Lys_AAG
% of Asp_GAU
*
% of Asp_GAC
****
% of Glu_GAA
***
% of Glu_GAG
ns
% of Ser_AGU
ns
% of Ser_AGC
ns
% of Arg_AGA
ns
% of Arg_AGG
ns
% of Gly_GGU
**
% of Gly_GGC
ns
% of Gly_GGA
***
% of Gly_GGG
Decreased
Increased

Appendix Figure S9. Relative codon composition of proteins downregulated and upregulated in *Cdkal1* KO cells.

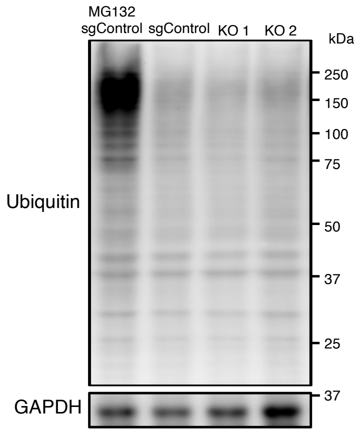

Appendix Figure S10. Ubiquitination in *Cdkal1* KO podocytes. Western blotting analysis of ubiquitinated proteins in *Cdkal1* KO and control podocytes. SVI sgControl cells were treated with MG132 as a positive marker. GAPDH was used as a loading control.

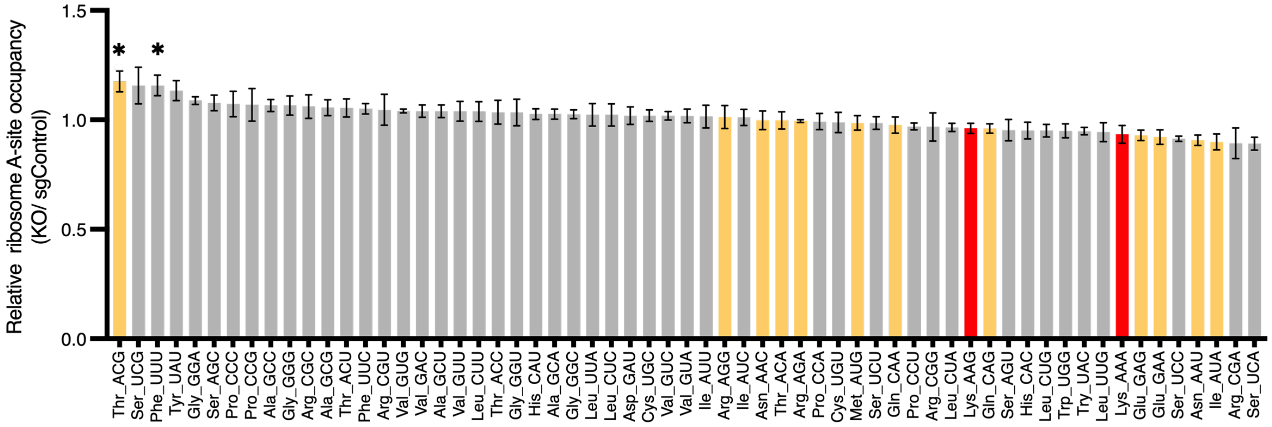

Appendix Figure S11. Ribosome A-site occupancy across mRNA codons in WT and *Cdkal1* KO SVI podocytes (mean ± SEM, *n* = 4 per group). Lysine codons are indicated in red and their near-cognate codons are indicated in yellow. *P = 0.02249 (Thr_ACG), *P = 0.0297 (Phe_TTT) by Welch's *t*-test. No significant changes were observed in unindicated codons.

	CDKAL1 Risk Allele (+) *N*=35	*CDKAL1* Risk Allele (-) *N*=9	*P* value
Age	54 (48, 61)	60 (57.5, 67)	**0.032**
Gender male, n (%)	31 (89)	8 (89)	0.98
Body height (cm)	167.8 (163.8, 172.4)	166 (160.7, 172.5)	0.64
Body weight (kg)	70.4 (65.2, 79.9)	70.2 (59.9, 70.5)	0.26
BMI (kg/m^2)	25.3 (23.7, 27.2)	24.3 (23.3, 25.25)	0.12
DM year (years)	9 (6, 14)	14 (10.5, 18.5)	**0.046**
HgbA1c (%)	6.8 (6.6, 7.3)	7 (6.65, 7.35)	0.42
BUN (mg/dL)	19.2 (17.6, 21.7)	20.2 (18.6, 23.85)	0.29
Creatinine (mg/dL)	0.96 (0.85, 1.07)	0.97 (0.83, 1.135)	0.84
eGFR (mL/min/1.73 m^2)	64.2 (57.2, 70.9)	58.1 (52.8, 75.4)	0.41

Appendix Table S1. Physical examination and laboratory findings in the two groups. Summary of the clinical characteristics of the patients in the *CDKAL1* SNP risk allele (+) and (–) groups. Values are median (interquartile range) or numbers and percentages (categorical data). Bold values indicate statistically significant differences. The Wilcoxon signed-rank test was used for univariate analysis. BMI, body mass index; HbA1c, hemoglobin A1c; BUN, blood urea nitrogen; eGFR, estimated glomerular filtration rate. eGFR was calculated using the conversion formula for Japanese people of the Japanese Society of Nephrology CKD Initiative (JSN-CKDI) (Matsuo *et al*, 2009). Continuous variables were summarized as medians and interquartile ranges due to non-normal distribution, and were analyzed using non-parametric methods. Categorical variables were expressed as frequencies and percentages.

Appendix Supplementary Methods

Histologic analysis

After sacrifice, the kidneys of the mice were harvested, fixed in Duboscq–Brazil fluids for 24 h, paraffin-embedded, and sections were cut at 2 μm using a cryostat (Leica, CM1510S). Sections were stained using HE, PAS, and Azan–Mallory according to standard protocols and examined under a light microscope (Olympus, BX51).

Immunofluorescence

For double immunostaining, the sections were incubated overnight at 37 °C with a guinea pig anti-nephrin polyclonal antibody (1:50, PROGEN) and a rabbit anti-CDKAL1 polyclonal antibody (1:75, Proteintech). The next day, secondary goat anti-guinea pig Alexa 488 (1:100, Abcam) and goat anti-rabbit Alexa 647 (1:200, Invitrogen) antibodies were added and the sections were incubated for 1 h at RT in the dark. For concomitant staining for synaptopodin/CDKAL1, FlexAble CoraLite Plus 488 (Proteintech, 1 μL) and FlexAble CoraLite Plus 647 (Proteintech, 3.5 μL) kits were used with rabbit anti-Synaptopodin (0.45 μg, Proteintech 21064-1-AP) and anti-CDKAL1 (1.75 μg, Proteintech) antibodies, respectively, and the sections were incubated for 2 h at RT. Images were obtained using a confocal laser scanning microscope (Olympus, FV3000).

RNA sequencing and RT–PCR

E11 or SVI cells in 10-cm dishes were briefly washed with PBS and lysed in 1 mL of TRI Reagent, and total RNA was extracted according to the manufacturer's protocol. The sieved glomeruli were pelleted by centrifugation at 300 × *g* for 1 min and lysed using TRI Reagent. RNA purity and concentration were measured using NanoDrop One (Thermo Fisher Scientific). RT-qPCR was performed using Prime Script RT Master Mix (Takara), Rotor Gene 2 (Qiagen), and TB Green Premix Ex Taq II (Takara) using standard protocols and primers listed in Appendix Table S1. RNA sequencing and data analyses were conducted by Novogene Co., Ltd. (Beijing, China). Gene expression profiles were generated based on the number of fragments per kilobase of exons per million mapped reads.

Wound healing migration assay

Cells were seeded at 100,000 cells/well in a 12-well culture plate. After 12 h of growth, mechanical scraping was performed with a 200-μL pipette tip from the top to the bottom of the well, as previously described (Cechova *et al*, 2018). Images of each scratched area were obtained immediately after scraping (0 h) and after 30 h, using a light microscope (Olympus, CKX53). A magnification of 4× was used for imaging, and the scraped area at each time point was measured using the ImageJ software (1.53v; NIH). The percentage of the area at 30 h relative to that at 0 h for cells of each genotype was calculated. Five replicates were used for each assay.

Western blotting

Protein extraction and immunoblotting were performed as described in the *Methods* section, except that the following primary antibodies were used: rabbit anti-ANLN (anillin) antibody (Sigma, 1:10,000), rabbit anti-NPHS2 (podocin) antibody (Abcam, 1:1,000) and mouse anti-ubiquitin (Cell Signal Technology, 1:1000).

Clinical study

We enrolled 45 patients who had been diagnosed with type 2 diabetes mellitus at Jinnouchi Hospital between January 26 and September 30, 2023. The SNP (rs7756992) in *CDKAL1* was analyzed using the TaqMan SNP Genotyping Assay Kit (Life Technologies). We collected the data of these patients, including blood and urine test results, from their clinical records.

Statistics

The Wilcoxon signed-rank test was used for univariate analysis and Fisher's exact test was used to compare categorical datasets in the clinical study. Analyses were performed using JMP version 9.0 (SAS Campus Drive). Data were analyzed using GraphPad Prism 10.2 software. Statistical significance was set at $P < 0.05$.

Study approval

This clinical study was conducted in accordance with the principles of the Declaration of Helsinki and the ethical guidelines for Medical and Health Research Involving Human Subjects of the Ministry of Health, Labour, and Welfare (MEXT) of Japan. Written informed consent was obtained from all patients. This study was approved by the Institutional Ethics Board of Kumamoto University (approval no. 1671 and Kumamoto University Genome no. 528) and Jinnouchi Hospital (approval no. 2019-7-2).

Reference

Cechova S, Dong F, Chan F, Kelley MJ, Ruiz P, Le TH (2018) MYH9 E1841K Mutation Augments Proteinuria and Podocyte Injury and Migration. *J Am Soc Nephrol* 29: 155-167

Matsuo S, Imai E, Horio M, Yasuda Y, Tomita K, Nitta K, Yamagata K, Tomino Y, Yokoyama H, Hishida A (2009) Revised equations for estimated GFR from serum creatinine in Japan. *Am J Kidney Dis* 53: 982-992
