# Supplementary figures and images for: CDKAL1 dysfunction impairs lysine codon translation in podocytes and accelerates chronic kidney disease (part 1 of 2)
Source: EMBO J. 2026 Mar 28;45(9):3206–29. doi: 10.1038/s44318-026-00759-3 (PMC13144697; doi:10.1038/s44318-026-00759-3)

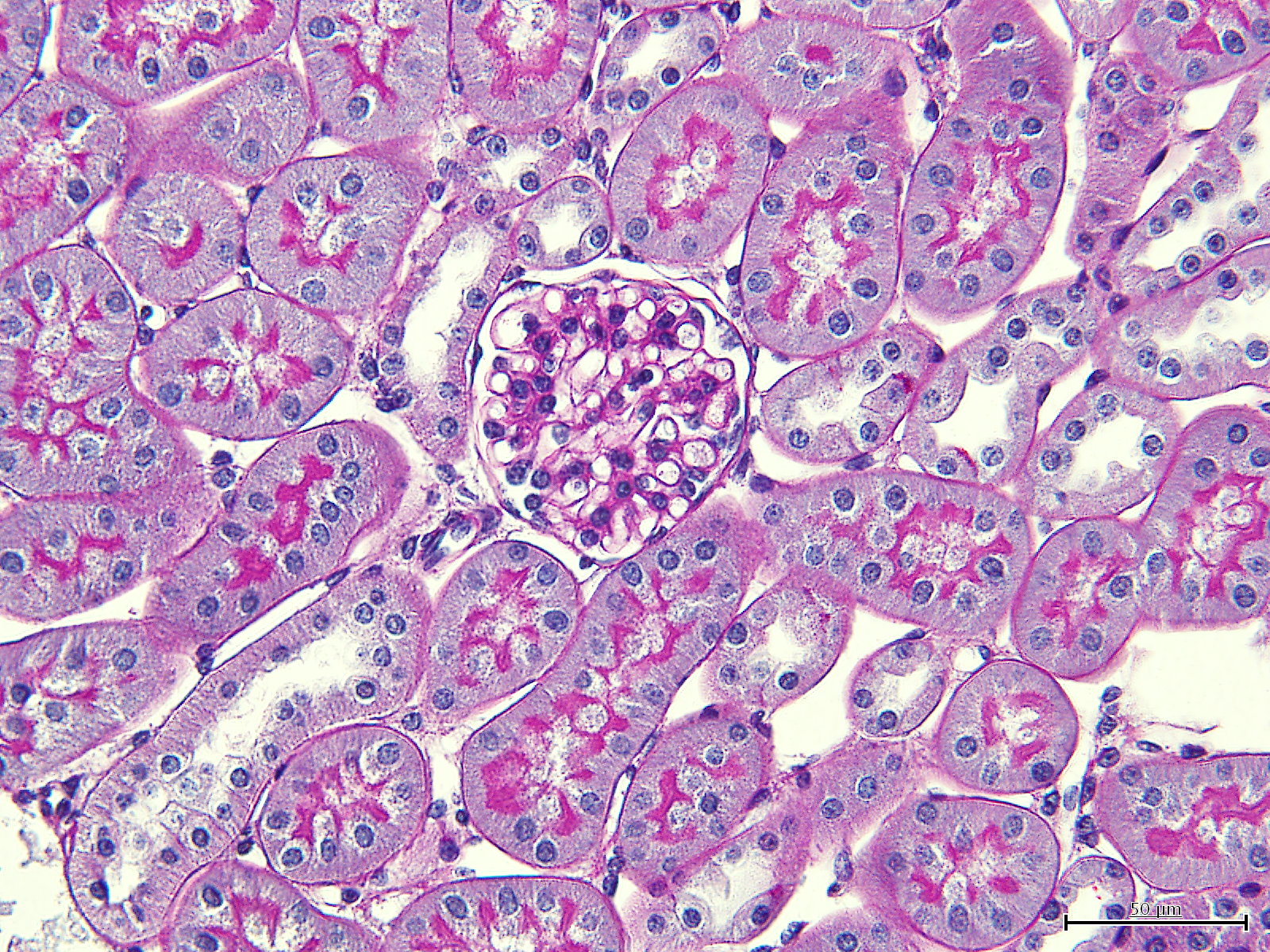

Supplement: Supplementary file 3 — Source data Fig. 1 [file 44318_2026_759_MOESM3_ESM.zip › SD Figure 1/1H/Flox_HighMagnification.TIF]

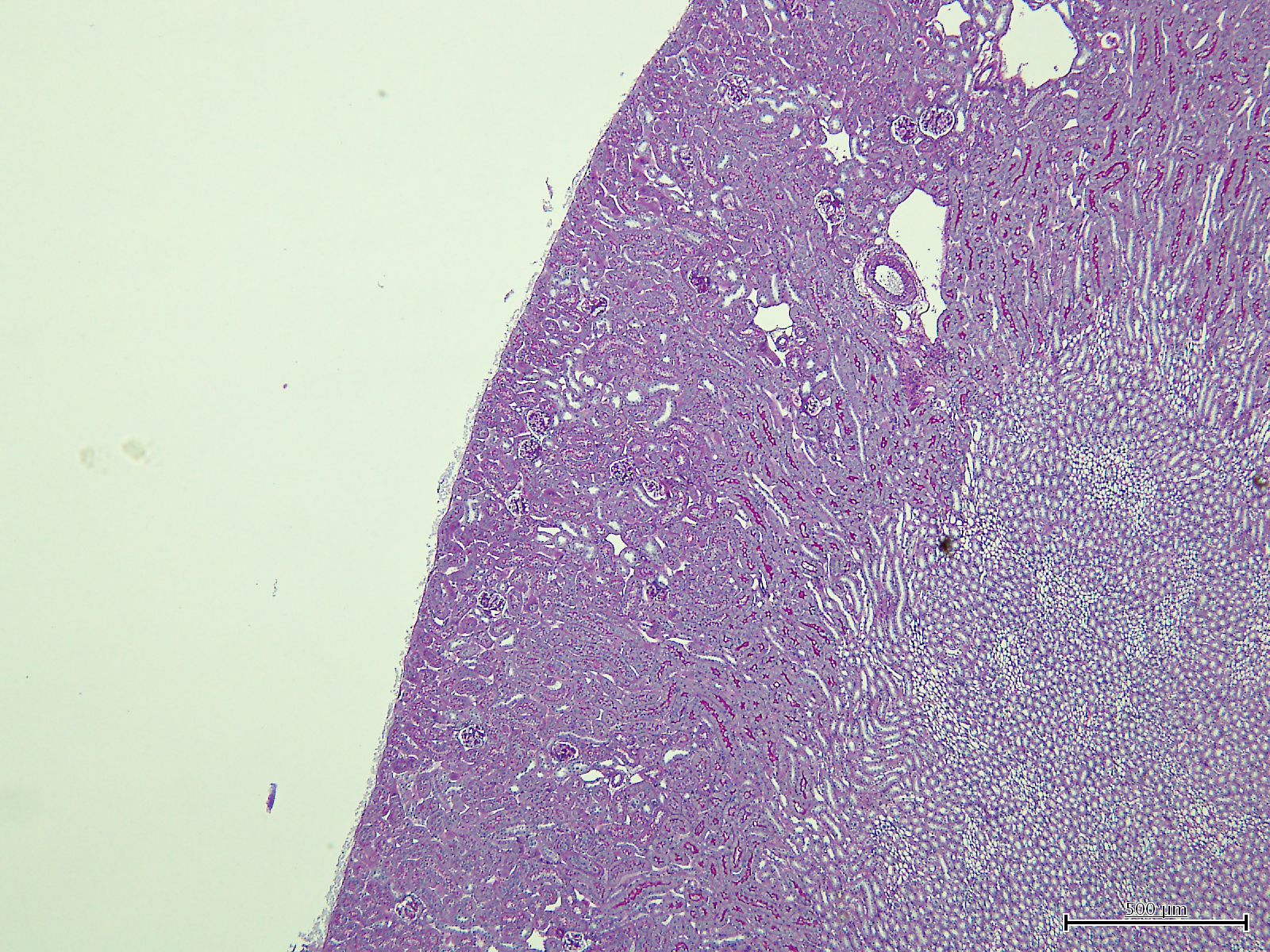

Supplement: Supplementary file 3 — Source data Fig. 1 [file 44318_2026_759_MOESM3_ESM.zip › SD Figure 1/1H/Flox_LowMagnification.TIF]

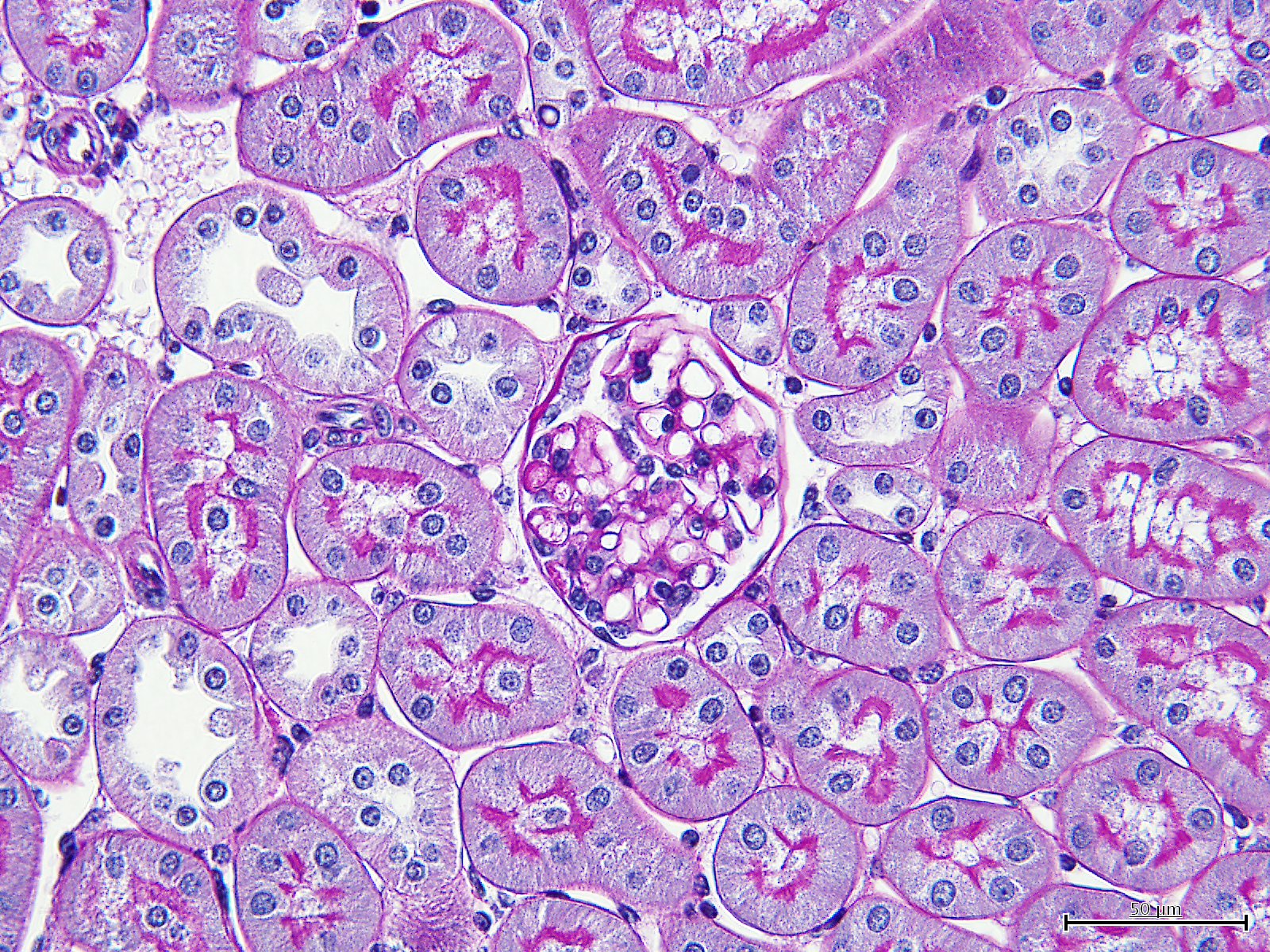

Supplement: Supplementary file 3 — Source data Fig. 1 [file 44318_2026_759_MOESM3_ESM.zip › SD Figure 1/1H/Systemic-Cdkal1 KO_HighMagnification.TIF]

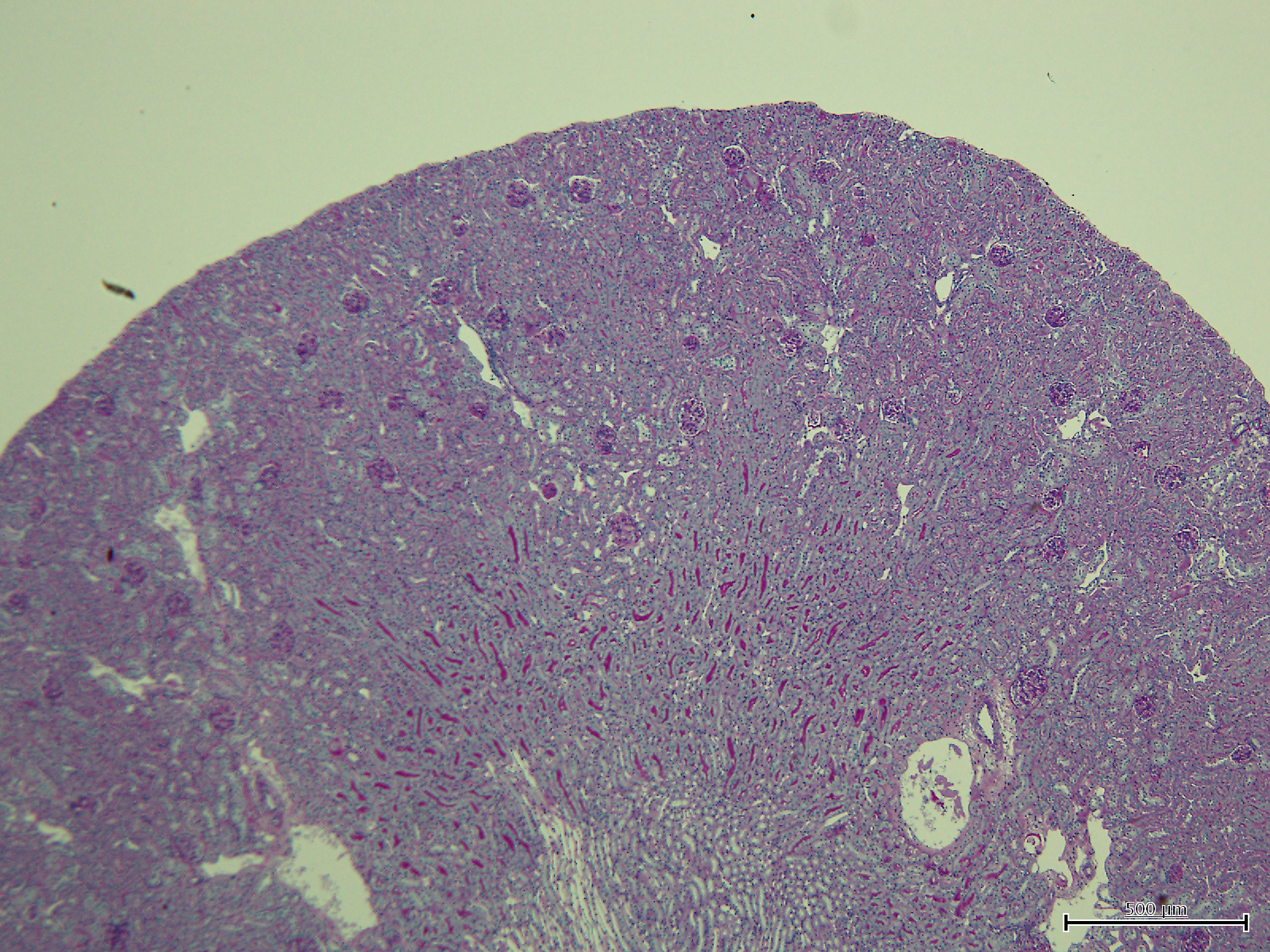

Supplement: Supplementary file 3 — Source data Fig. 1 [file 44318_2026_759_MOESM3_ESM.zip › SD Figure 1/1H/Systemic-Cdkal1 KO_LowMagnification.TIF]

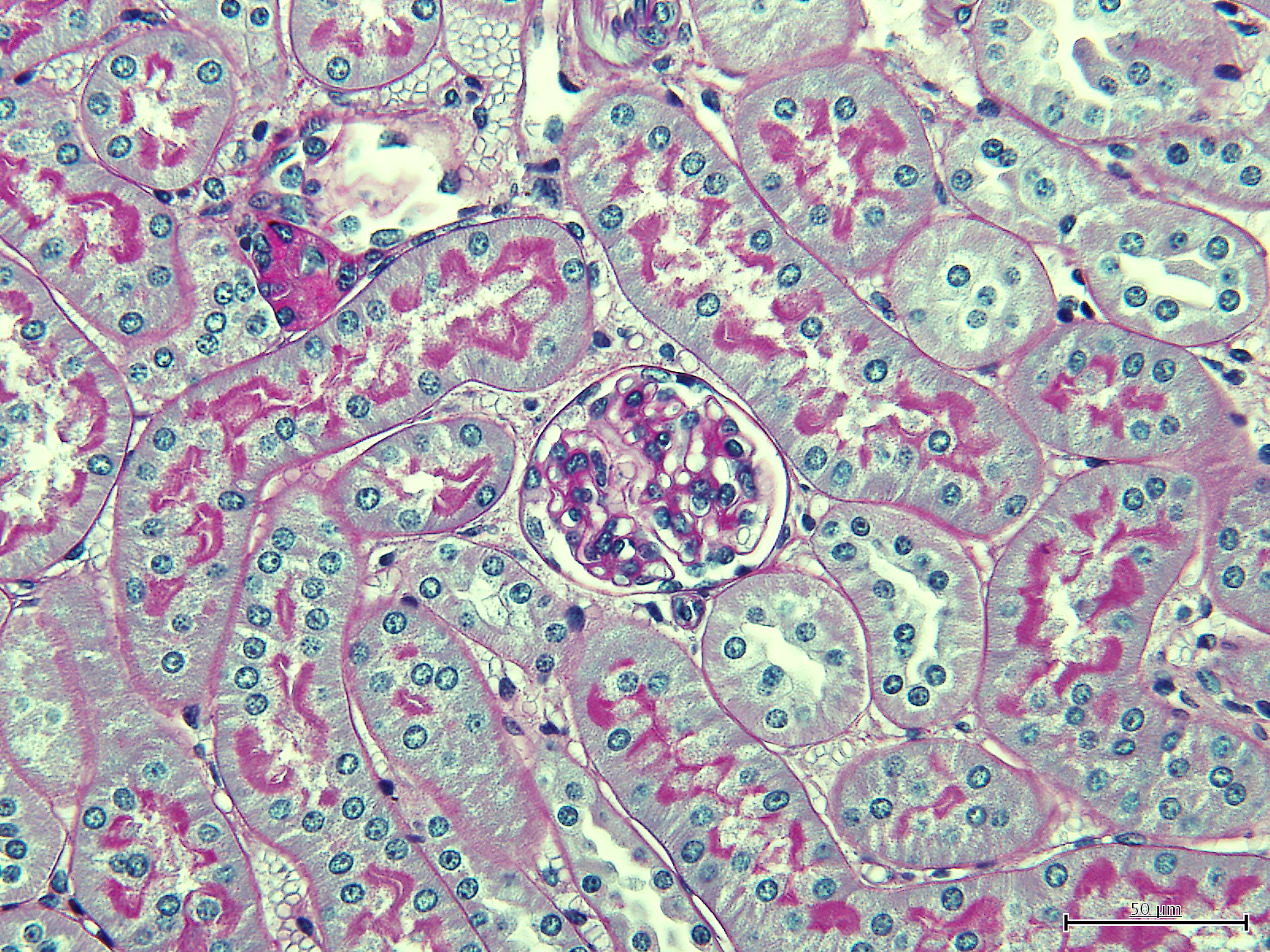

Supplement: Supplementary file 3 — Source data Fig. 1 [file 44318_2026_759_MOESM3_ESM.zip › SD Figure 1/1I/Flox_HighMagnification.tif]

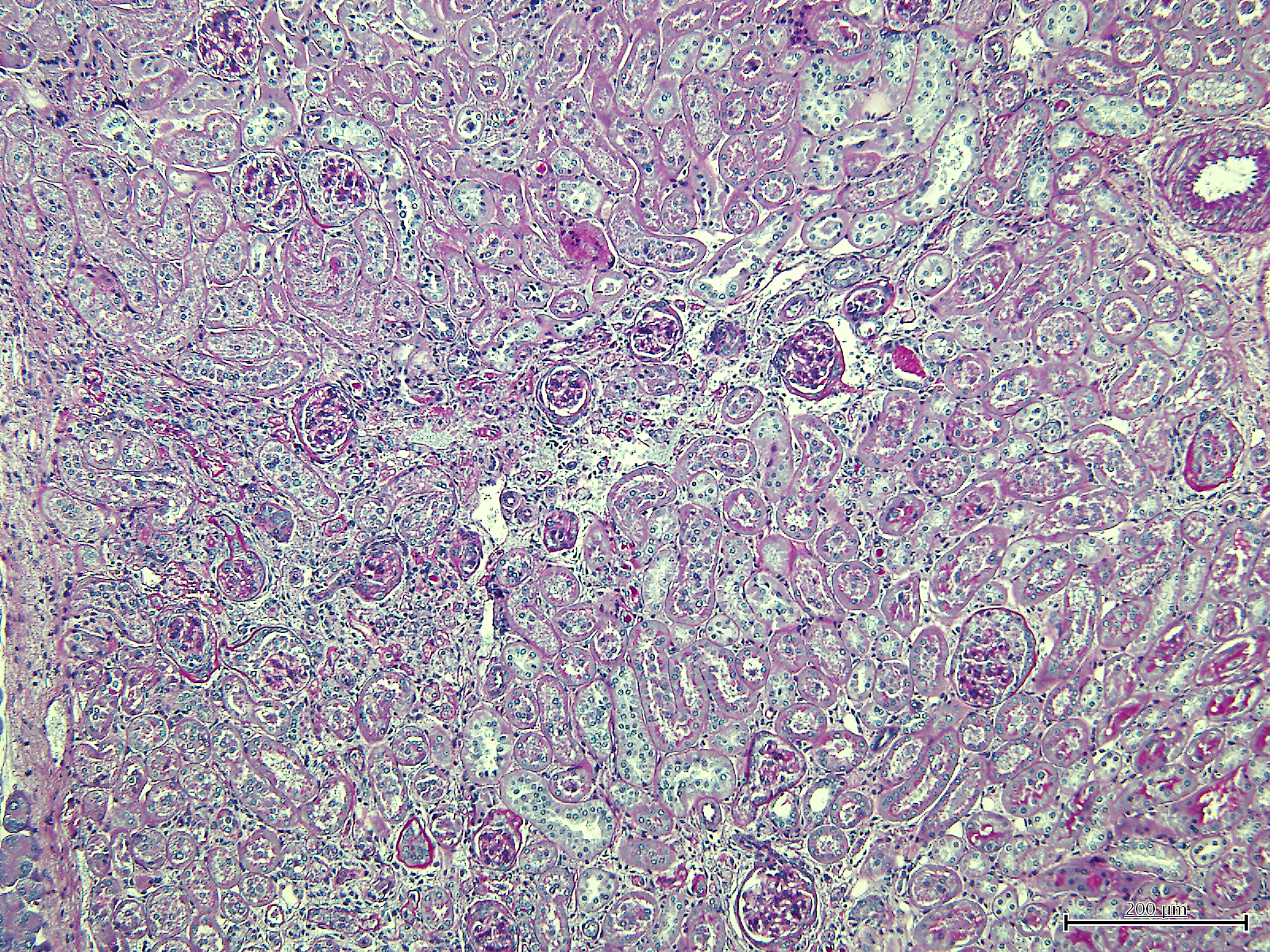

Supplement: Supplementary file 3 — Source data Fig. 1 [file 44318_2026_759_MOESM3_ESM.zip › SD Figure 1/1I/Systemic-Cdkal1 KO_MedumMagnification.tif]

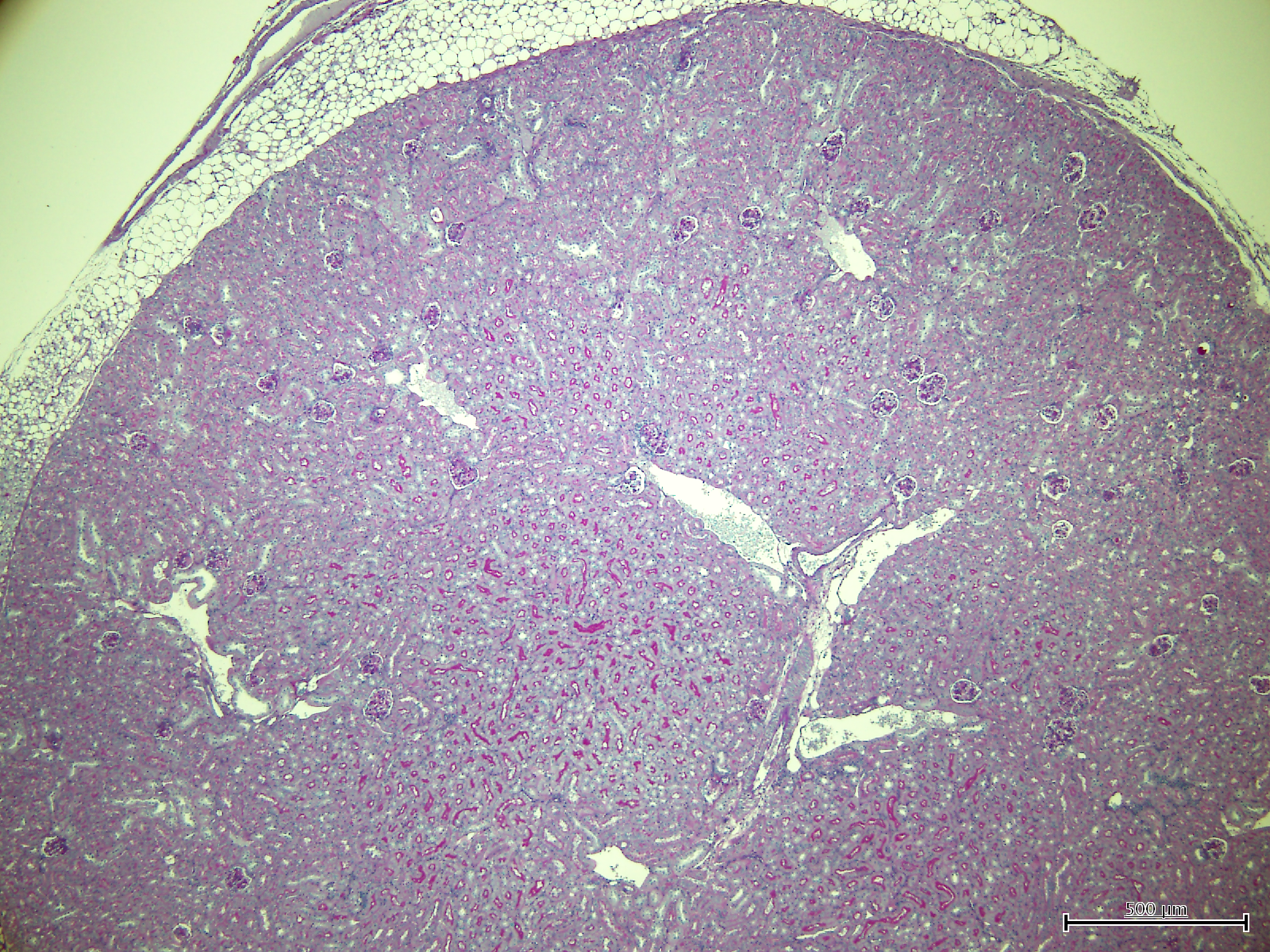

Supplement: Supplementary file 3 — Source data Fig. 1 [file 44318_2026_759_MOESM3_ESM.zip › SD Figure 1/1I/Flox_LowMagnification.TIF]

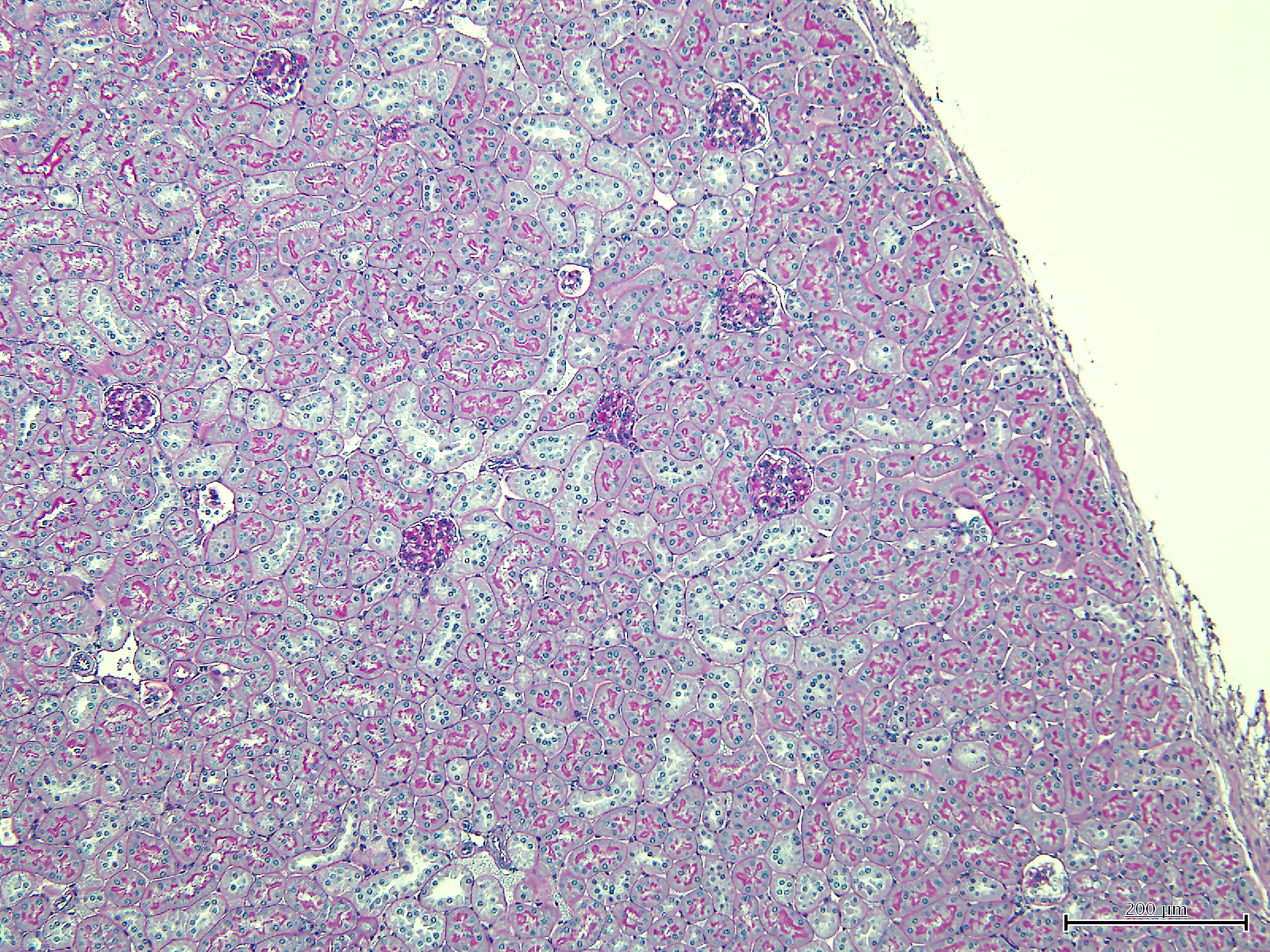

Supplement: Supplementary file 3 — Source data Fig. 1 [file 44318_2026_759_MOESM3_ESM.zip › SD Figure 1/1I/Flox_MediumMagnification.TIF]

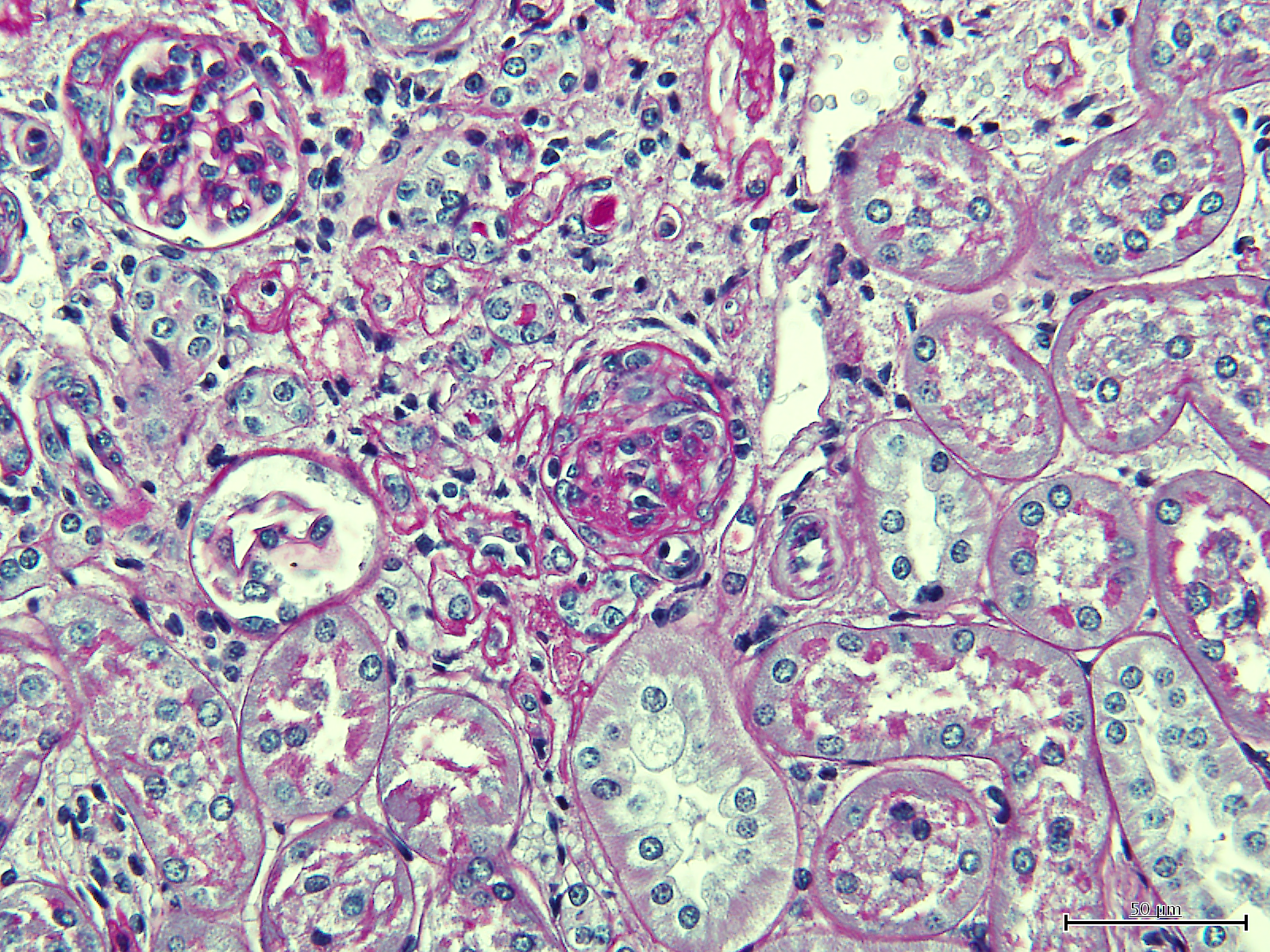

Supplement: Supplementary file 3 — Source data Fig. 1 [file 44318_2026_759_MOESM3_ESM.zip › SD Figure 1/1I/Systemic-Cdkal1 KO_HighMagnification.tif]

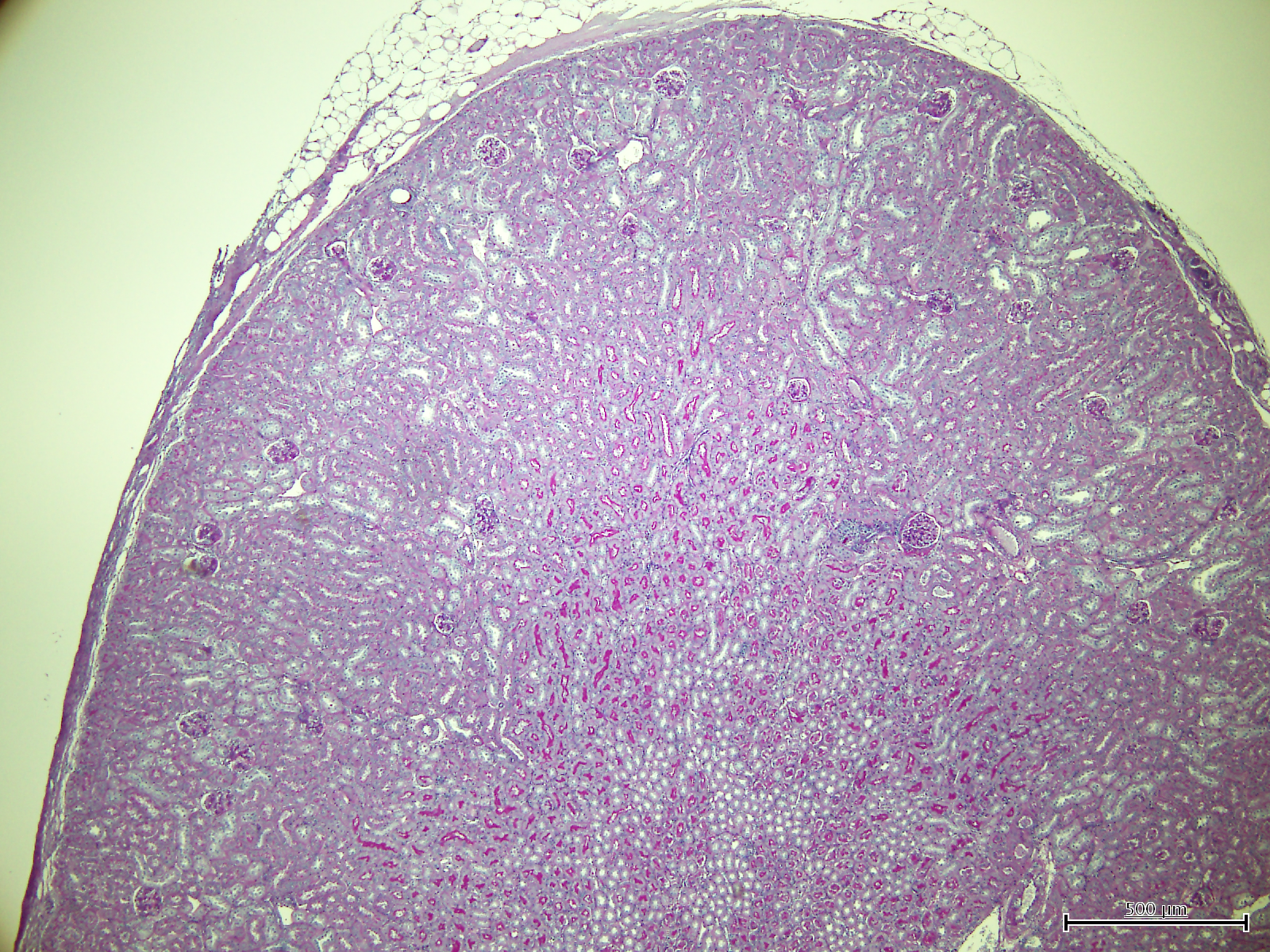

Supplement: Supplementary file 3 — Source data Fig. 1 [file 44318_2026_759_MOESM3_ESM.zip › SD Figure 1/1I/Systemic-Cdkal1 KO_LowMagnification.tif]

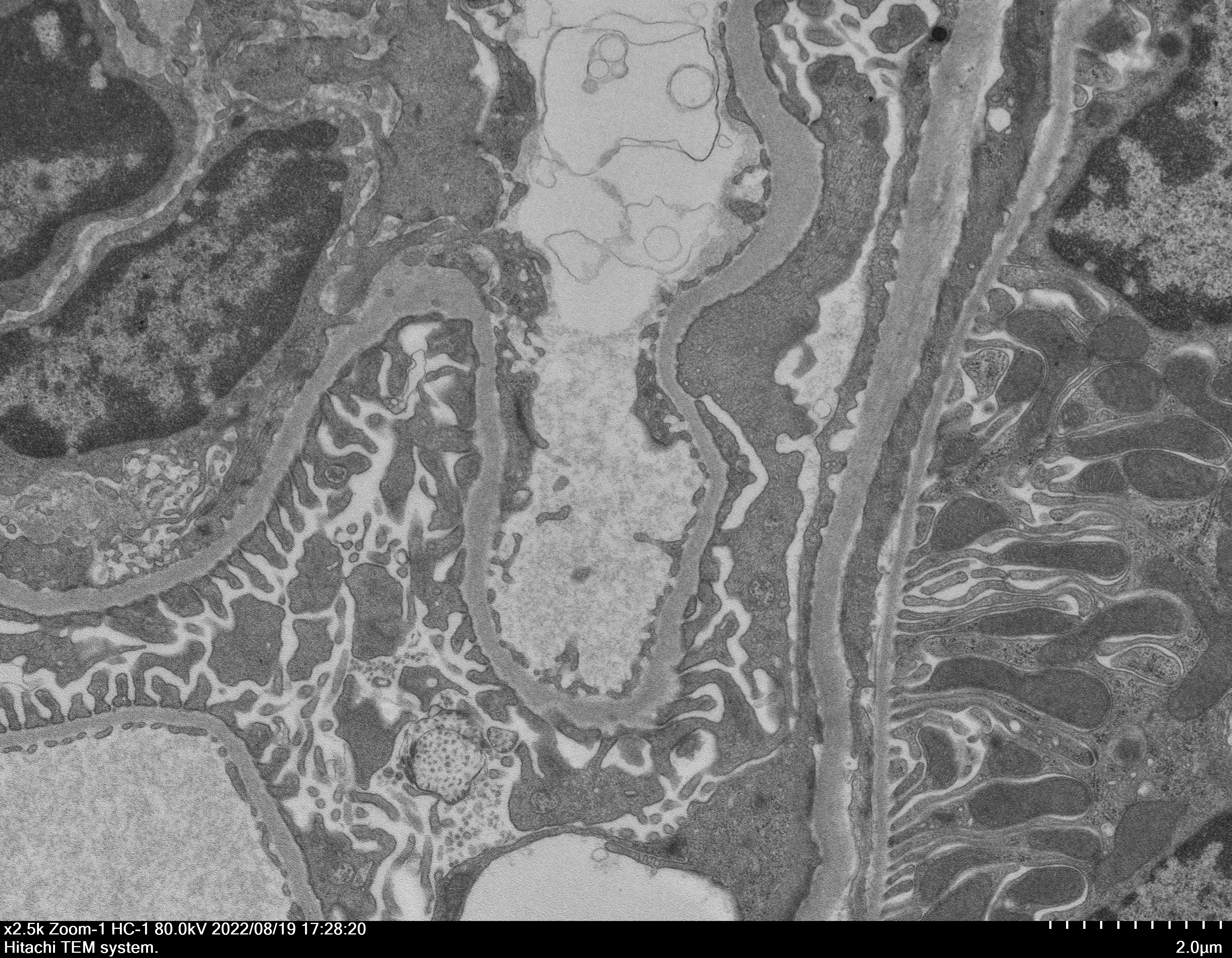

Supplement: Supplementary file 3 — Source data Fig. 1 [file 44318_2026_759_MOESM3_ESM.zip › SD Figure 1/1J/Systemic Cdkal1 KO_2.5k.tif]

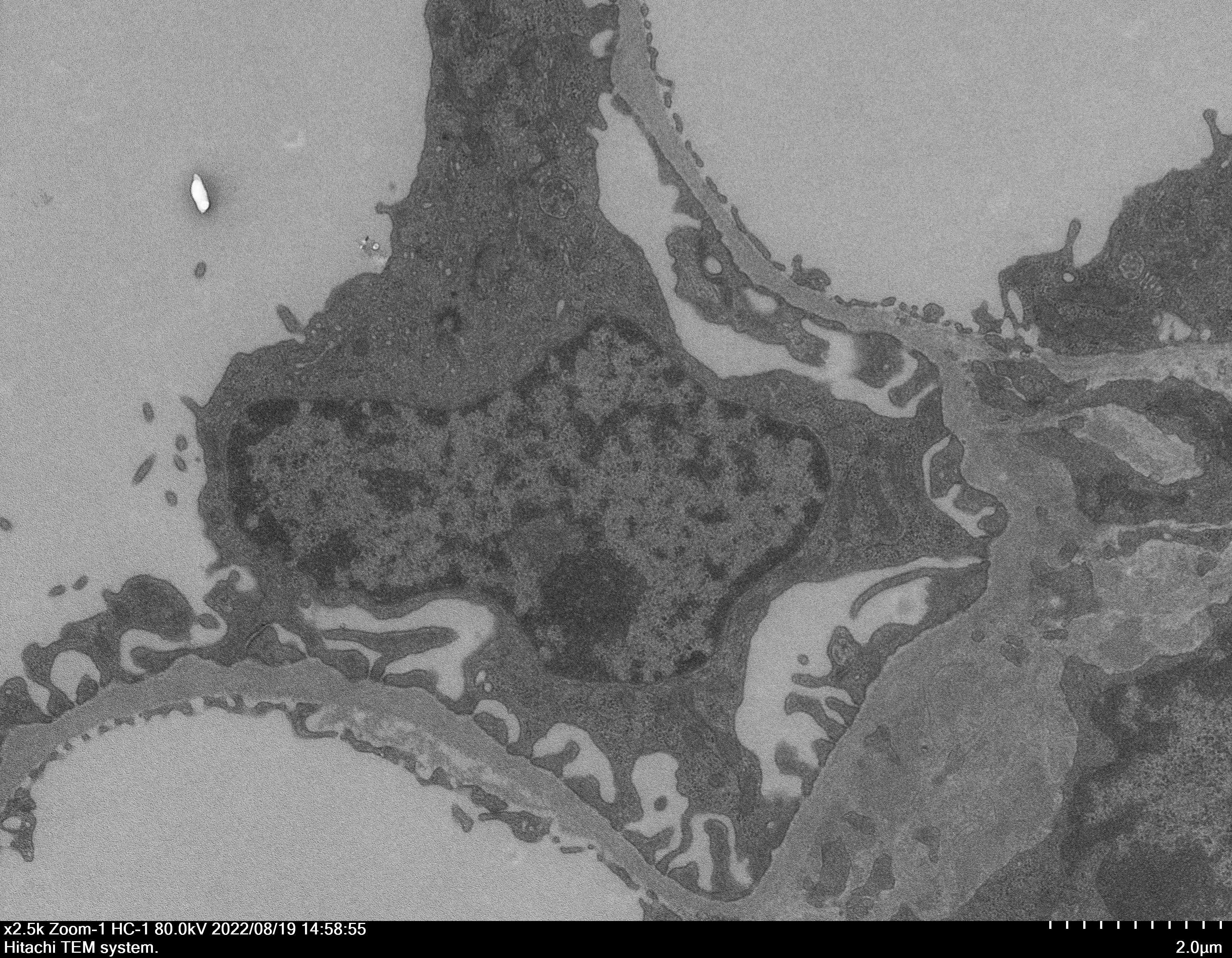

Supplement: Supplementary file 3 — Source data Fig. 1 [file 44318_2026_759_MOESM3_ESM.zip › SD Figure 1/1J/Flox_2.5k.tif]

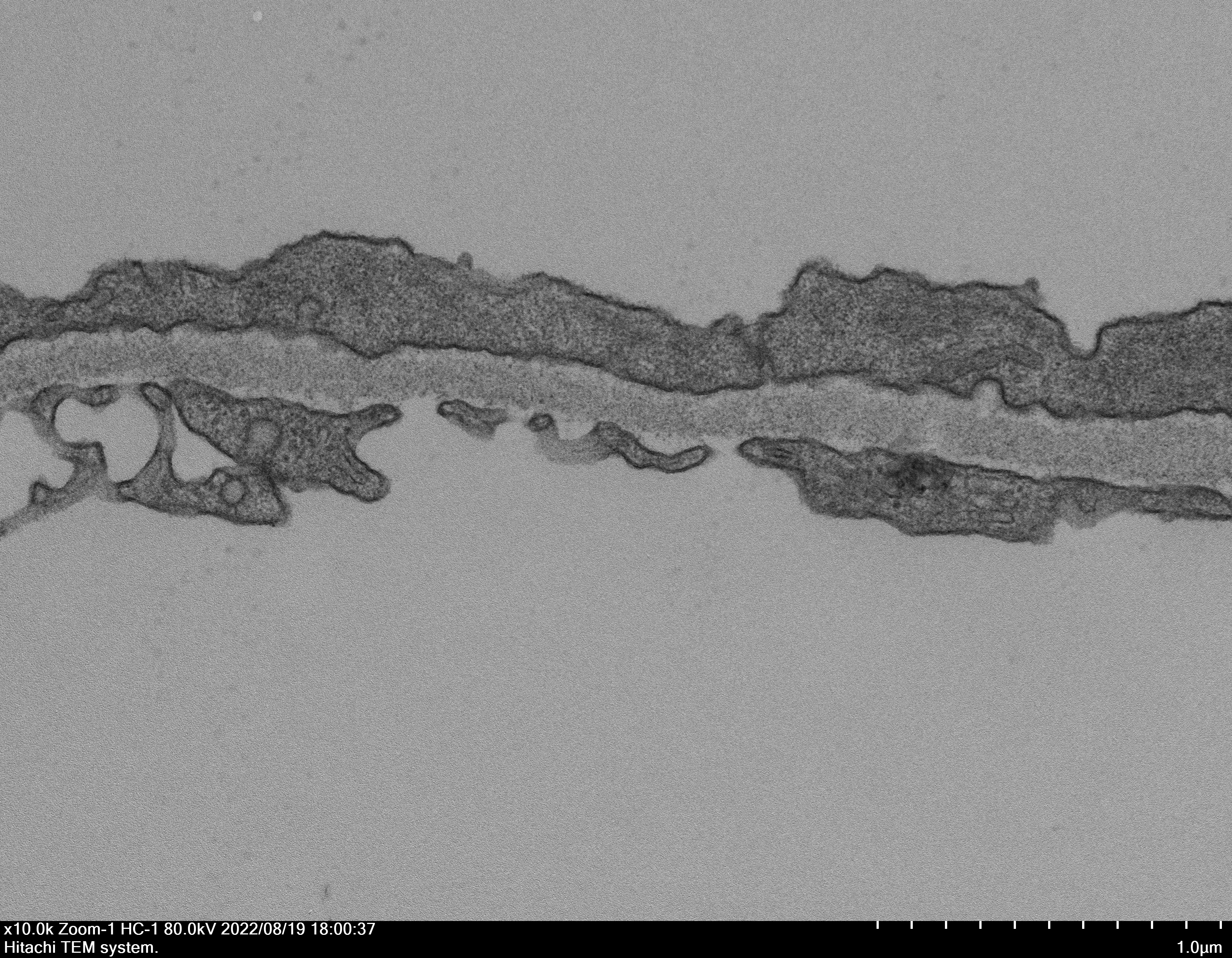

Supplement: Supplementary file 3 — Source data Fig. 1 [file 44318_2026_759_MOESM3_ESM.zip › SD Figure 1/1J/Systemic Cdkal1 KO_10.0k.tif]

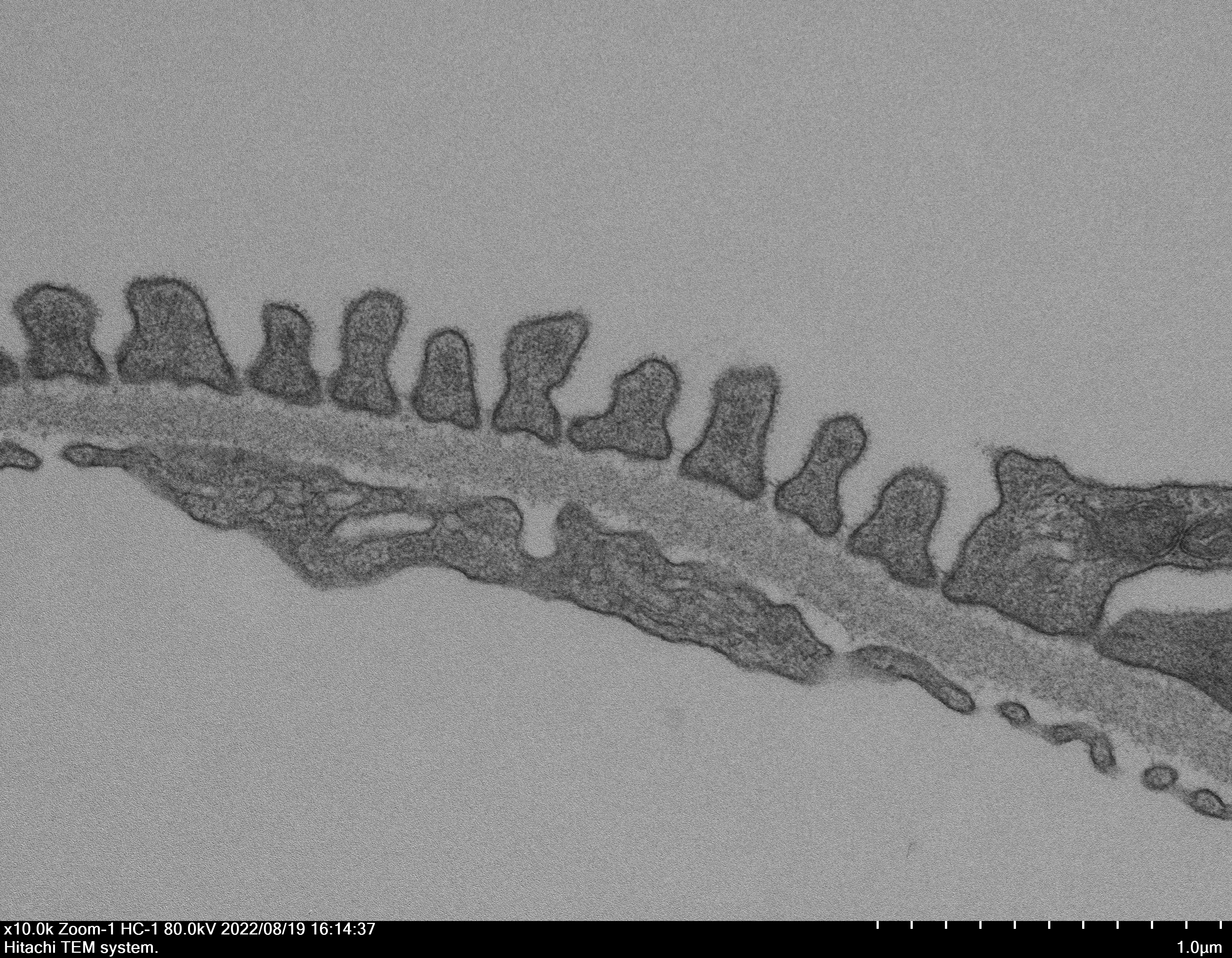

Supplement: Supplementary file 3 — Source data Fig. 1 [file 44318_2026_759_MOESM3_ESM.zip › SD Figure 1/1J/Flox_10.0k.tif]

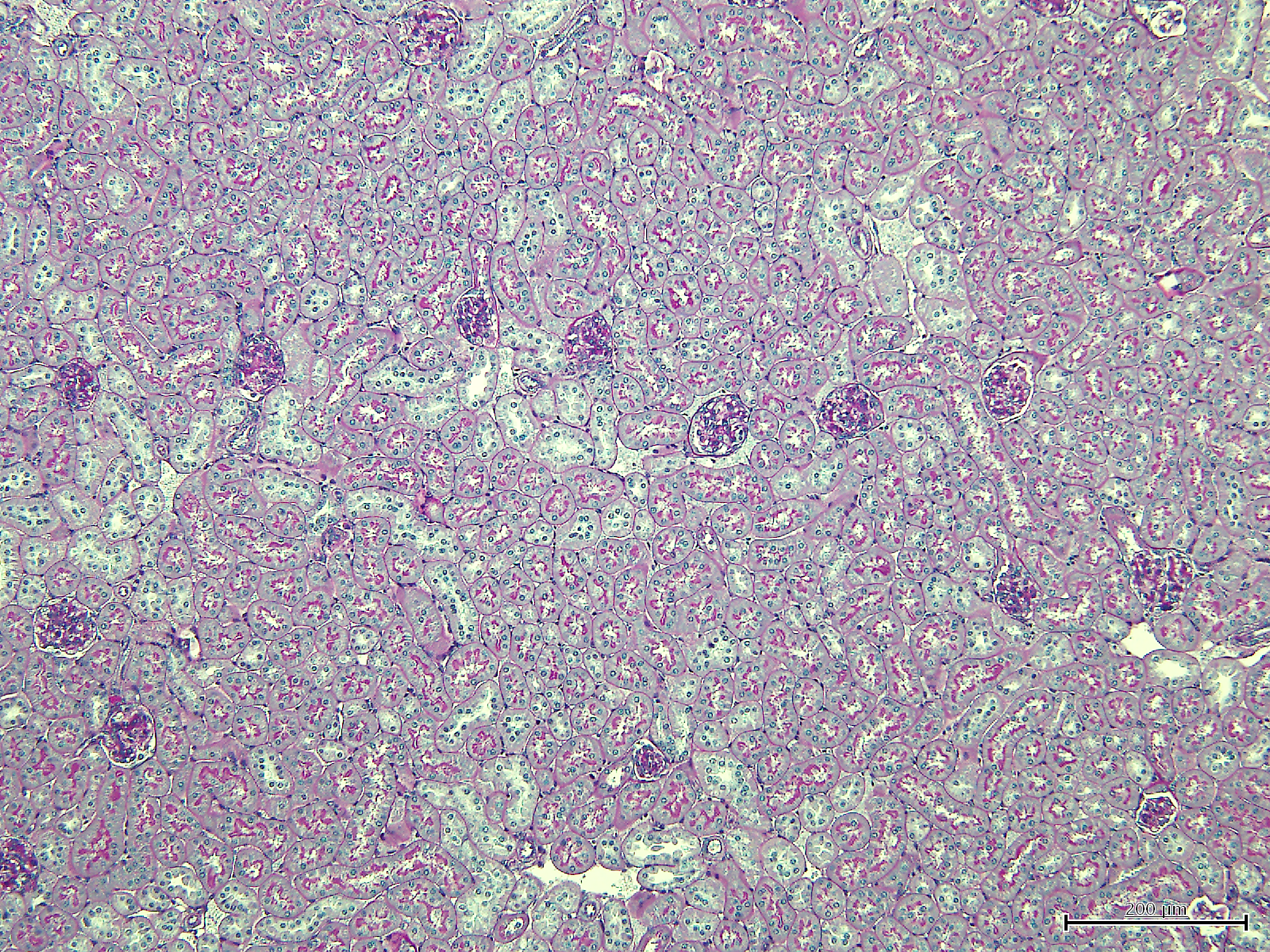

Supplement: Supplementary file 4 — Source data Fig. 2 [file 44318_2026_759_MOESM4_ESM.zip › SD Figure 2/2G/Flox_MediumMagnificant.TIF]

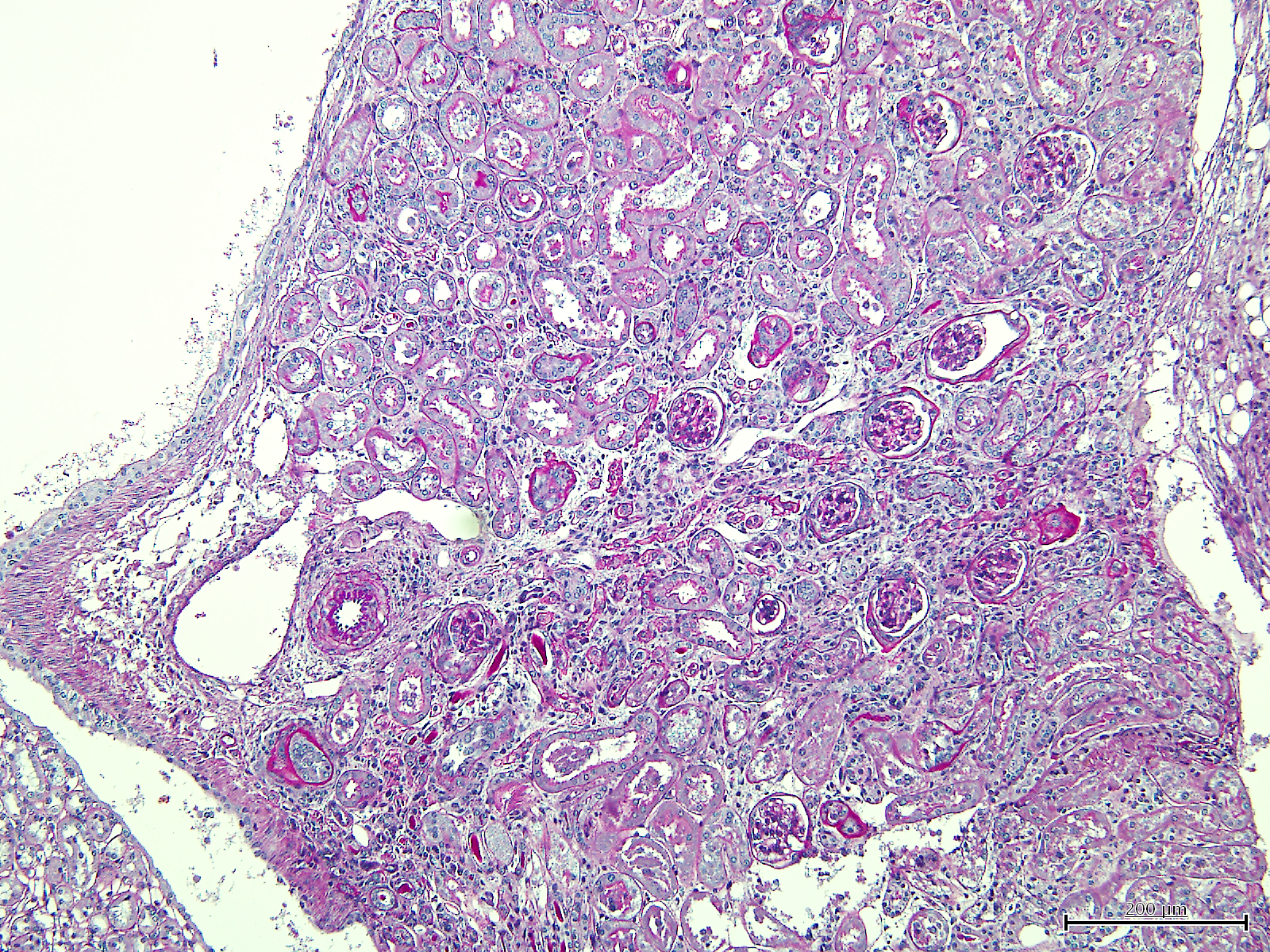

Supplement: Supplementary file 4 — Source data Fig. 2 [file 44318_2026_759_MOESM4_ESM.zip › SD Figure 2/2G/Podocyte-specific Cdkal1 KO_MediumMagnification.TIF]

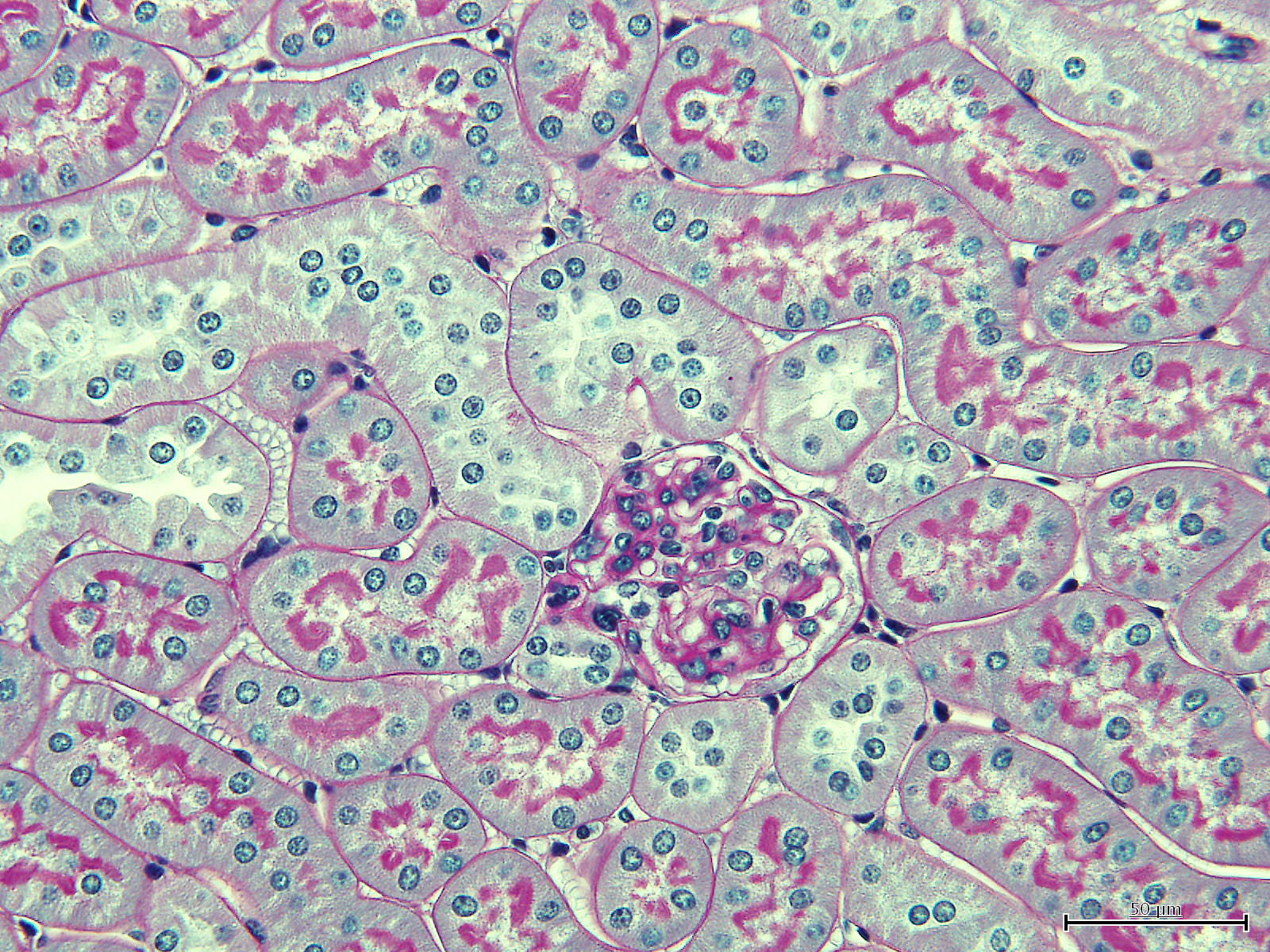

Supplement: Supplementary file 4 — Source data Fig. 2 [file 44318_2026_759_MOESM4_ESM.zip › SD Figure 2/2G/Flox_HighMagnification.tif]

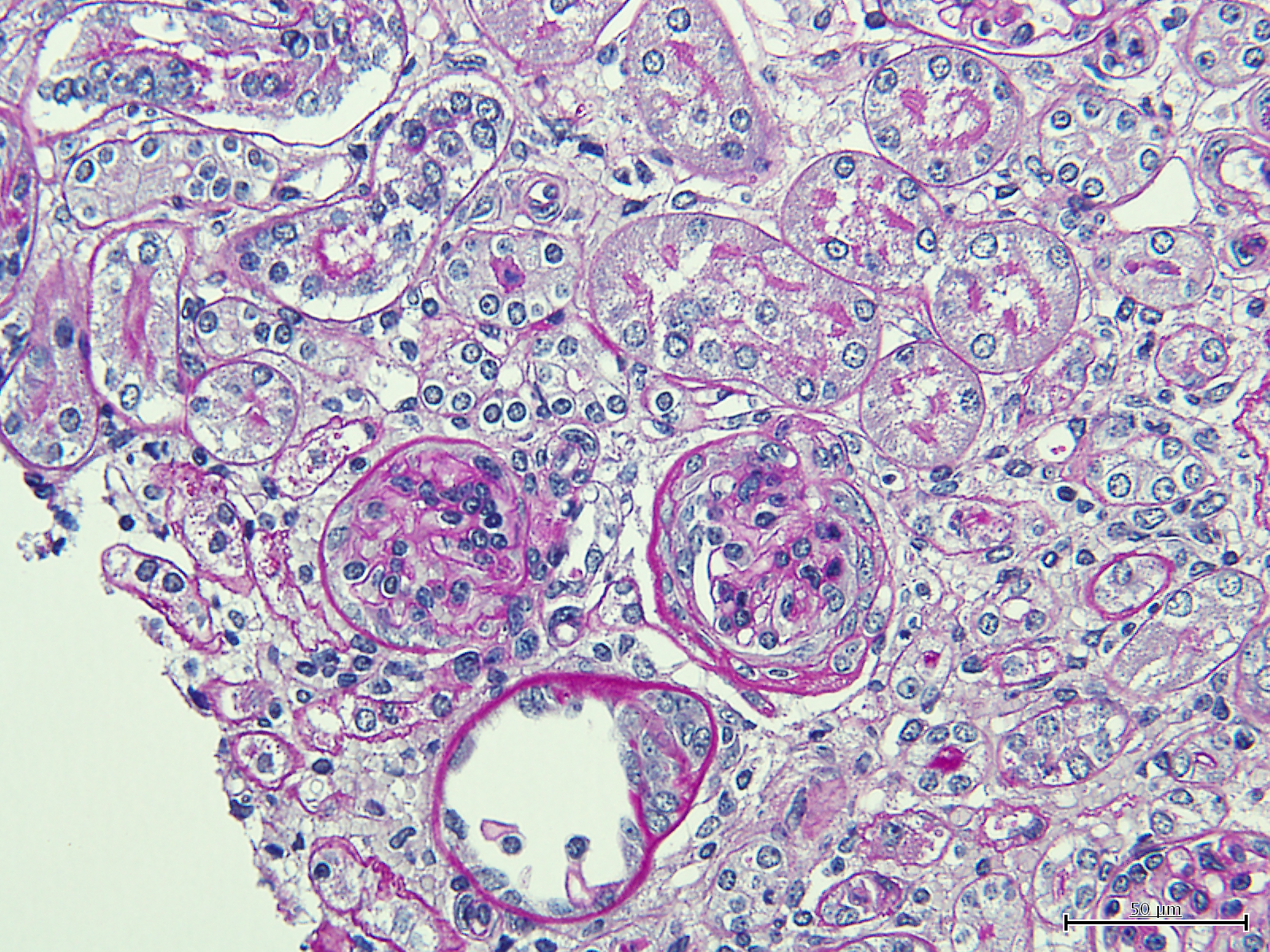

Supplement: Supplementary file 4 — Source data Fig. 2 [file 44318_2026_759_MOESM4_ESM.zip › SD Figure 2/2G/Podocyte-specific Cdkal1 KO_HighMagnification.tif]

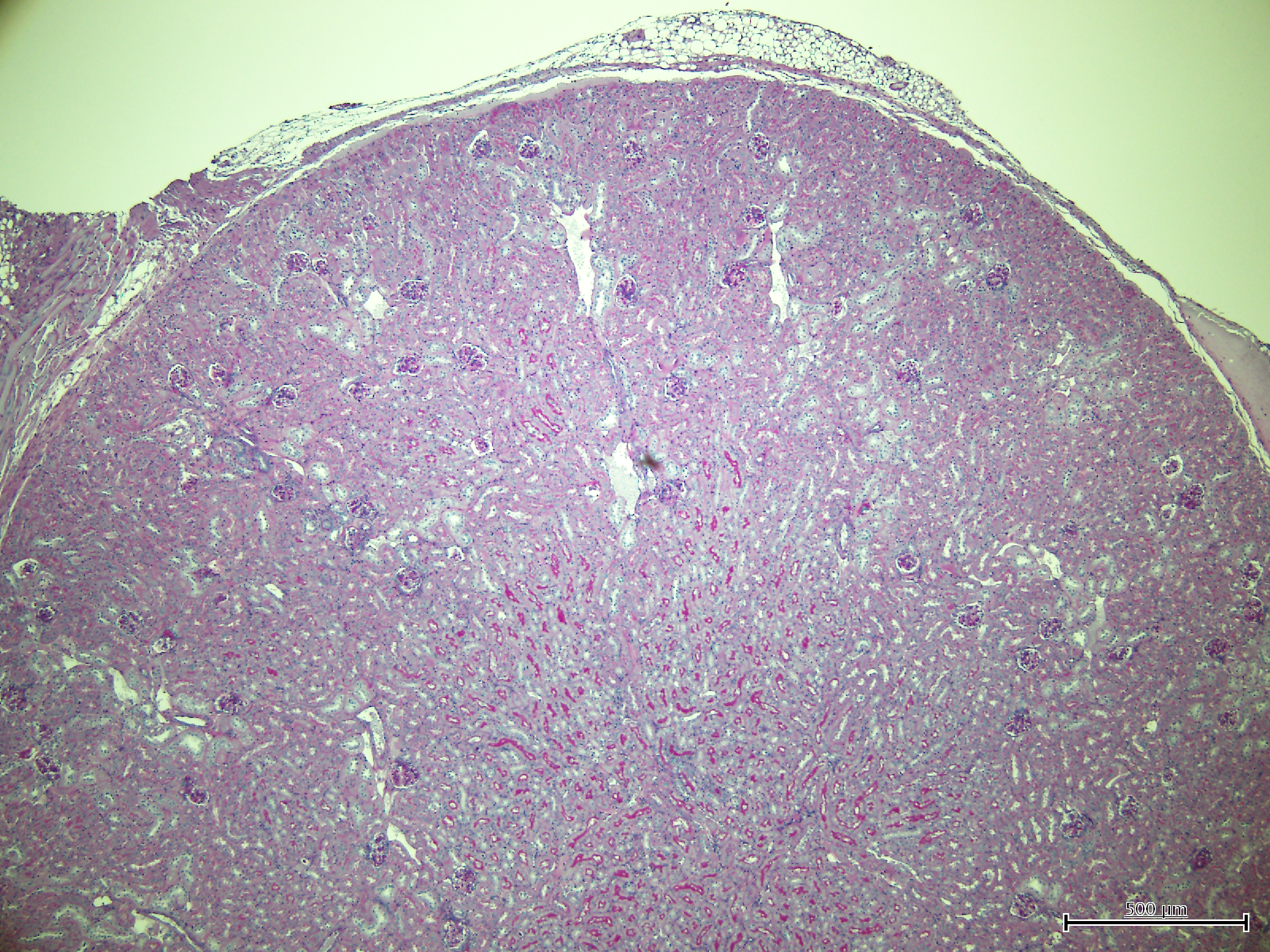

Supplement: Supplementary file 4 — Source data Fig. 2 [file 44318_2026_759_MOESM4_ESM.zip › SD Figure 2/2G/Flox_LowMagnification.TIF]

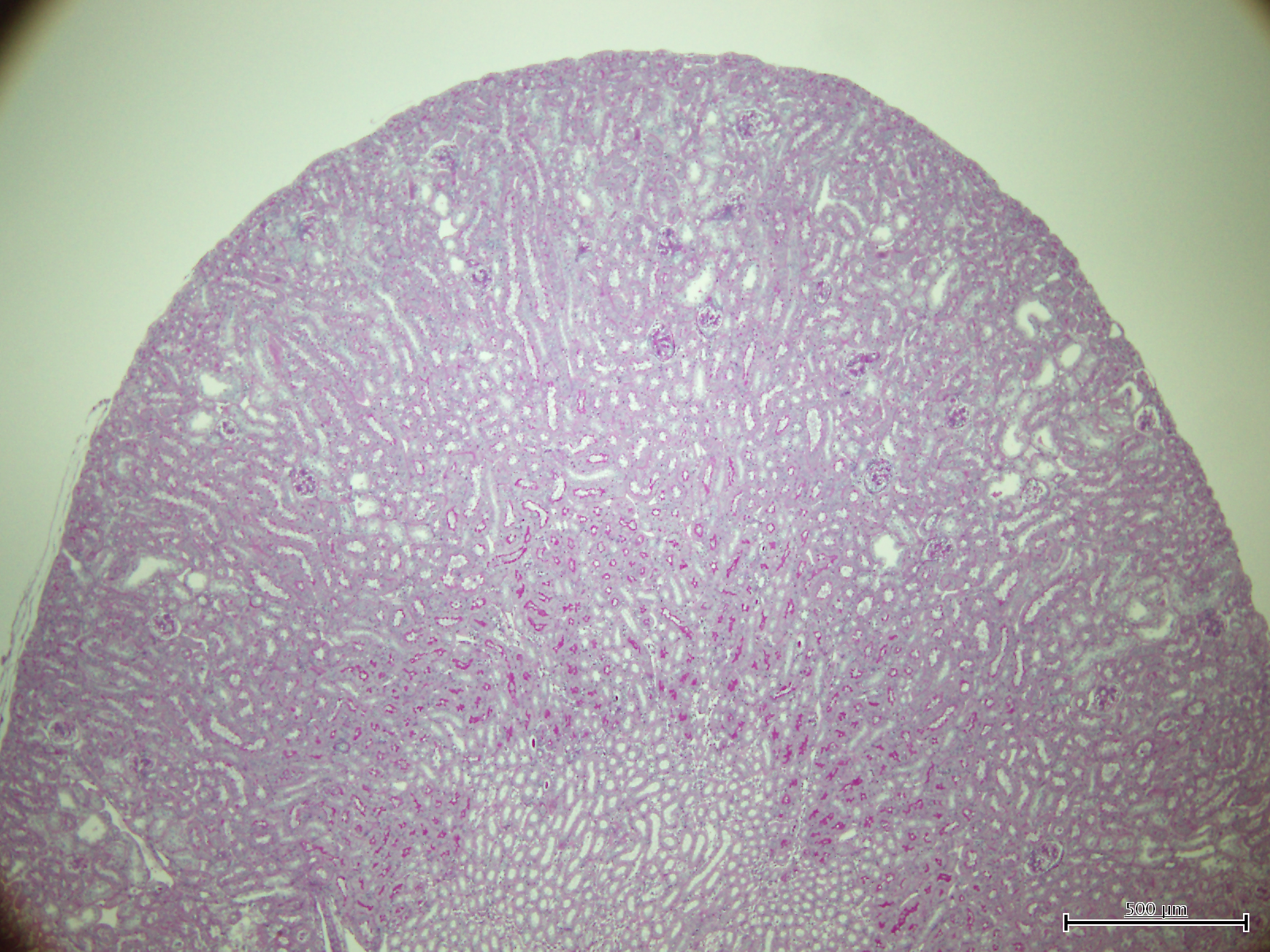

Supplement: Supplementary file 4 — Source data Fig. 2 [file 44318_2026_759_MOESM4_ESM.zip › SD Figure 2/2G/Podocyte-specific Cdkal1 KO_LowMagnification.tif]

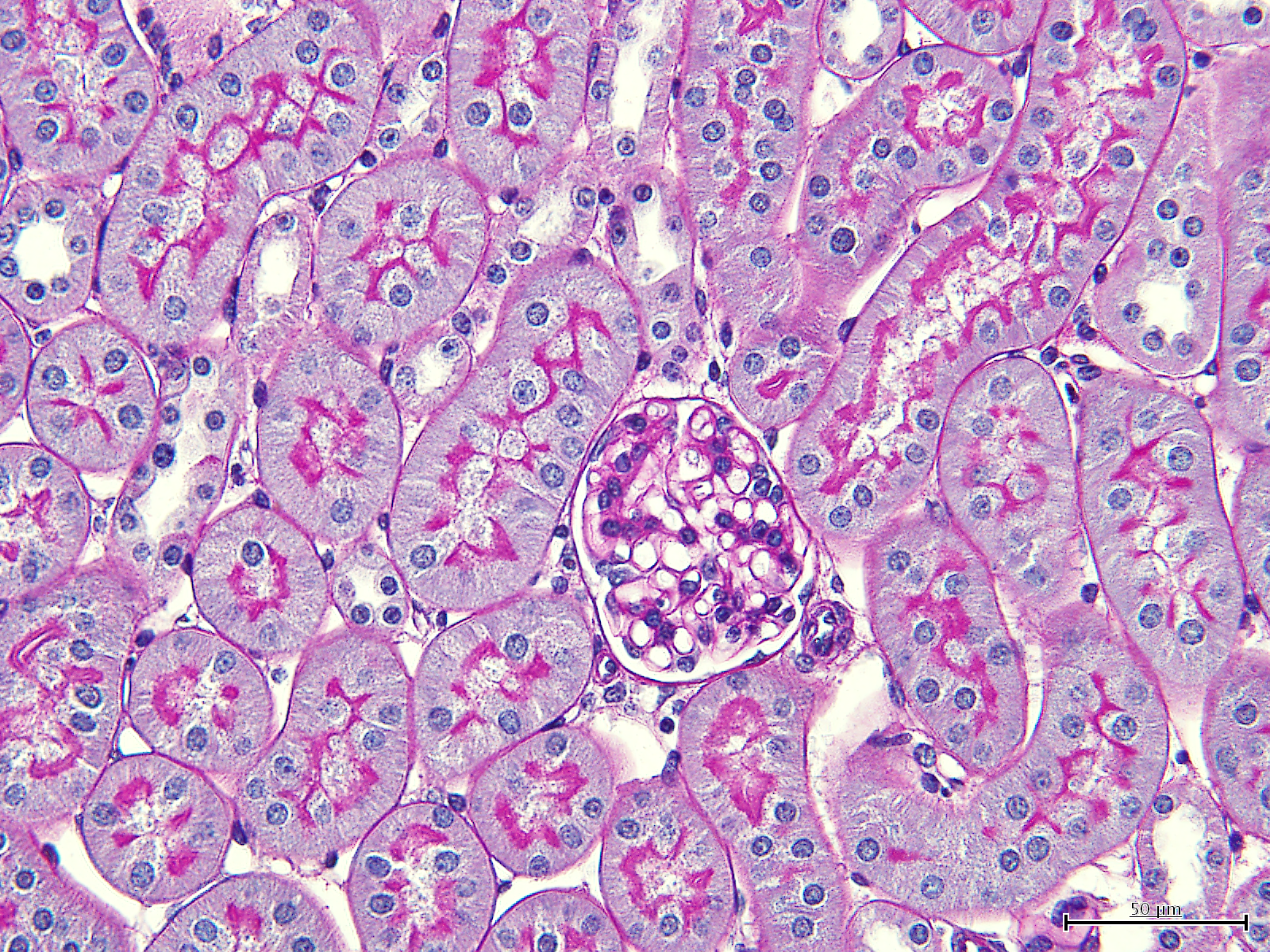

Supplement: Supplementary file 4 — Source data Fig. 2 [file 44318_2026_759_MOESM4_ESM.zip › SD Figure 2/2D/Flox_HighMagnification.TIF]

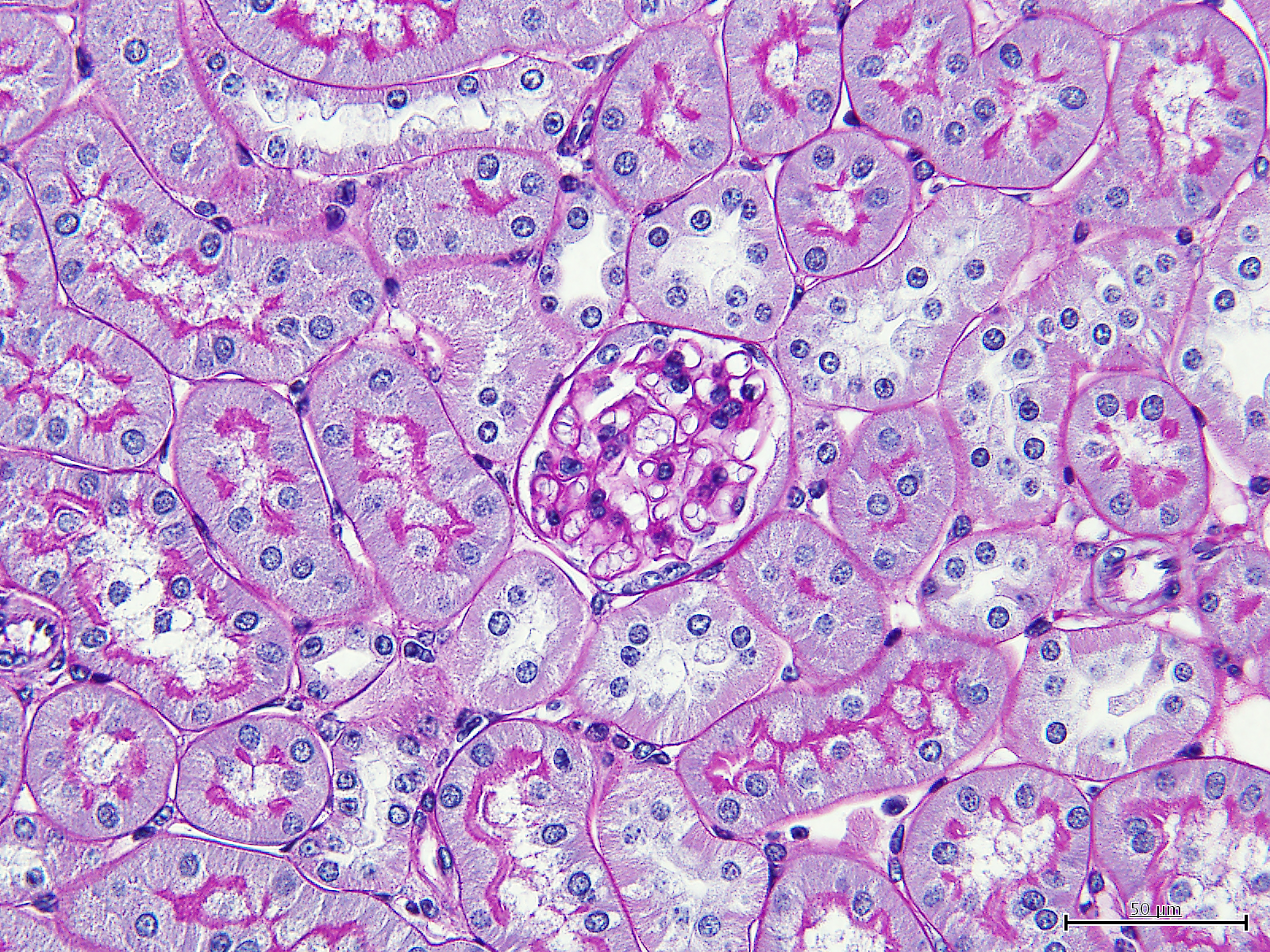

Supplement: Supplementary file 4 — Source data Fig. 2 [file 44318_2026_759_MOESM4_ESM.zip › SD Figure 2/2D/Podocyte-specific Cdkal1 KO_HighMagnification.TIF]

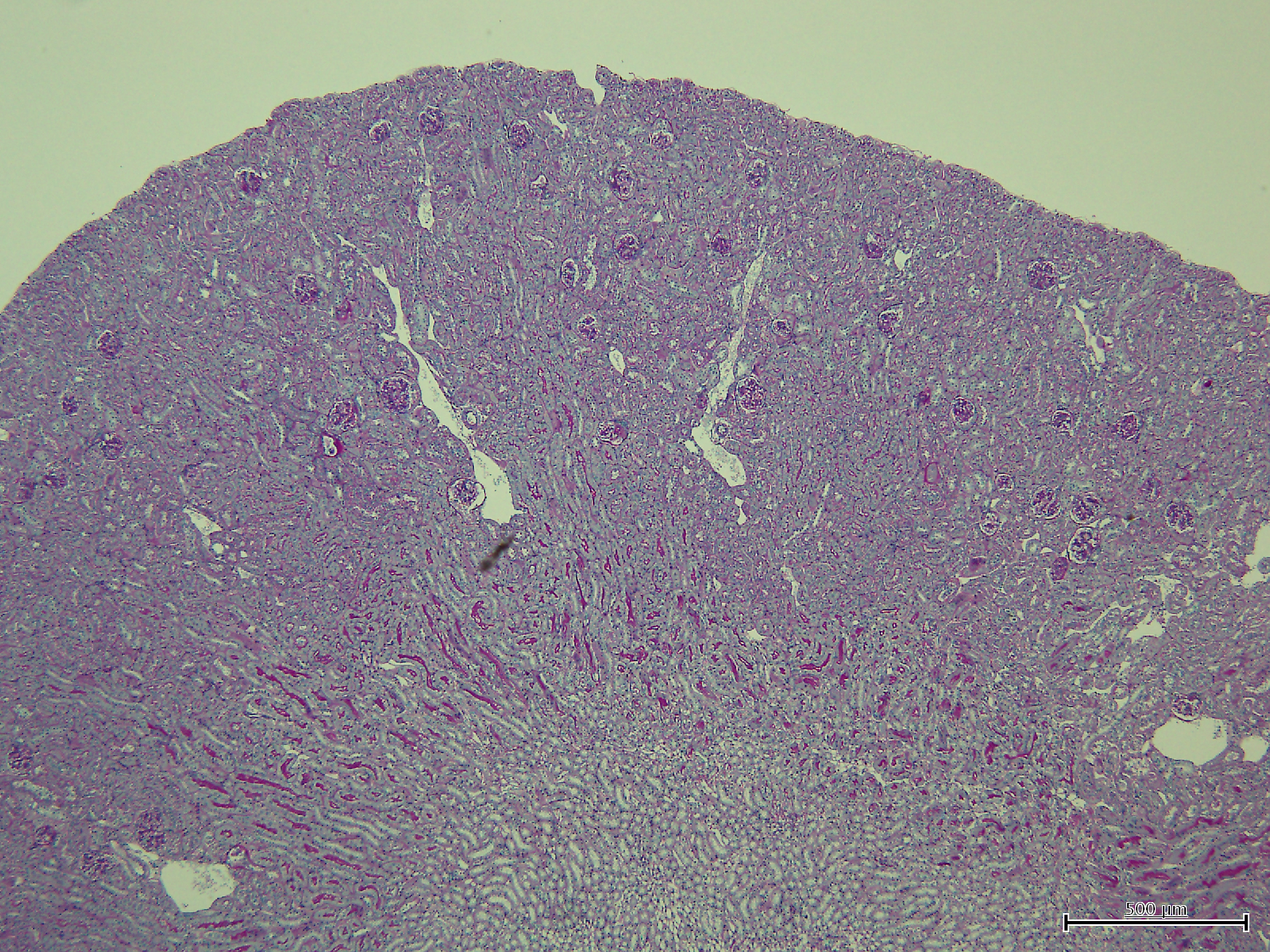

Supplement: Supplementary file 4 — Source data Fig. 2 [file 44318_2026_759_MOESM4_ESM.zip › SD Figure 2/2D/Flox_LowMagnification.TIF]

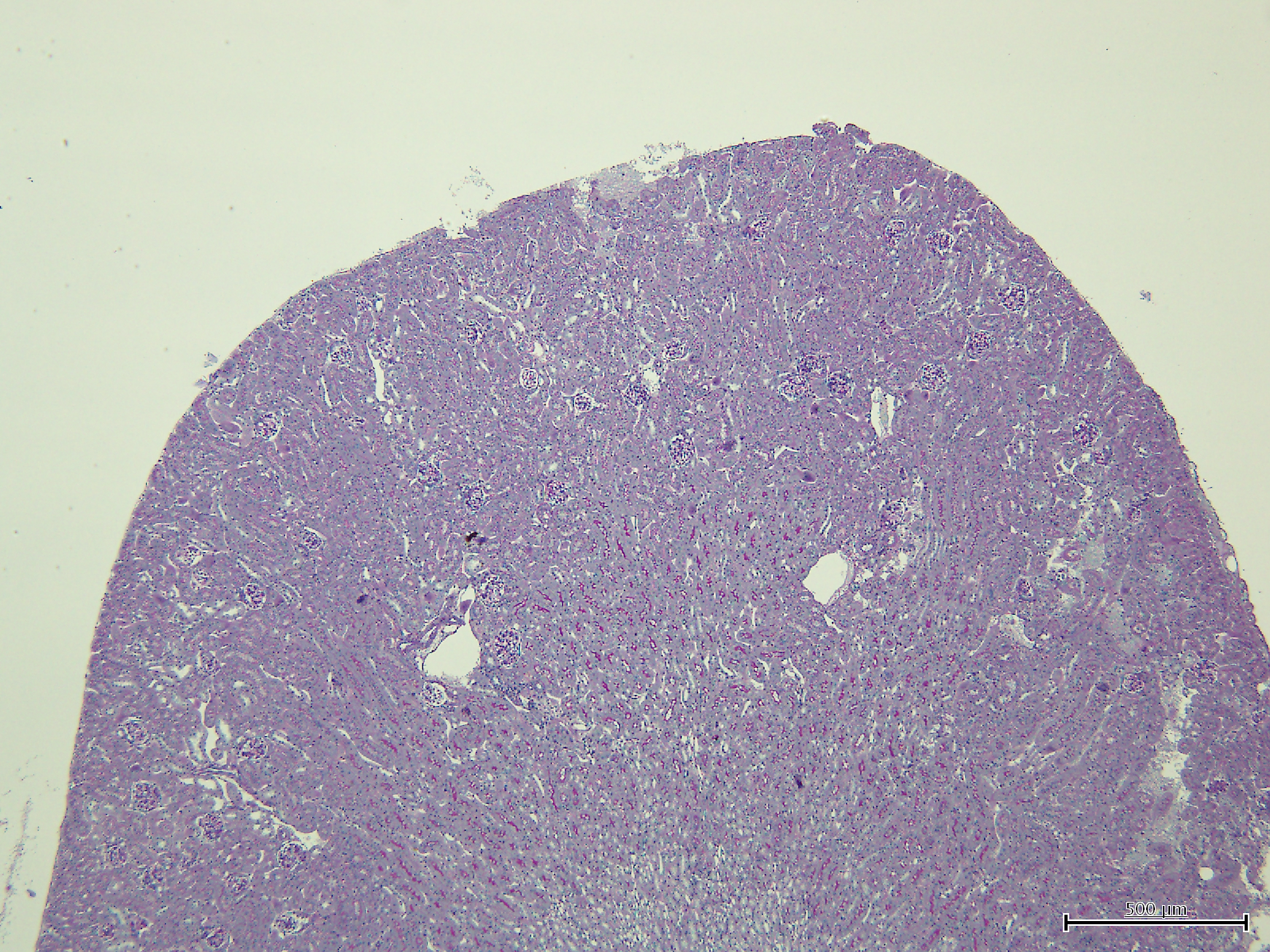

Supplement: Supplementary file 4 — Source data Fig. 2 [file 44318_2026_759_MOESM4_ESM.zip › SD Figure 2/2D/Podocyte-specific Cdkal1 KO_LowMagnification.TIF]

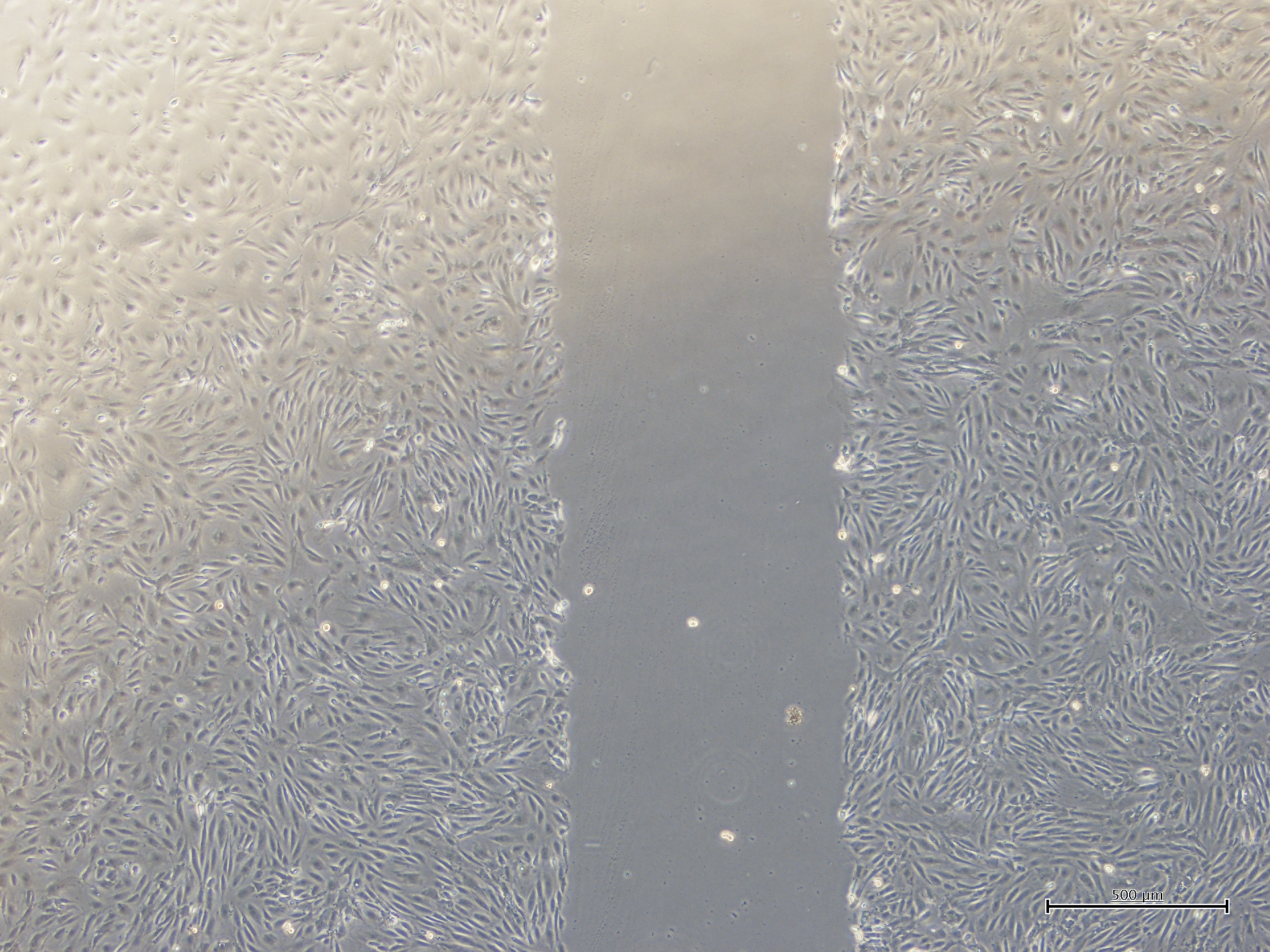

Supplement: Supplementary file 5 — Source data Fig. 3 [file 44318_2026_759_MOESM5_ESM.zip › SD Figure 3/3E/E11 0hr/SgControl .TIF]

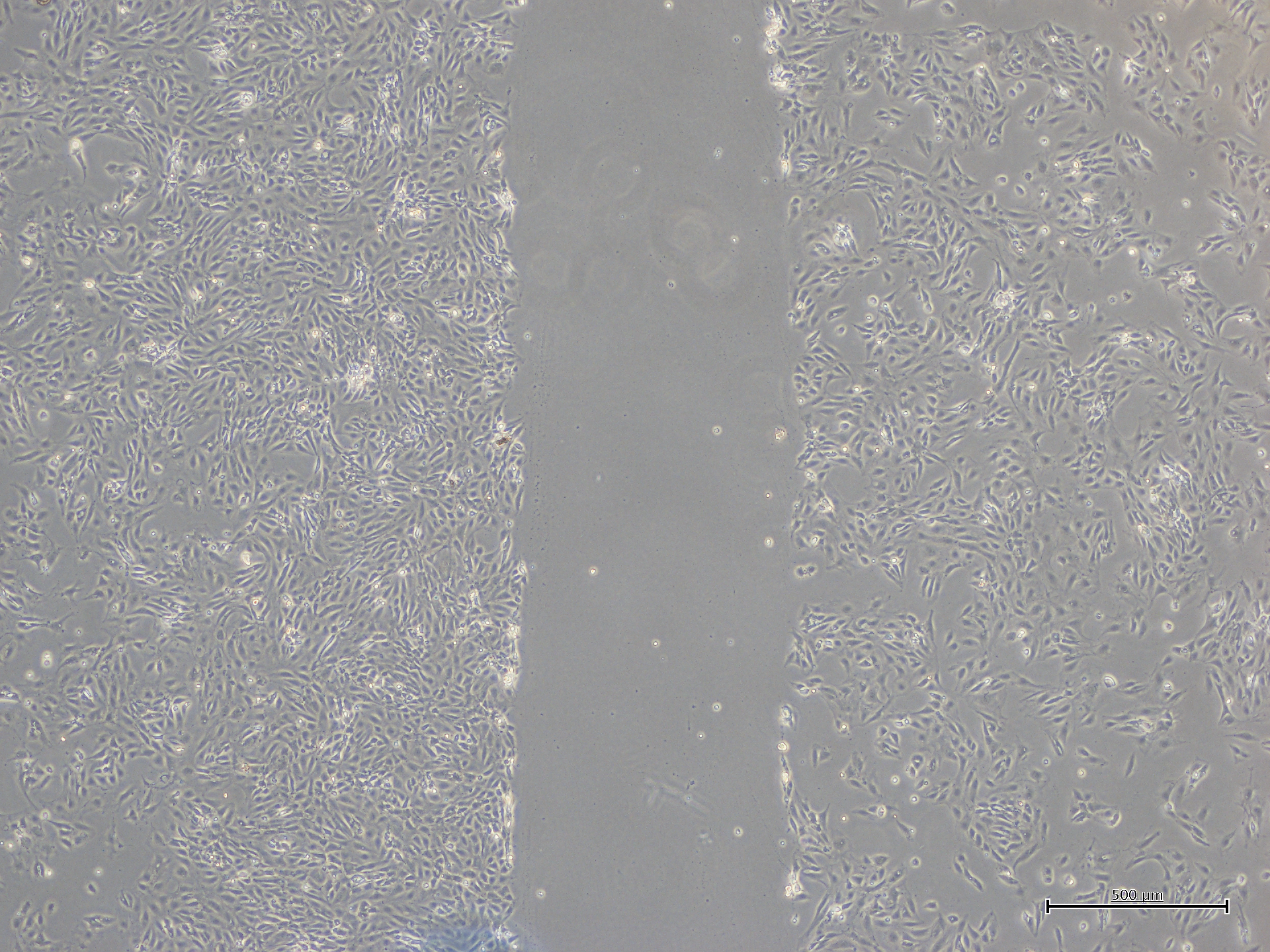

Supplement: Supplementary file 5 — Source data Fig. 3 [file 44318_2026_759_MOESM5_ESM.zip › SD Figure 3/3E/E11 0hr/Cdkal1 KO 2.TIF]

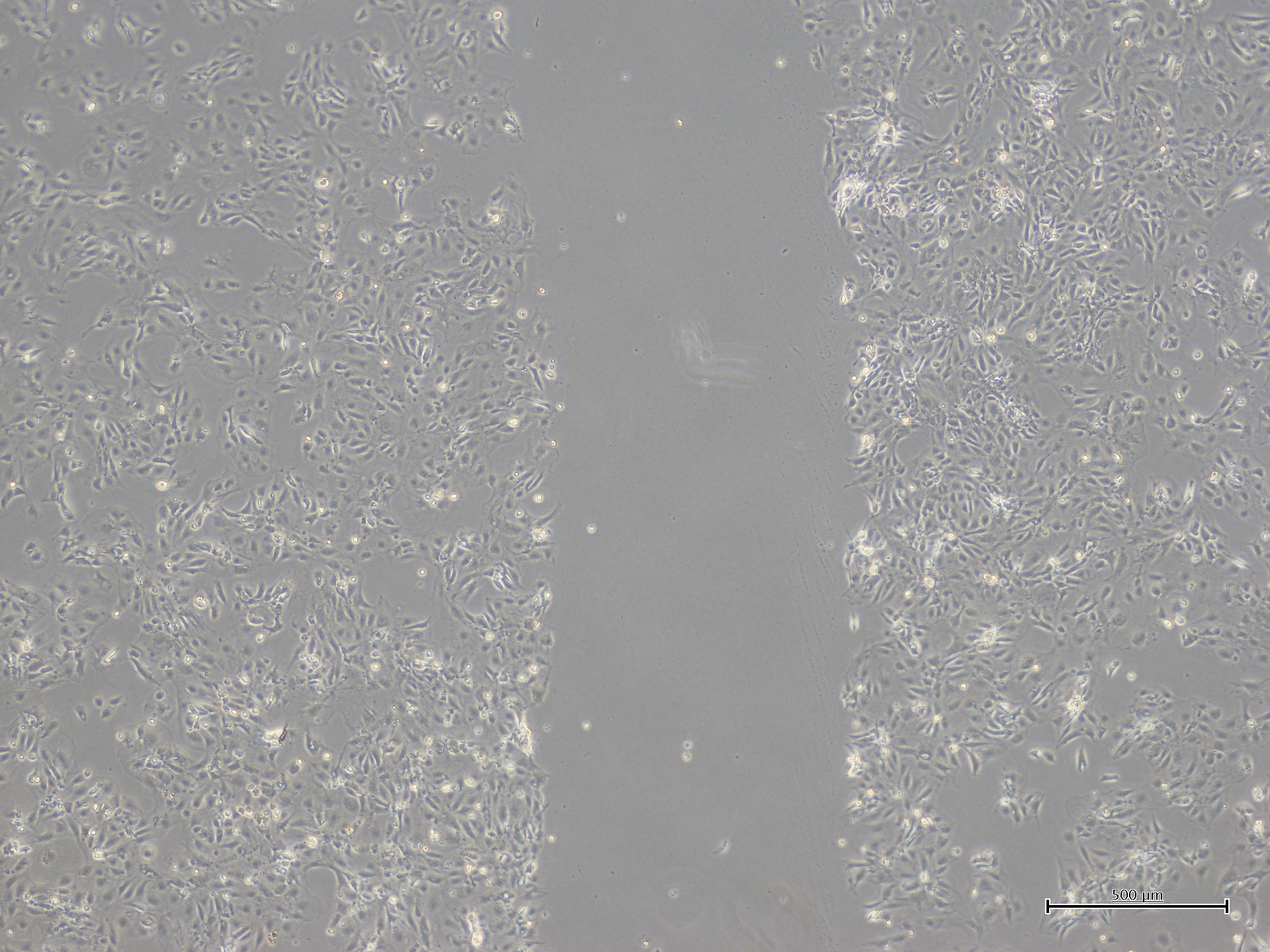

Supplement: Supplementary file 5 — Source data Fig. 3 [file 44318_2026_759_MOESM5_ESM.zip › SD Figure 3/3E/E11 0hr/Cdkal1 KO1.TIF]

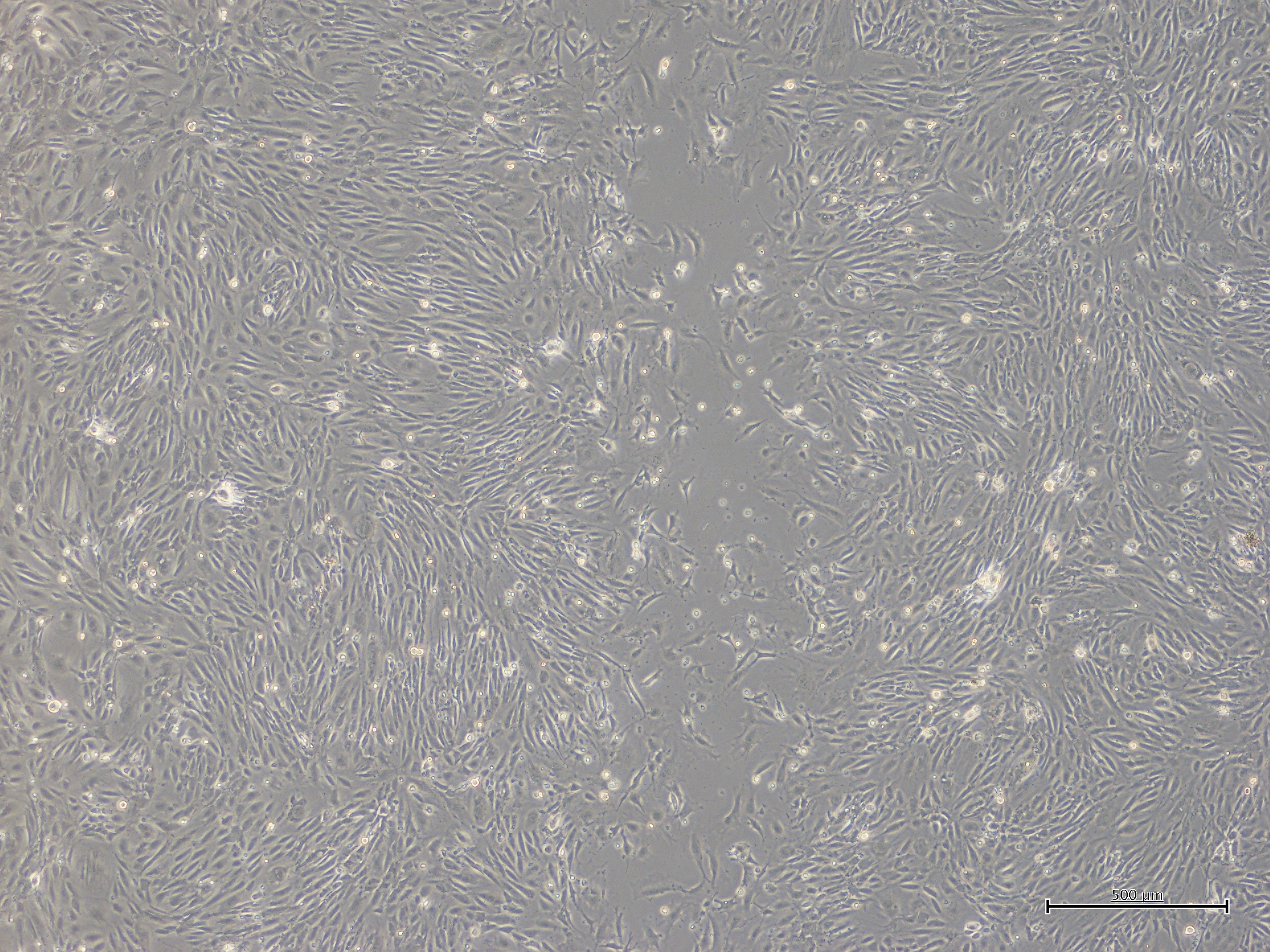

Supplement: Supplementary file 5 — Source data Fig. 3 [file 44318_2026_759_MOESM5_ESM.zip › SD Figure 3/3E/E11 30hr/SgControl .TIF]

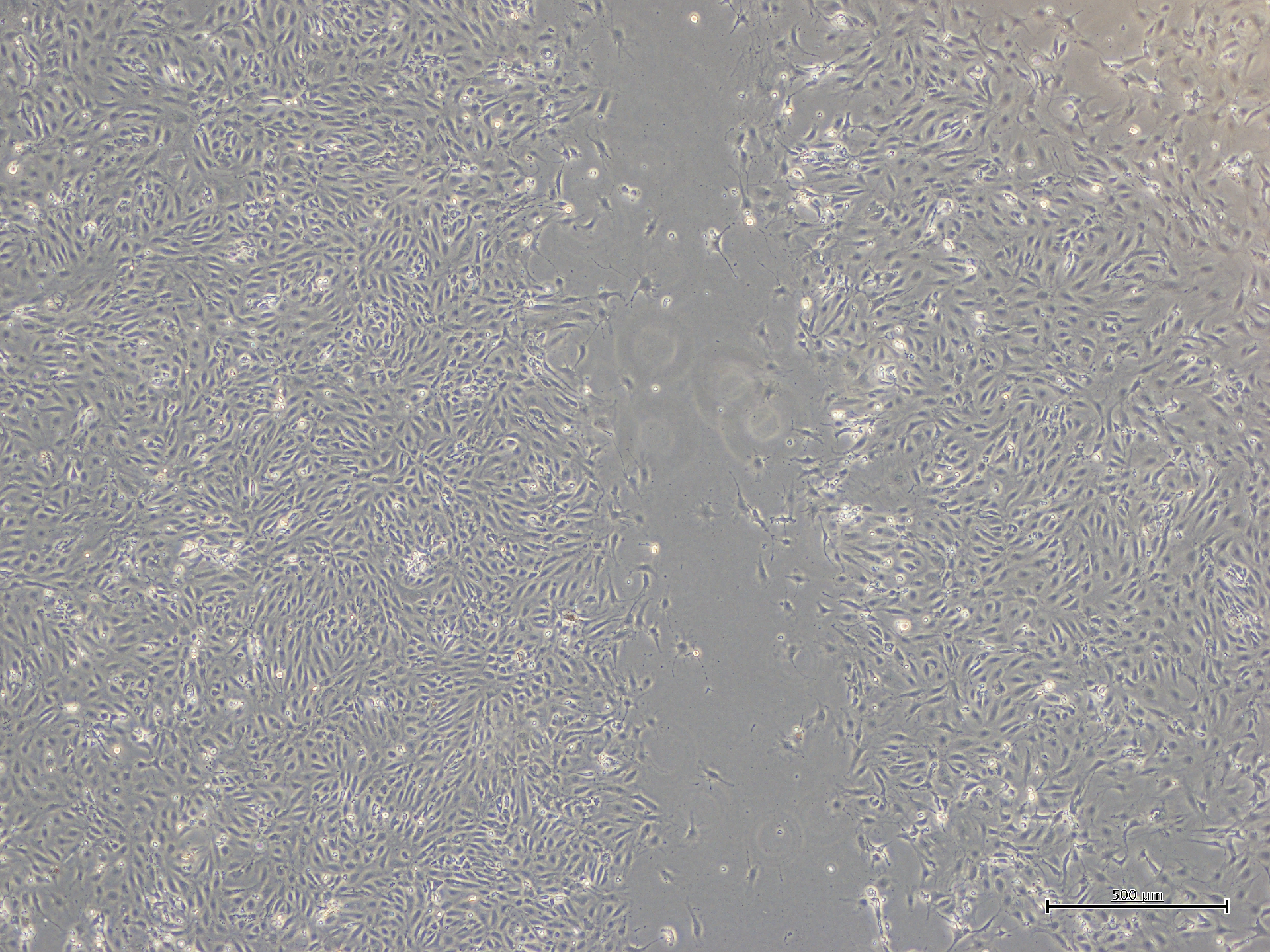

Supplement: Supplementary file 5 — Source data Fig. 3 [file 44318_2026_759_MOESM5_ESM.zip › SD Figure 3/3E/E11 30hr/Cdkal1 KO 2TIF.TIF]

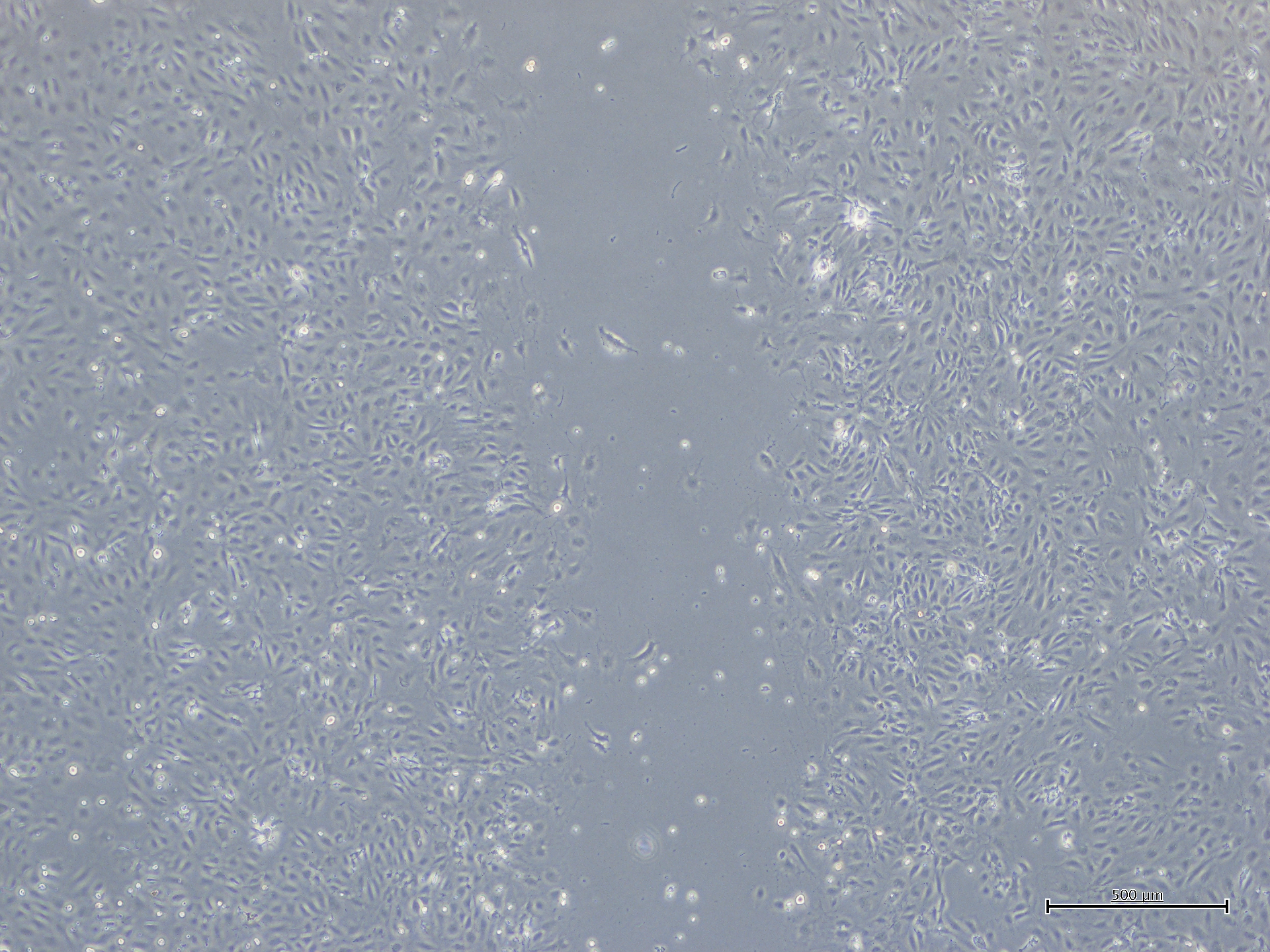

Supplement: Supplementary file 5 — Source data Fig. 3 [file 44318_2026_759_MOESM5_ESM.zip › SD Figure 3/3E/E11 30hr/Cdkal1 KO 1.TIF]

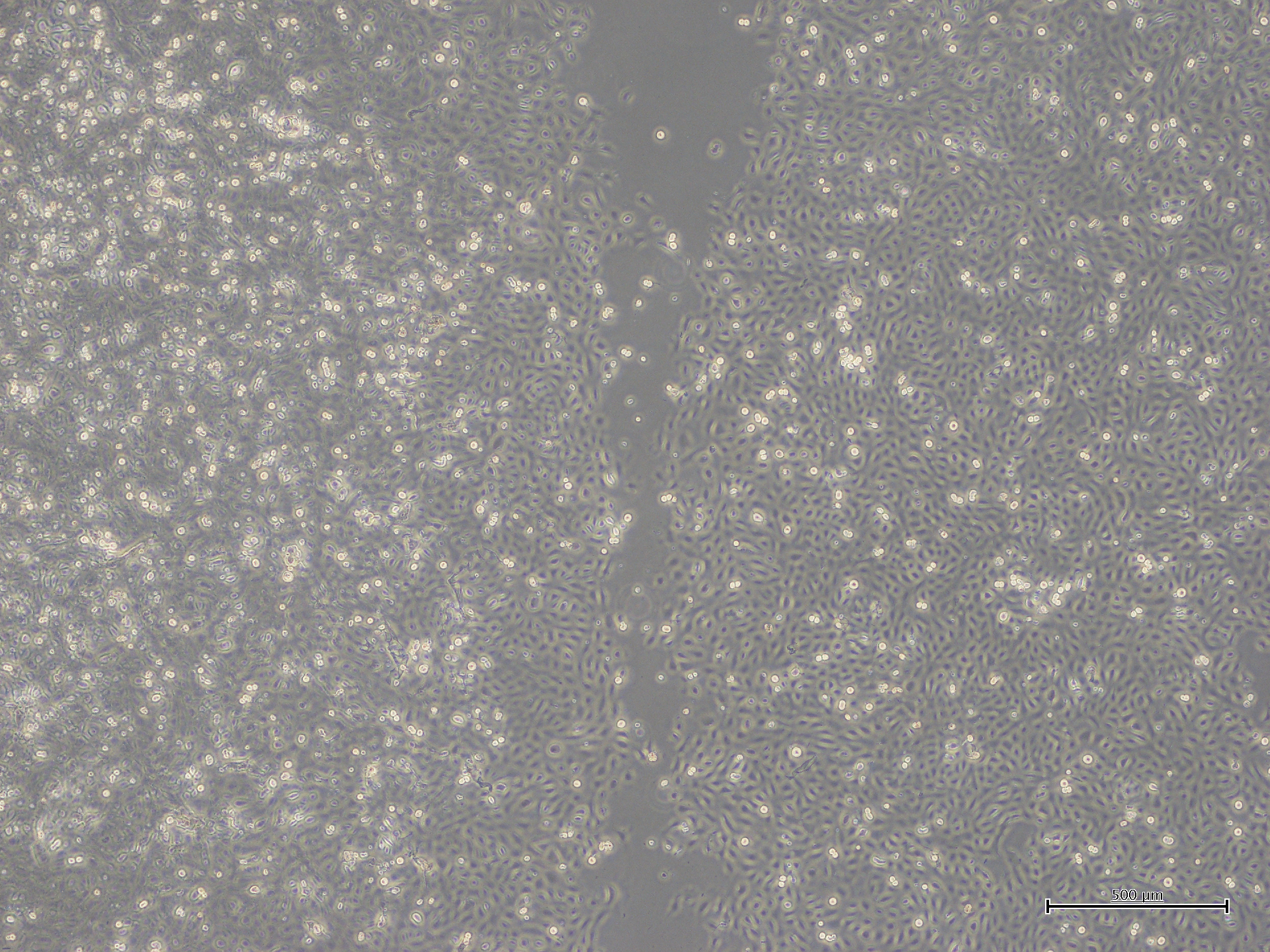

Supplement: Supplementary file 5 — Source data Fig. 3 [file 44318_2026_759_MOESM5_ESM.zip › SD Figure 3/3G/SVI 30hr/KO 1.TIF]

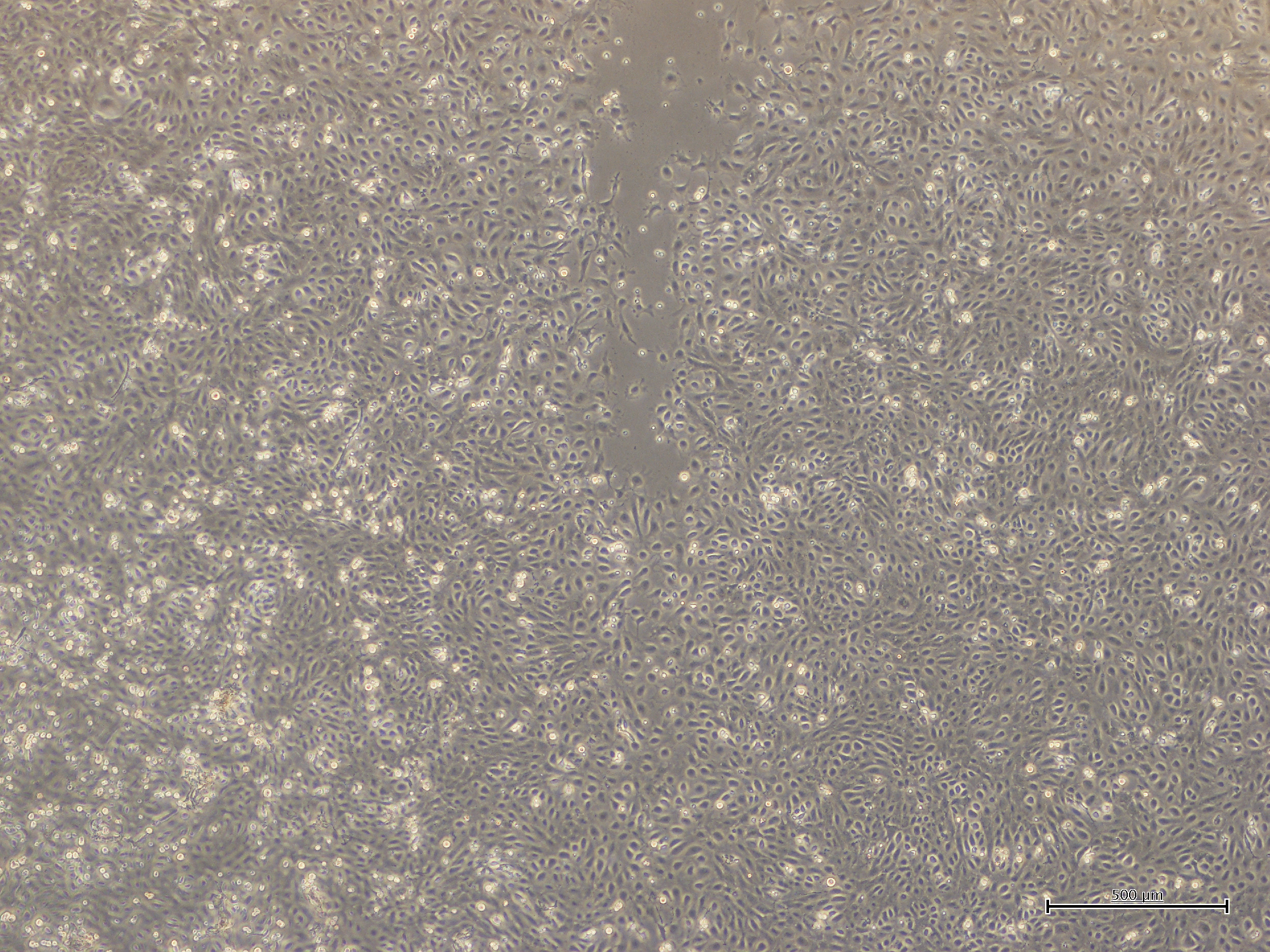

Supplement: Supplementary file 5 — Source data Fig. 3 [file 44318_2026_759_MOESM5_ESM.zip › SD Figure 3/3G/SVI 30hr/sgControl.TIF]

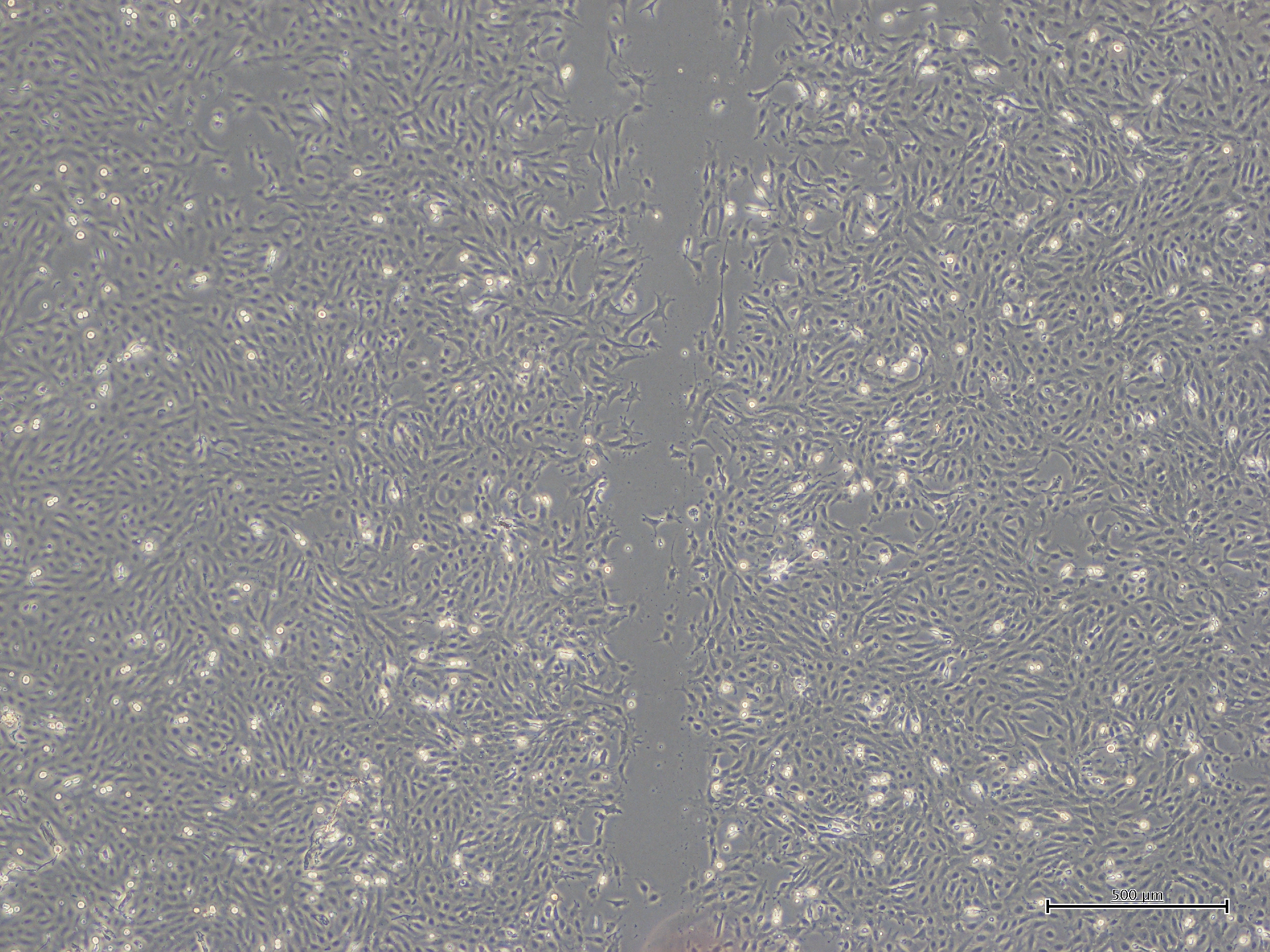

Supplement: Supplementary file 5 — Source data Fig. 3 [file 44318_2026_759_MOESM5_ESM.zip › SD Figure 3/3G/SVI 30hr/KO 2.TIF]

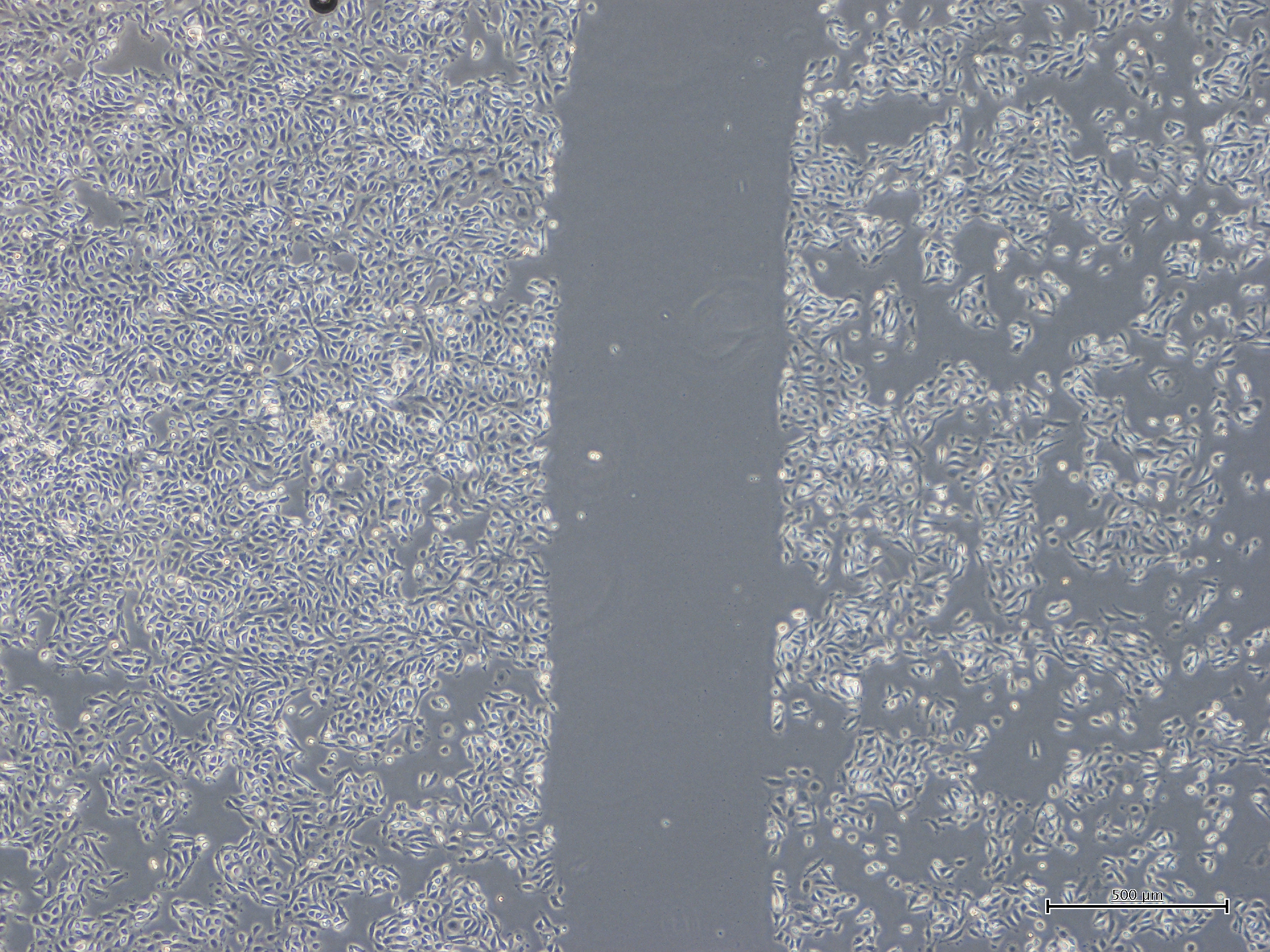

Supplement: Supplementary file 5 — Source data Fig. 3 [file 44318_2026_759_MOESM5_ESM.zip › SD Figure 3/3G/SVI 0hr/KO 1.TIF]

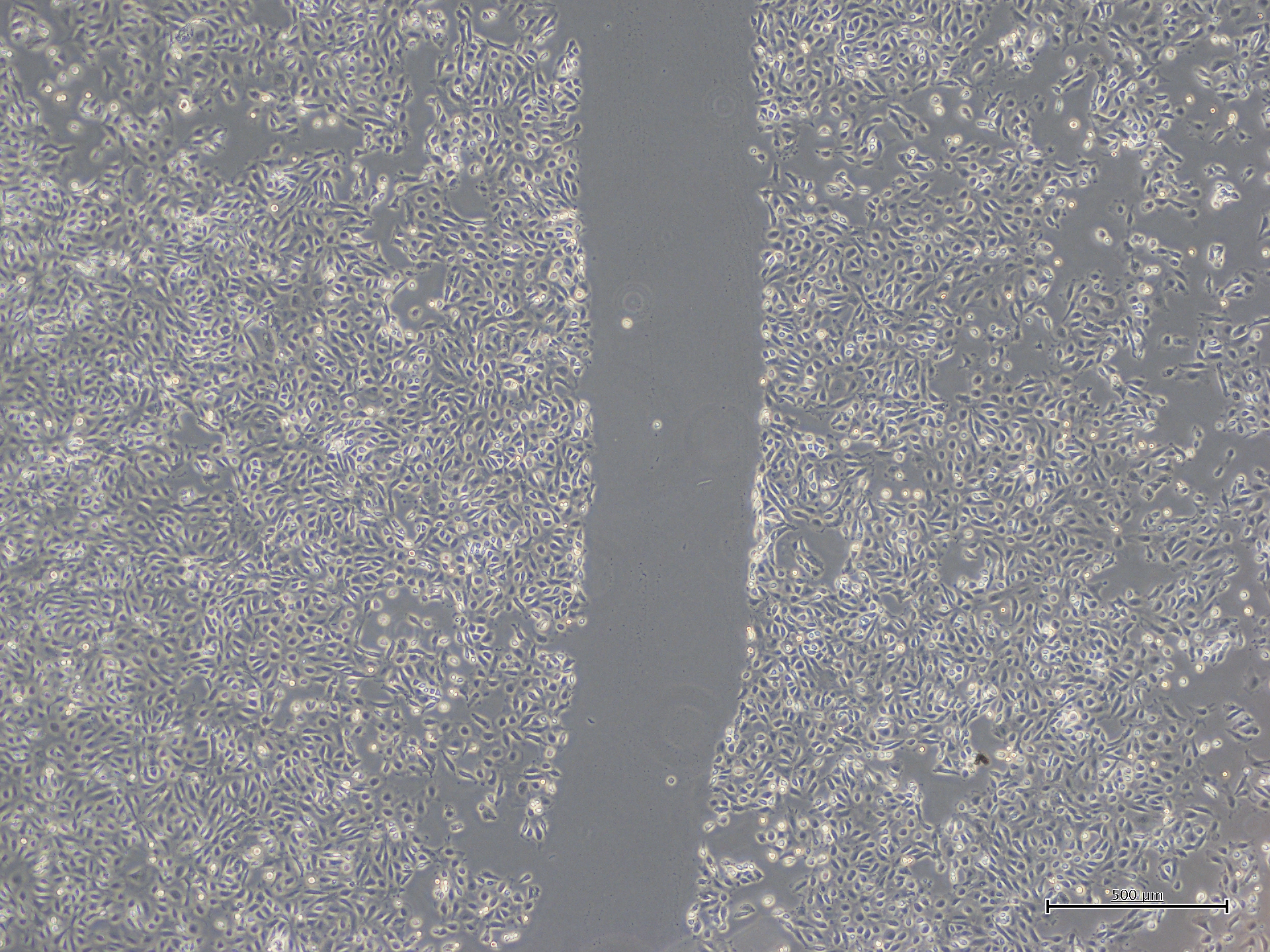

Supplement: Supplementary file 5 — Source data Fig. 3 [file 44318_2026_759_MOESM5_ESM.zip › SD Figure 3/3G/SVI 0hr/SgControl.TIF]

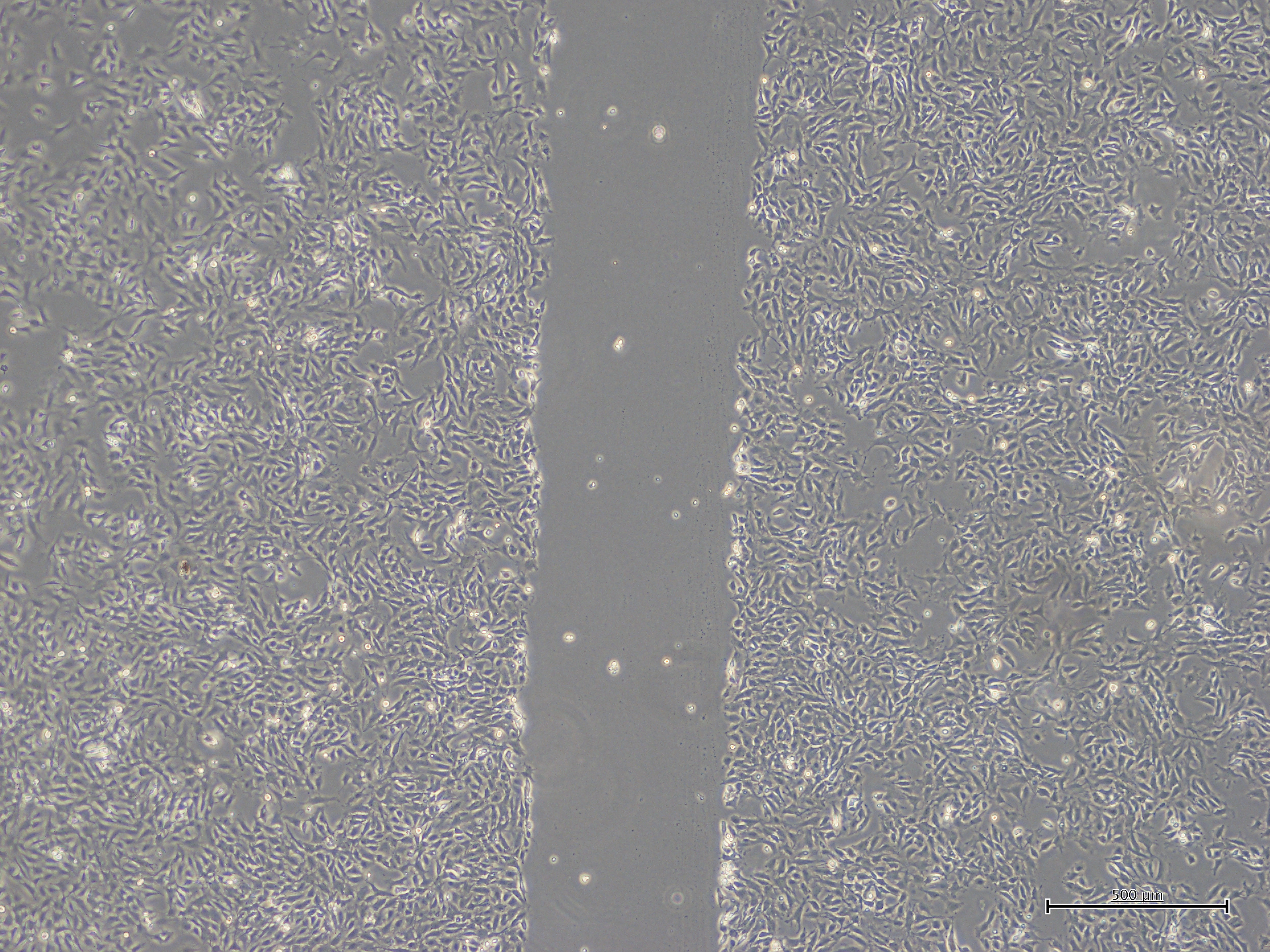

Supplement: Supplementary file 5 — Source data Fig. 3 [file 44318_2026_759_MOESM5_ESM.zip › SD Figure 3/3G/SVI 0hr/KO 2.TIF]

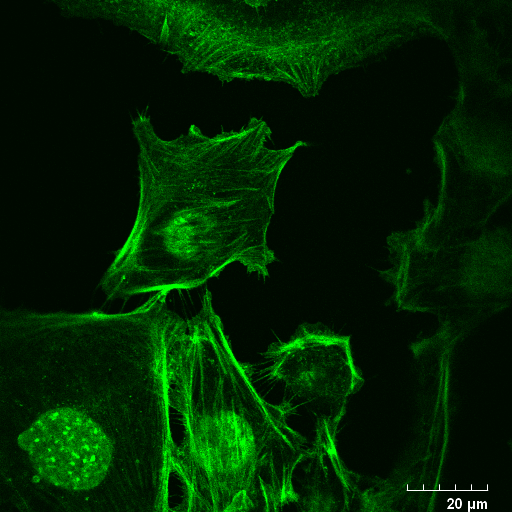

Supplement: Supplementary file 5 — Source data Fig. 3 [file 44318_2026_759_MOESM5_ESM.zip › SD Figure 3/3I/SVI/SVI sgControl.tif]

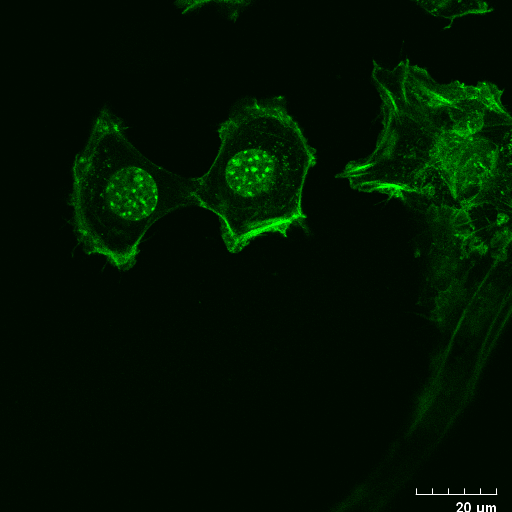

Supplement: Supplementary file 5 — Source data Fig. 3 [file 44318_2026_759_MOESM5_ESM.zip › SD Figure 3/3I/SVI/SVI KO 1.tif]

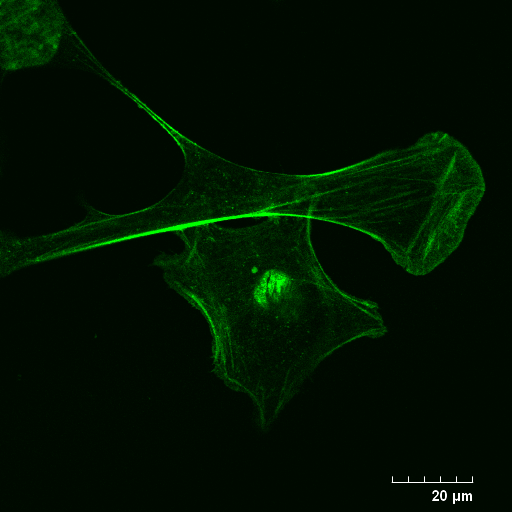

Supplement: Supplementary file 5 — Source data Fig. 3 [file 44318_2026_759_MOESM5_ESM.zip › SD Figure 3/3I/SVI/SVI KO 2.tif]

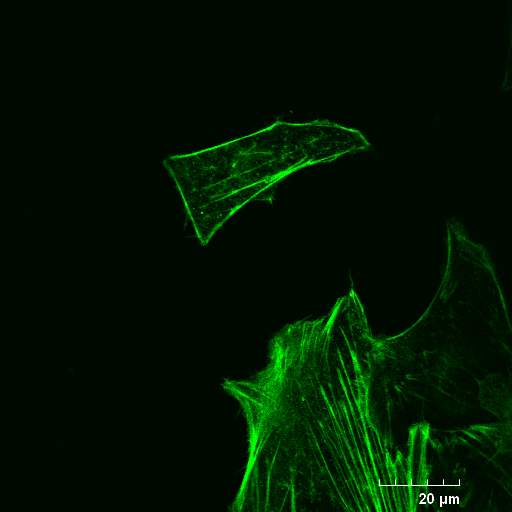

Supplement: Supplementary file 5 — Source data Fig. 3 [file 44318_2026_759_MOESM5_ESM.zip › SD Figure 3/3I/E11/E11 KO 1.tif]

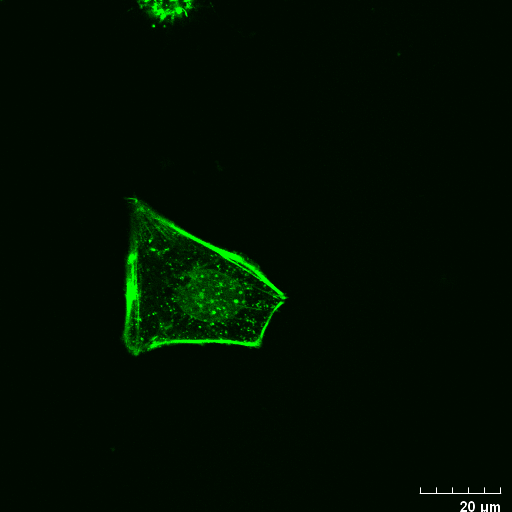

Supplement: Supplementary file 5 — Source data Fig. 3 [file 44318_2026_759_MOESM5_ESM.zip › SD Figure 3/3I/E11/E11 KO 2.tif]

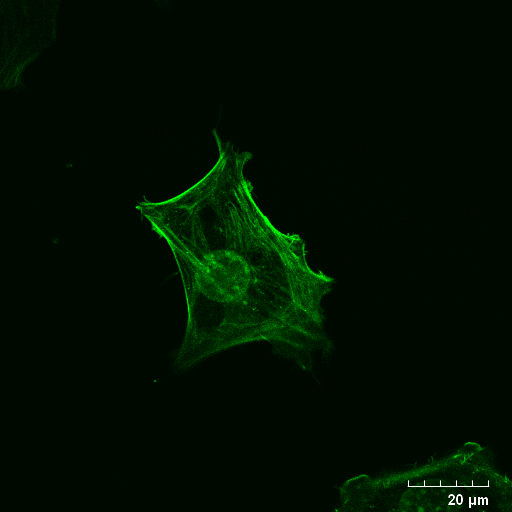

Supplement: Supplementary file 5 — Source data Fig. 3 [file 44318_2026_759_MOESM5_ESM.zip › SD Figure 3/3I/E11/E11 sgControl.tif]

Figure 4H

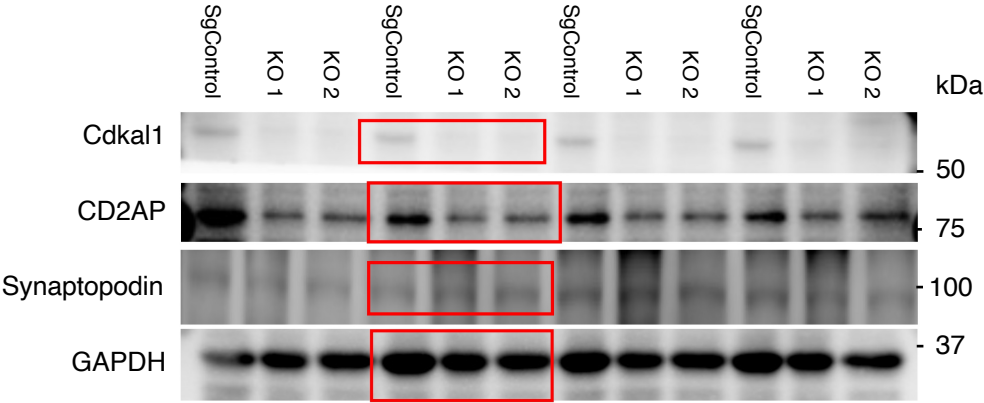

Figure 4K

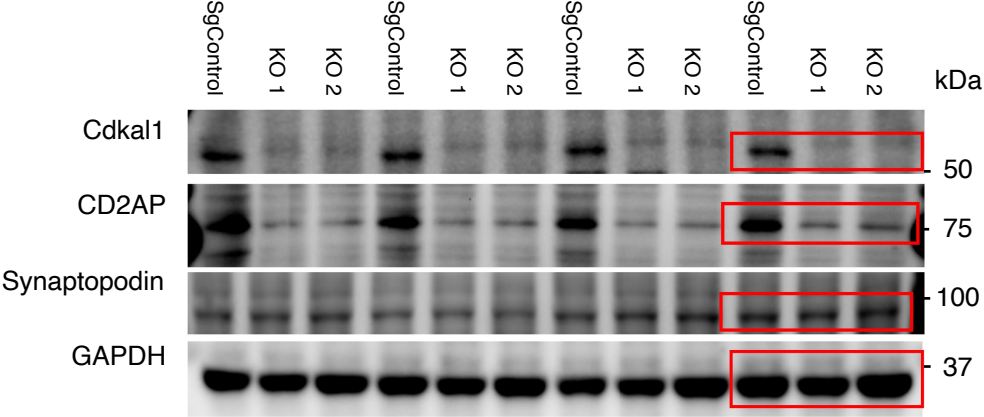

Figure 4M

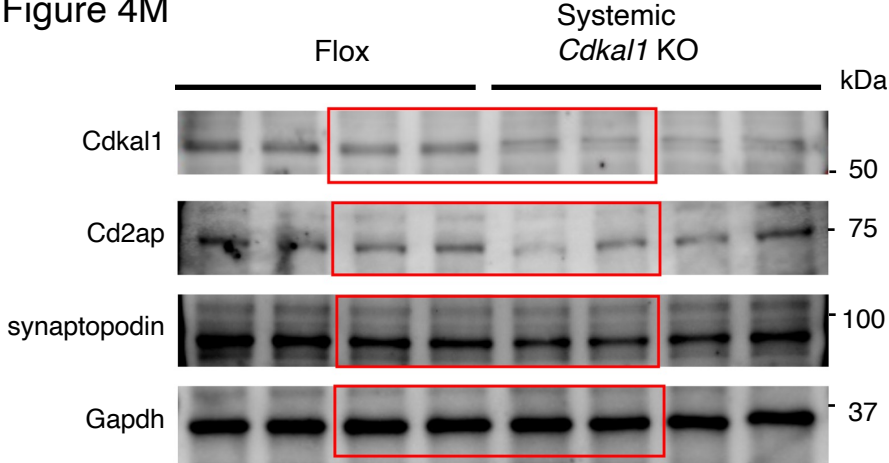

Supplement: Supplementary file 6 — Source data Fig. 4 [file 44318_2026_759_MOESM6_ESM.zip › SD Figure 4/4H,K and M.pdf]

Figure 5A

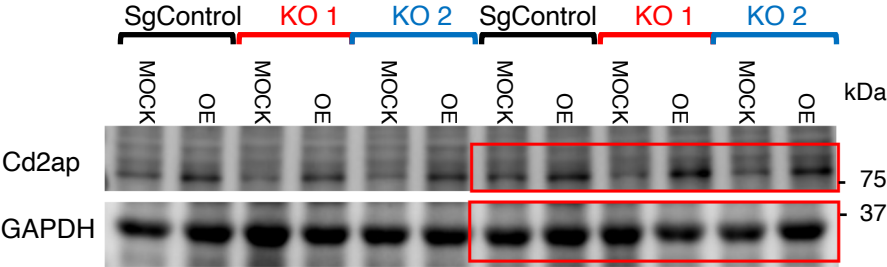

Supplement: Supplementary file 7 — Source data Fig. 5 [file 44318_2026_759_MOESM7_ESM.zip › SD FIgure 5/5A/5A.pdf]

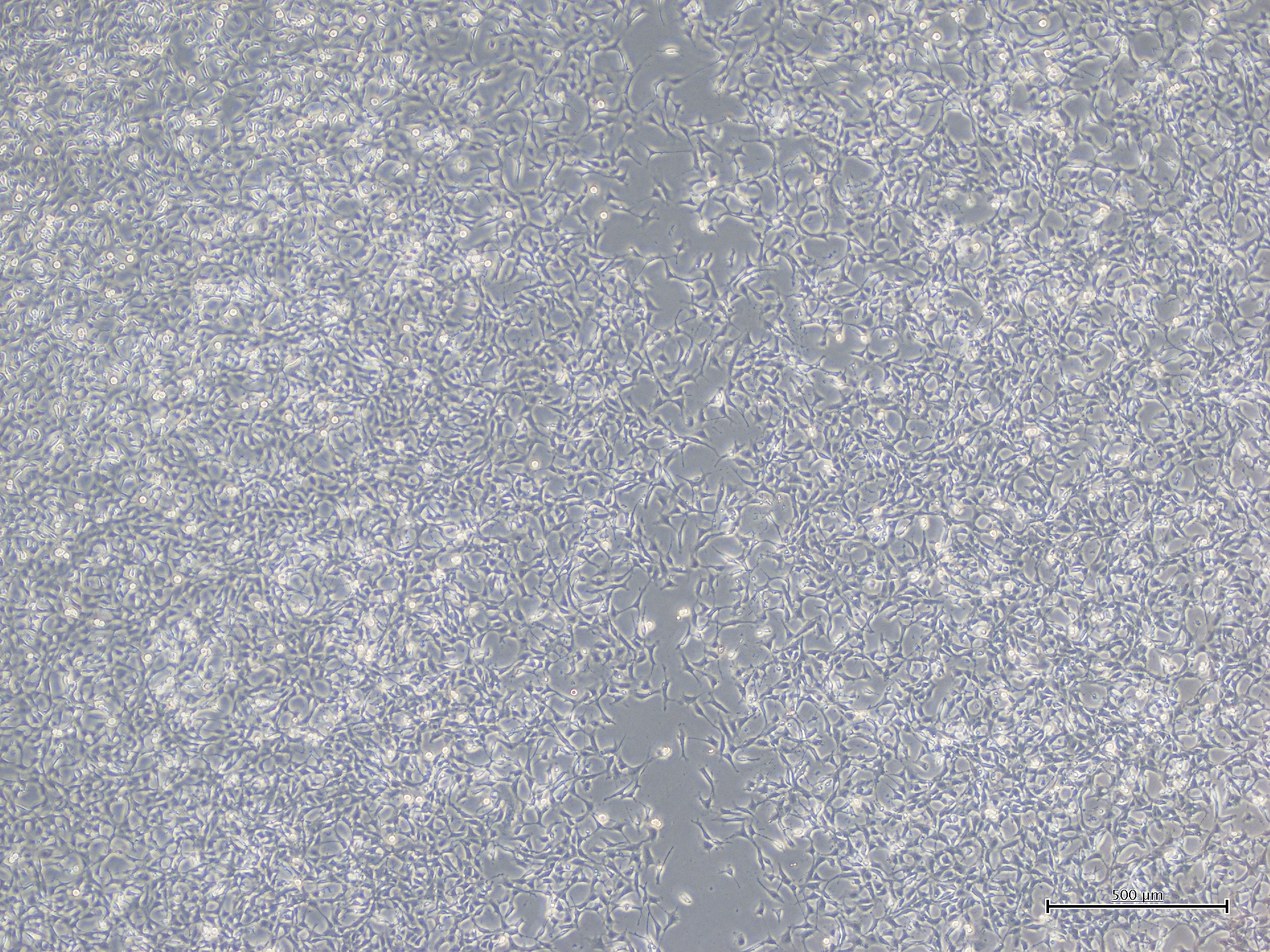

Supplement: Supplementary file 7 — Source data Fig. 5 [file 44318_2026_759_MOESM7_ESM.zip › SD FIgure 5/5F/Cd2ap 30hr/KO 1.TIF]

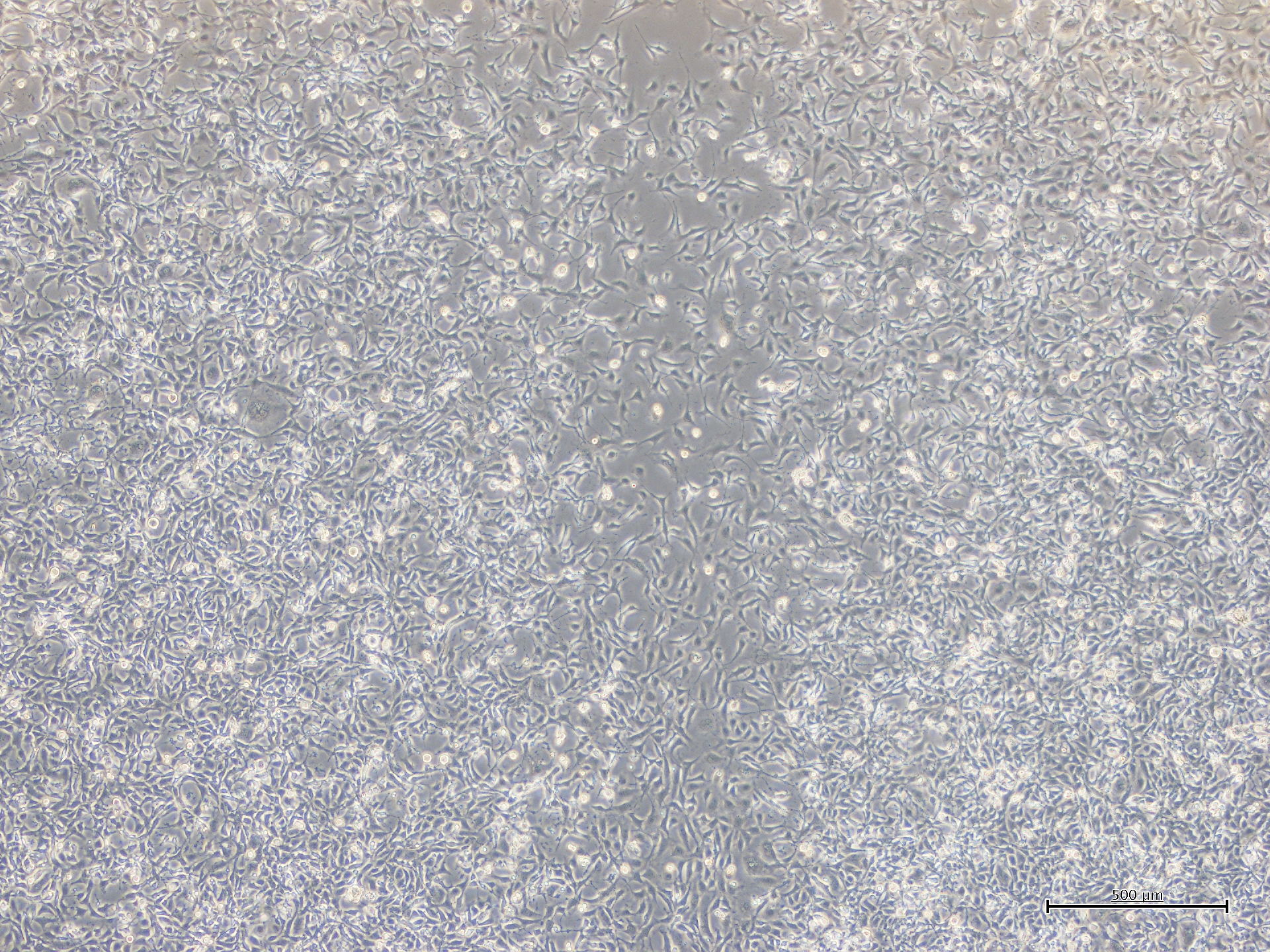

Supplement: Supplementary file 7 — Source data Fig. 5 [file 44318_2026_759_MOESM7_ESM.zip › SD FIgure 5/5F/Cd2ap 30hr/sgControl.TIF]

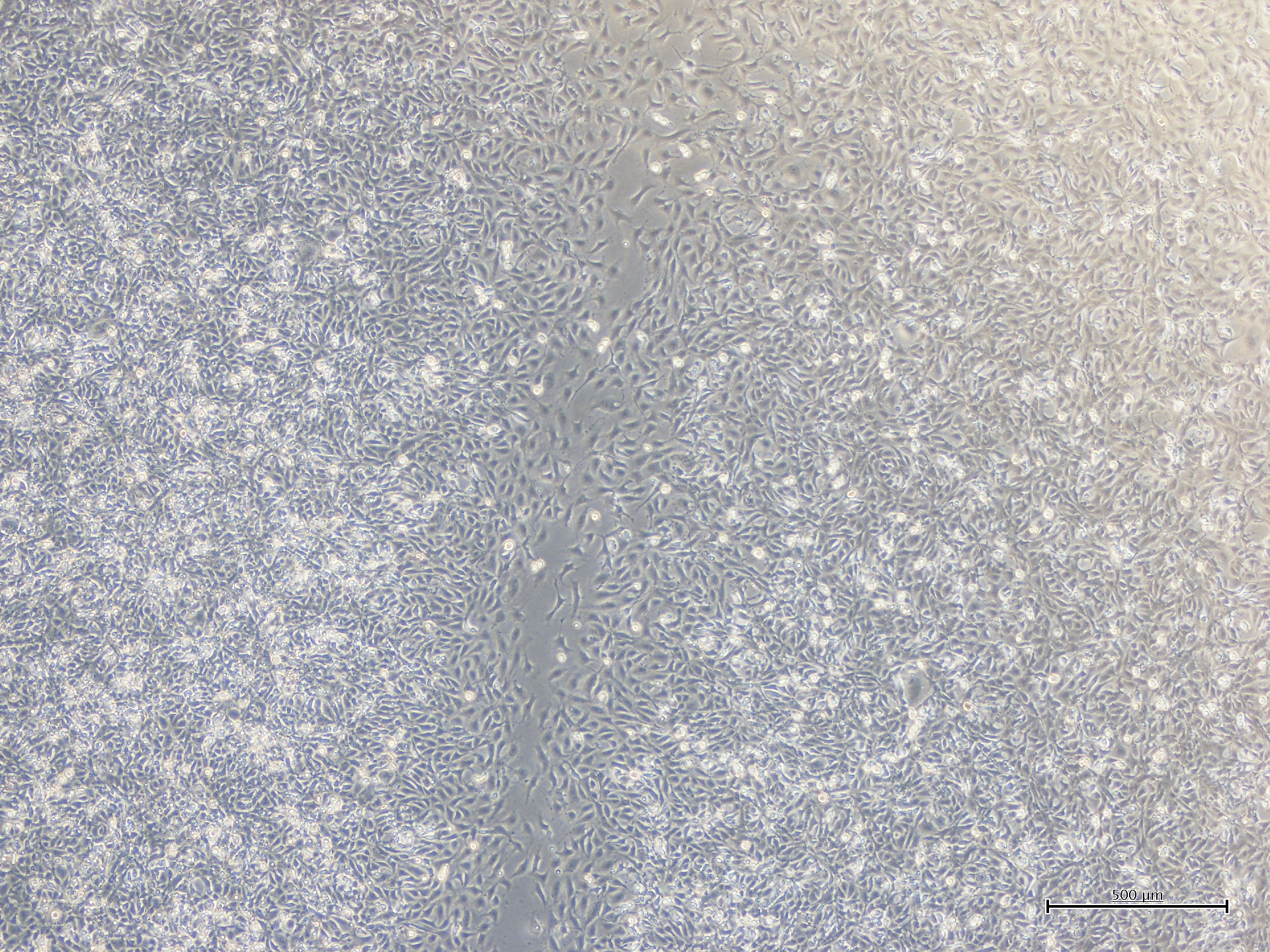

Supplement: Supplementary file 7 — Source data Fig. 5 [file 44318_2026_759_MOESM7_ESM.zip › SD FIgure 5/5F/Cd2ap 30hr/KO 2.TIF]

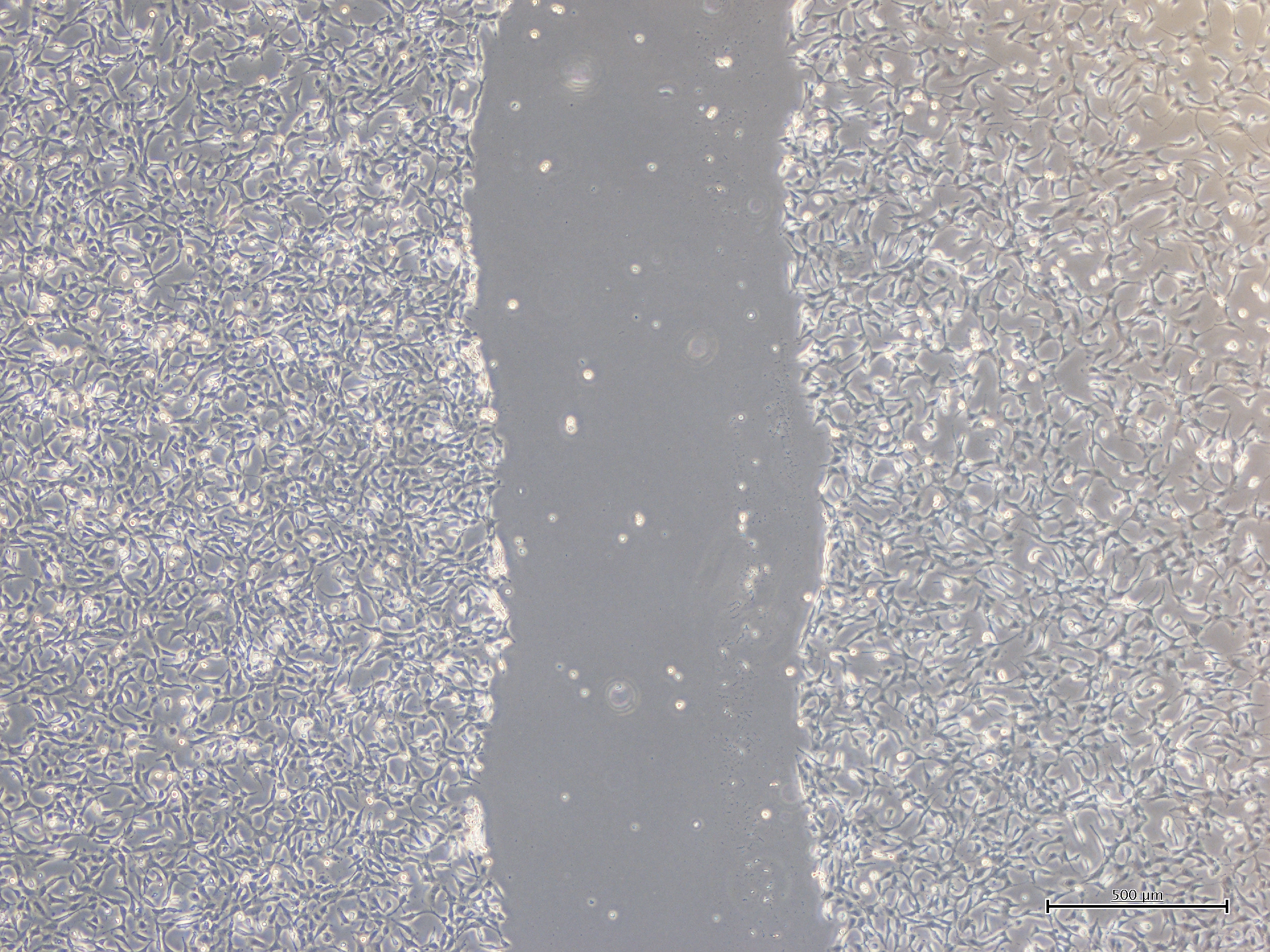

Supplement: Supplementary file 7 — Source data Fig. 5 [file 44318_2026_759_MOESM7_ESM.zip › SD FIgure 5/5F/Cd2ap 0hr/KO 1.TIF]

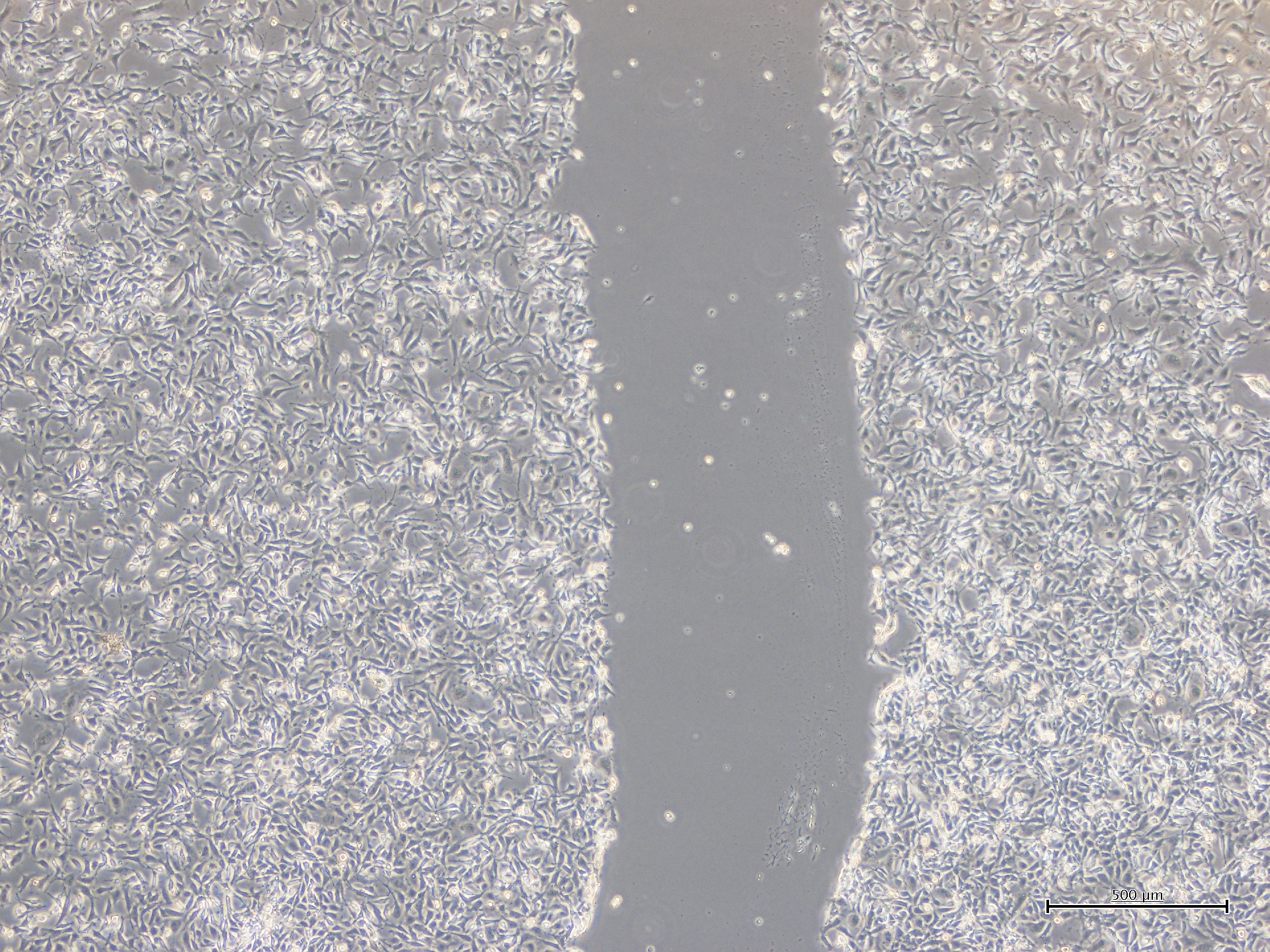

Supplement: Supplementary file 7 — Source data Fig. 5 [file 44318_2026_759_MOESM7_ESM.zip › SD FIgure 5/5F/Cd2ap 0hr/sgControl.TIF]

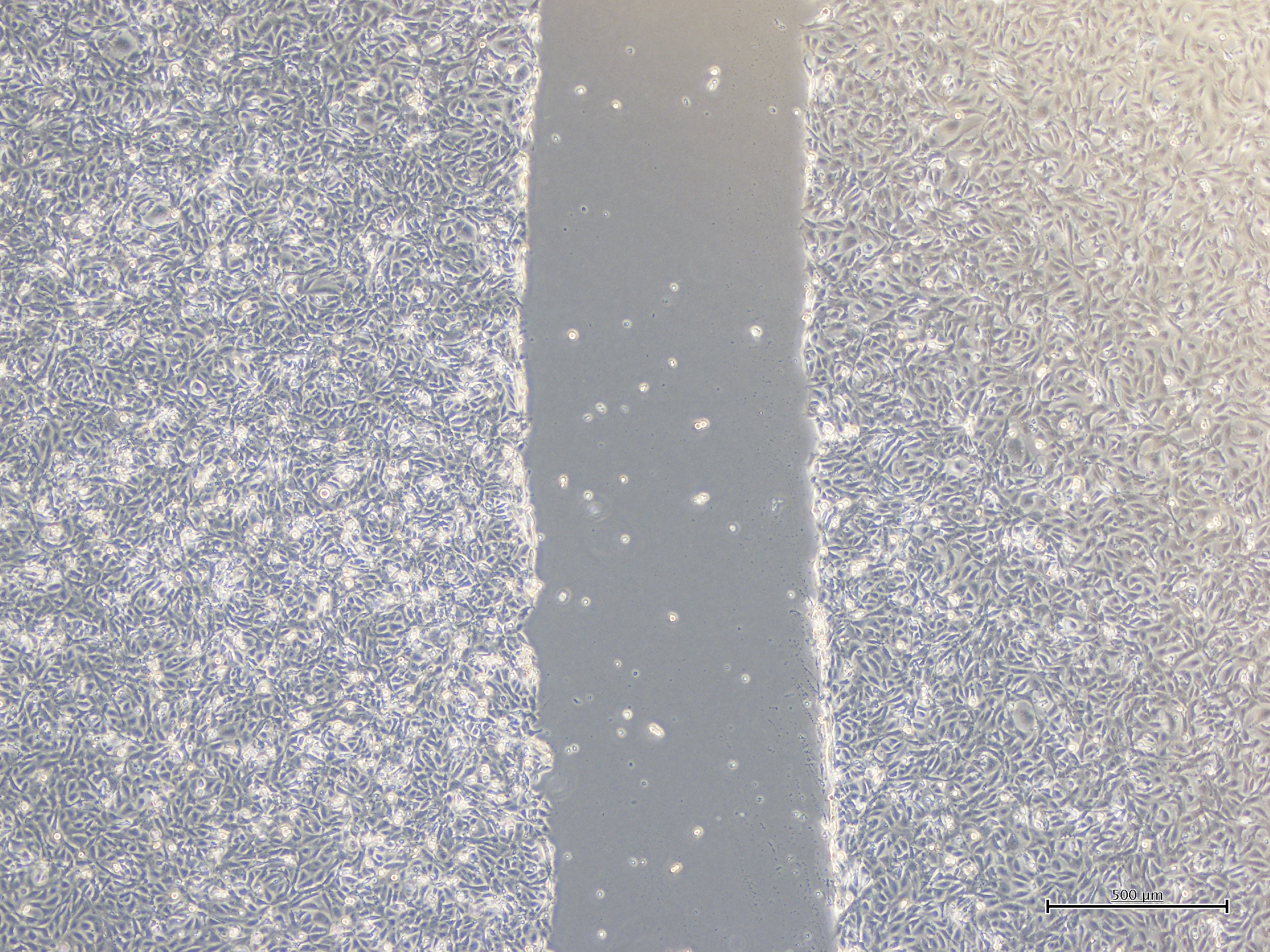

Supplement: Supplementary file 7 — Source data Fig. 5 [file 44318_2026_759_MOESM7_ESM.zip › SD FIgure 5/5F/Cd2ap 0hr/KO 2.TIF]

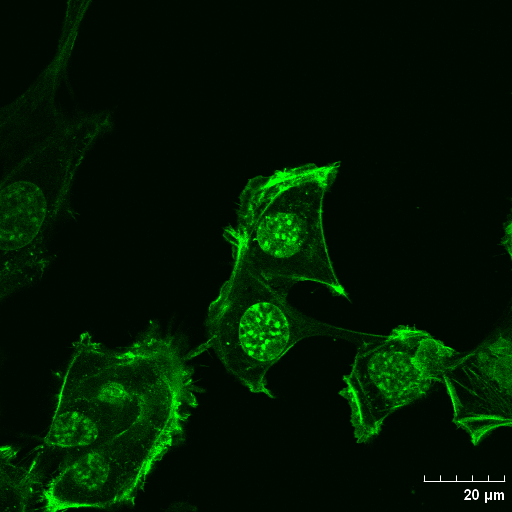

Supplement: Supplementary file 7 — Source data Fig. 5 [file 44318_2026_759_MOESM7_ESM.zip › SD FIgure 5/5C/Mock/KO 1.tif]

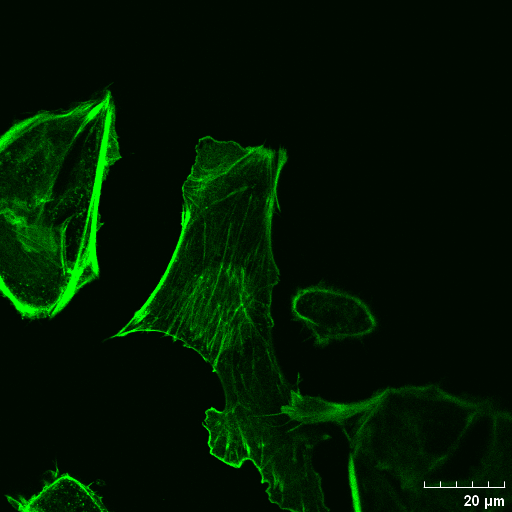

Supplement: Supplementary file 7 — Source data Fig. 5 [file 44318_2026_759_MOESM7_ESM.zip › SD FIgure 5/5C/Mock/sgControl.tif]

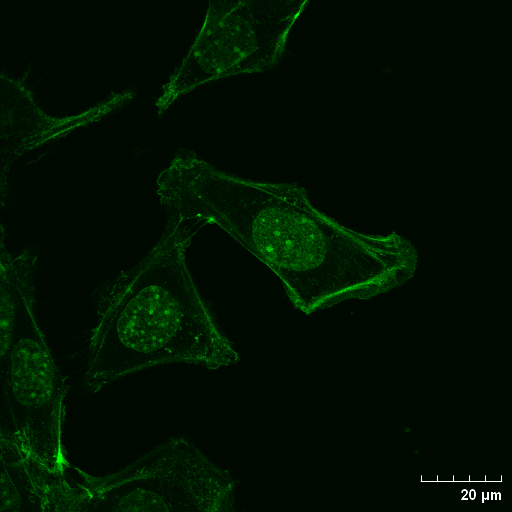

Supplement: Supplementary file 7 — Source data Fig. 5 [file 44318_2026_759_MOESM7_ESM.zip › SD FIgure 5/5C/Mock/KO 2.tif]

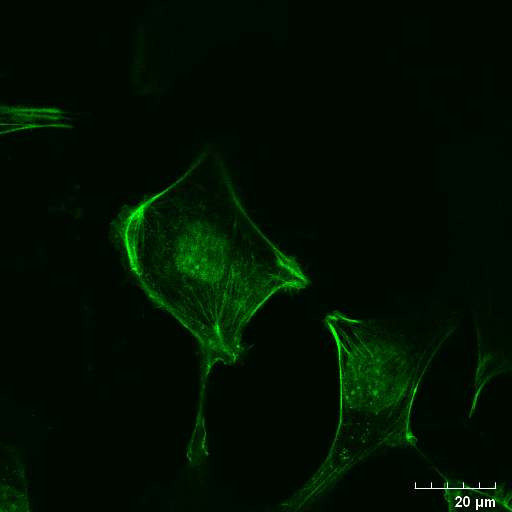

Supplement: Supplementary file 7 — Source data Fig. 5 [file 44318_2026_759_MOESM7_ESM.zip › SD FIgure 5/5C/Cd2ap OE/sgComtrol.tif]

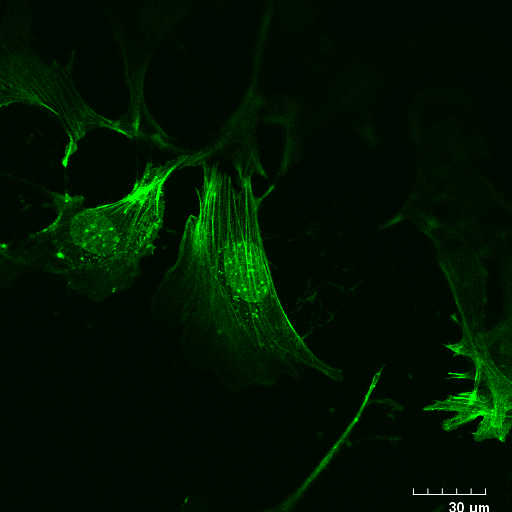

Supplement: Supplementary file 7 — Source data Fig. 5 [file 44318_2026_759_MOESM7_ESM.zip › SD FIgure 5/5C/Cd2ap OE/KO 1.tif]

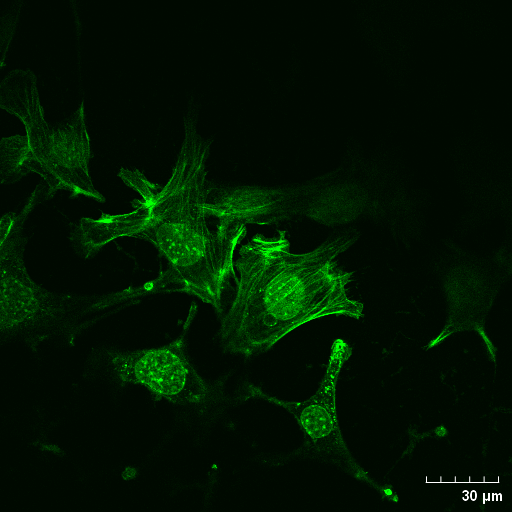

Supplement: Supplementary file 7 — Source data Fig. 5 [file 44318_2026_759_MOESM7_ESM.zip › SD FIgure 5/5C/Cd2ap OE/KO 2.tif]

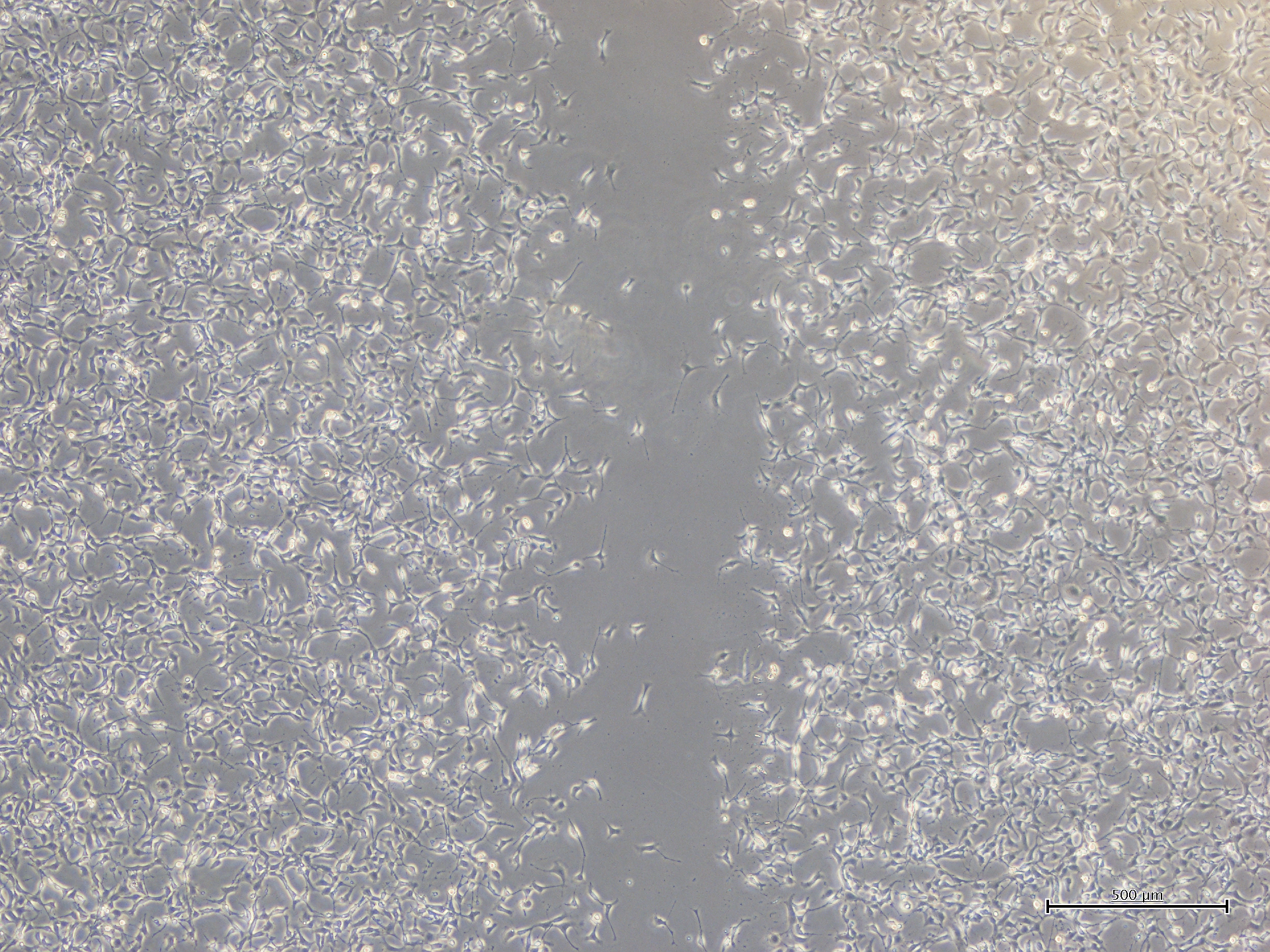

Supplement: Supplementary file 7 — Source data Fig. 5 [file 44318_2026_759_MOESM7_ESM.zip › SD FIgure 5/5E/Mock 30hr/KO 2TIF.TIF]

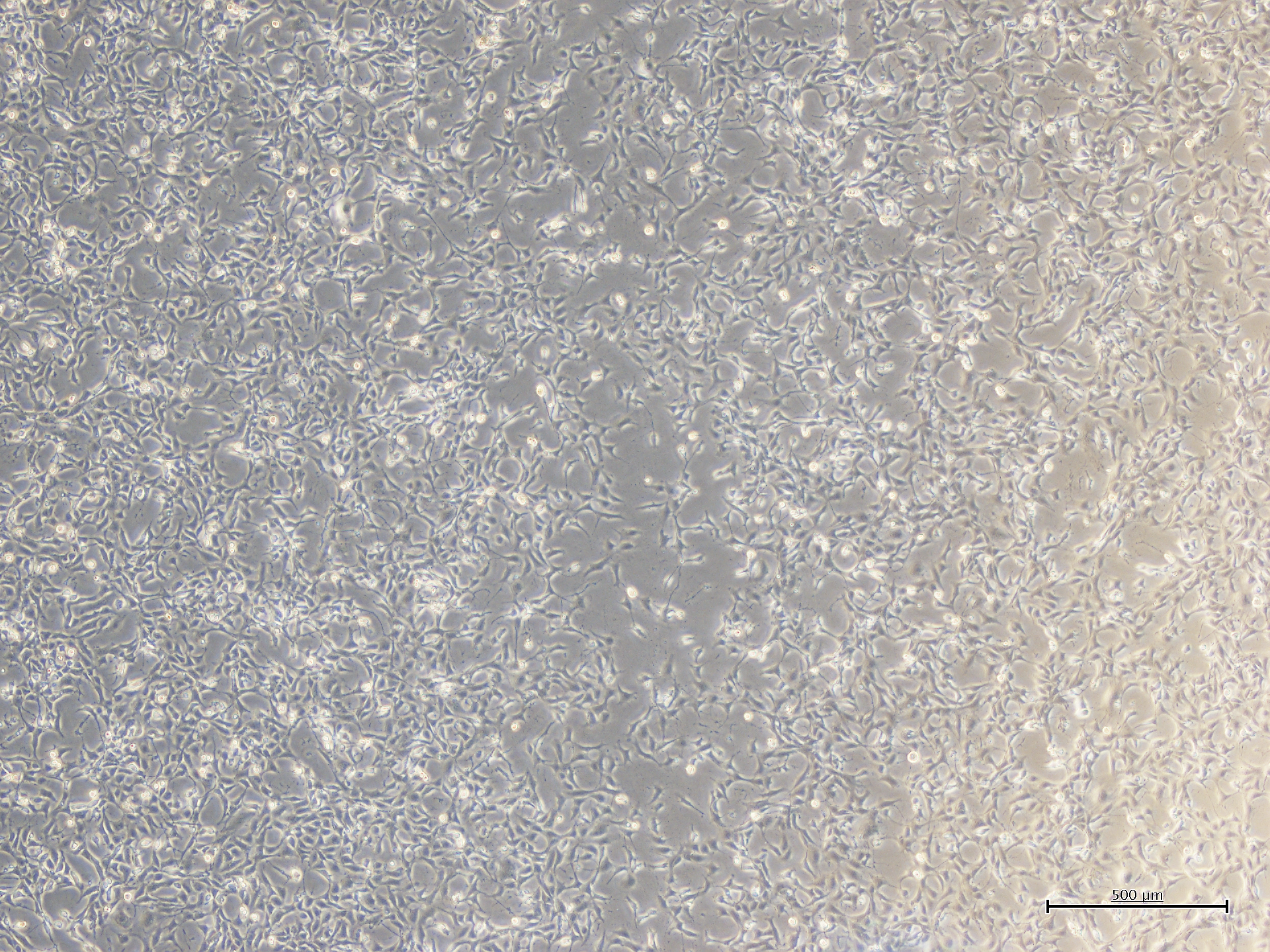

Supplement: Supplementary file 7 — Source data Fig. 5 [file 44318_2026_759_MOESM7_ESM.zip › SD FIgure 5/5E/Mock 30hr/sgControlTIF.TIF]

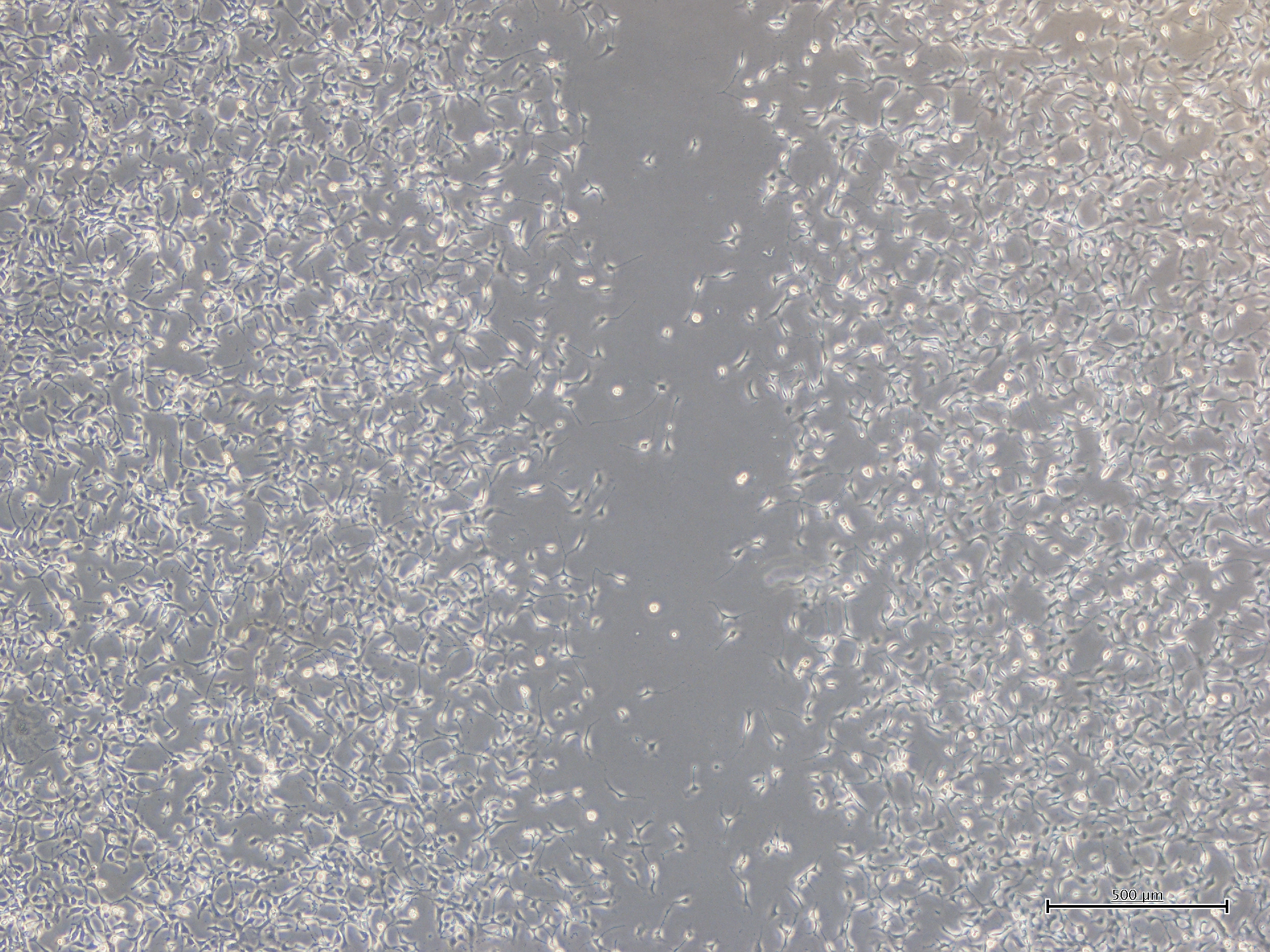

Supplement: Supplementary file 7 — Source data Fig. 5 [file 44318_2026_759_MOESM7_ESM.zip › SD FIgure 5/5E/Mock 30hr/KO 1.TIF]

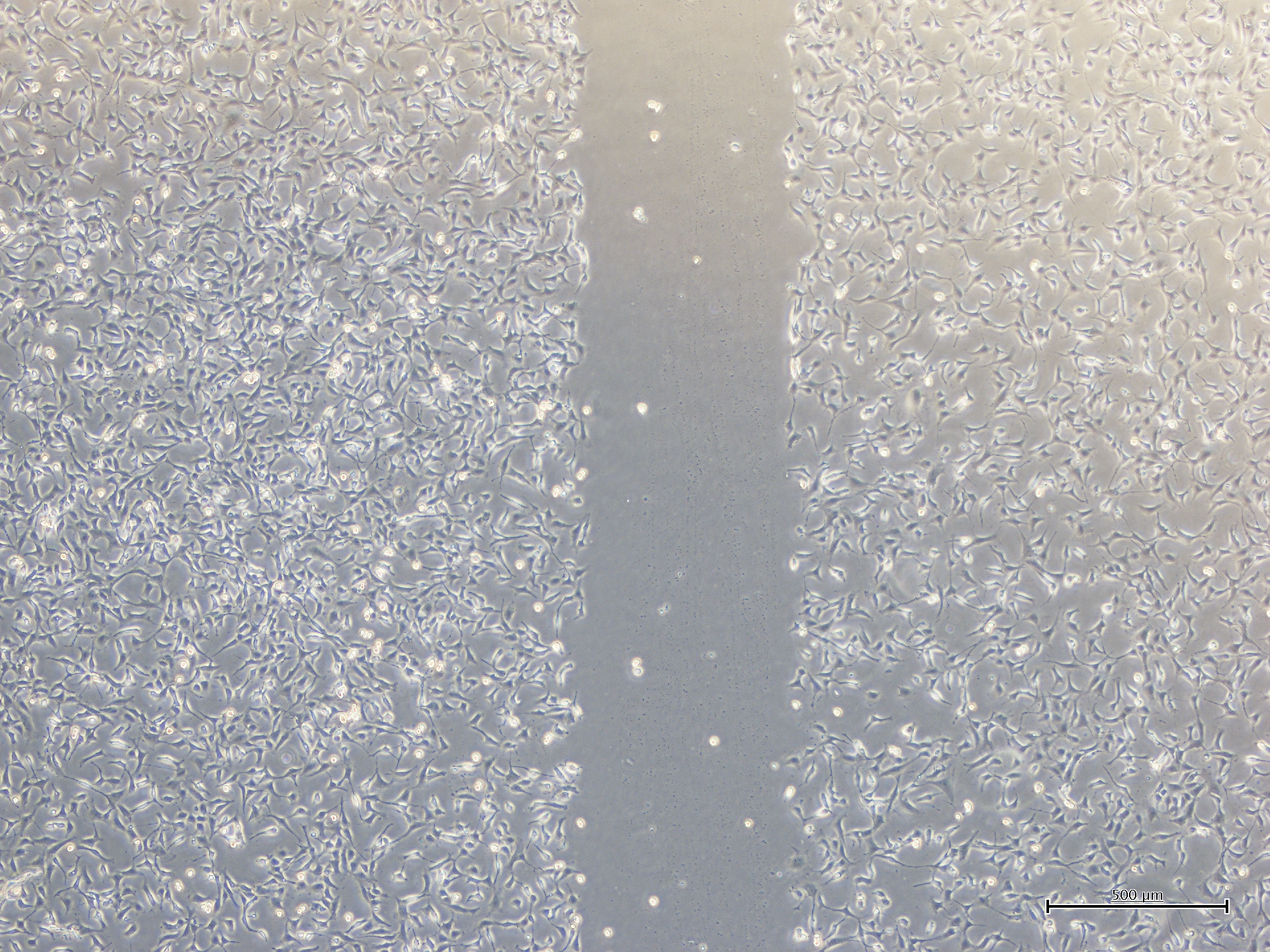

Supplement: Supplementary file 7 — Source data Fig. 5 [file 44318_2026_759_MOESM7_ESM.zip › SD FIgure 5/5E/Mock 0hr/KO 1.TIF]

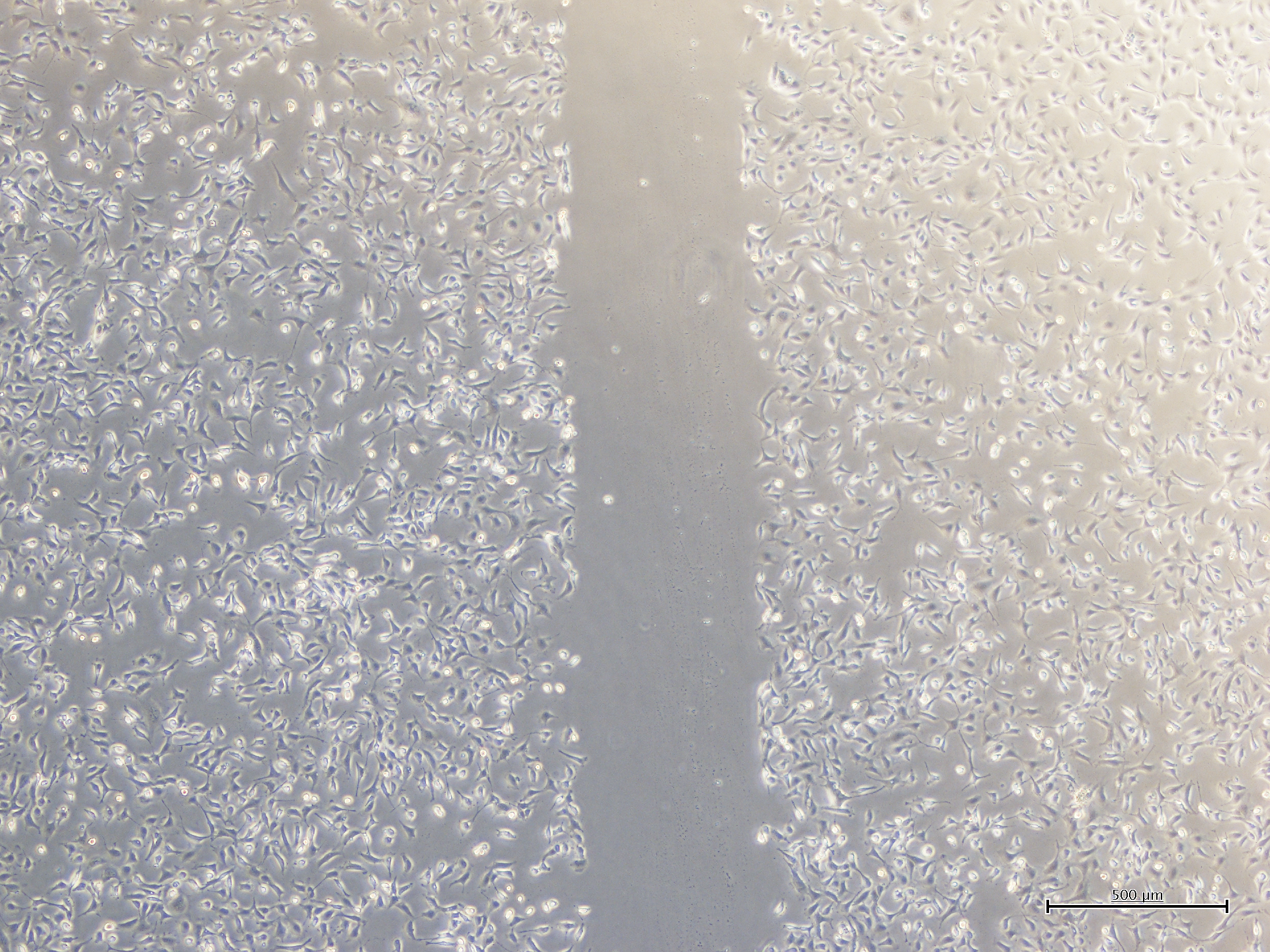

Supplement: Supplementary file 7 — Source data Fig. 5 [file 44318_2026_759_MOESM7_ESM.zip › SD FIgure 5/5E/Mock 0hr/sgControl.TIF]

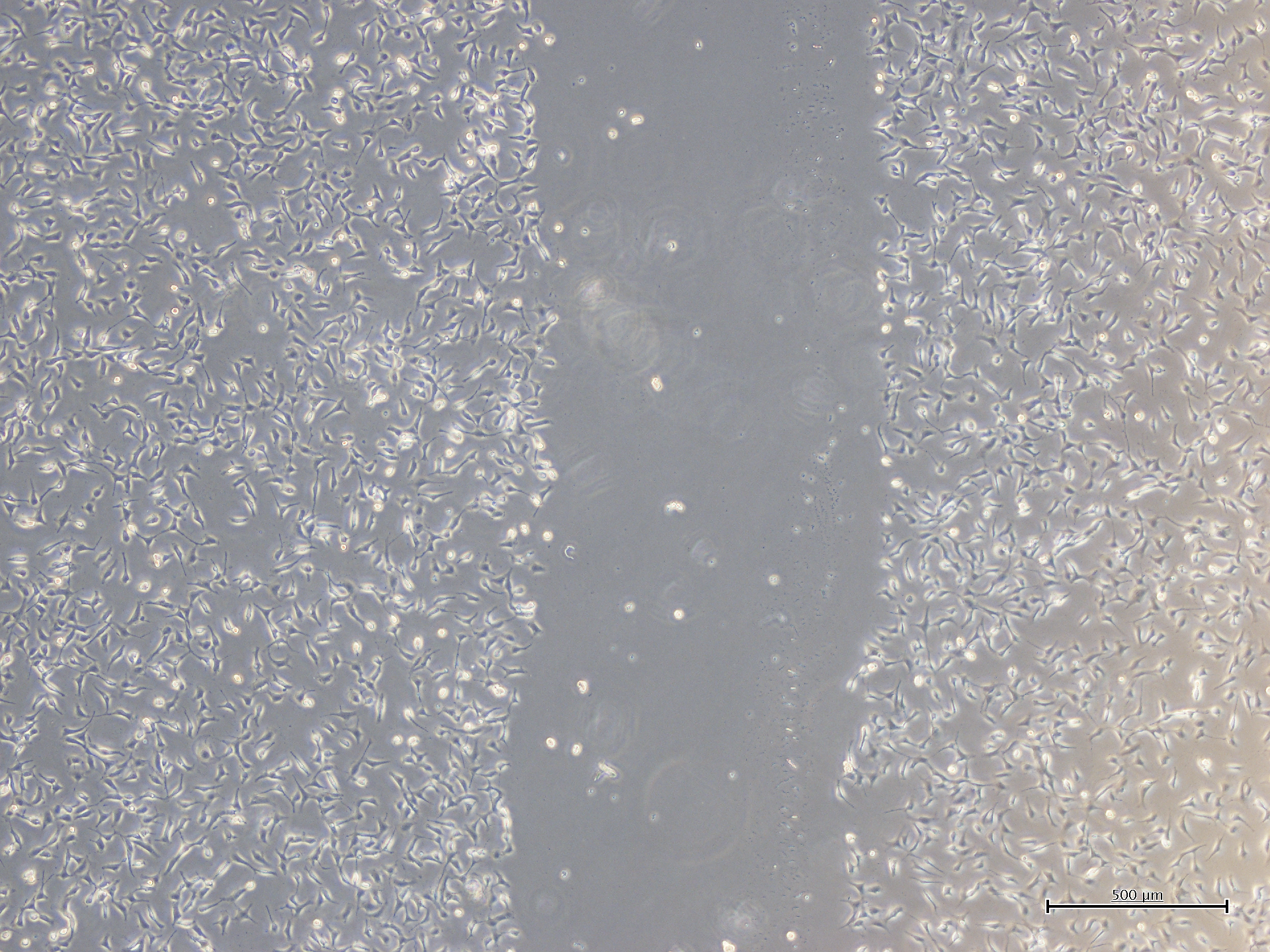

Supplement: Supplementary file 7 — Source data Fig. 5 [file 44318_2026_759_MOESM7_ESM.zip › SD FIgure 5/5E/Mock 0hr/KO 2.TIF]

Appendix Figure S6D

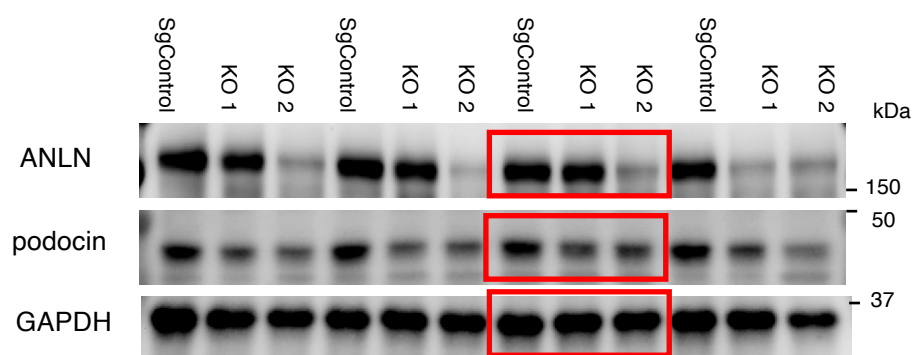

Supplement: Supplementary file 8 — EV and Appendix Figures Source Data [file 44318_2026_759_MOESM8_ESM.zip › SD EV Figureπü«πé│πâÆπéÜπâ╝/Appendix Figure S6/Appendix Figure S6D.pdf]

Appendix Figure S10.

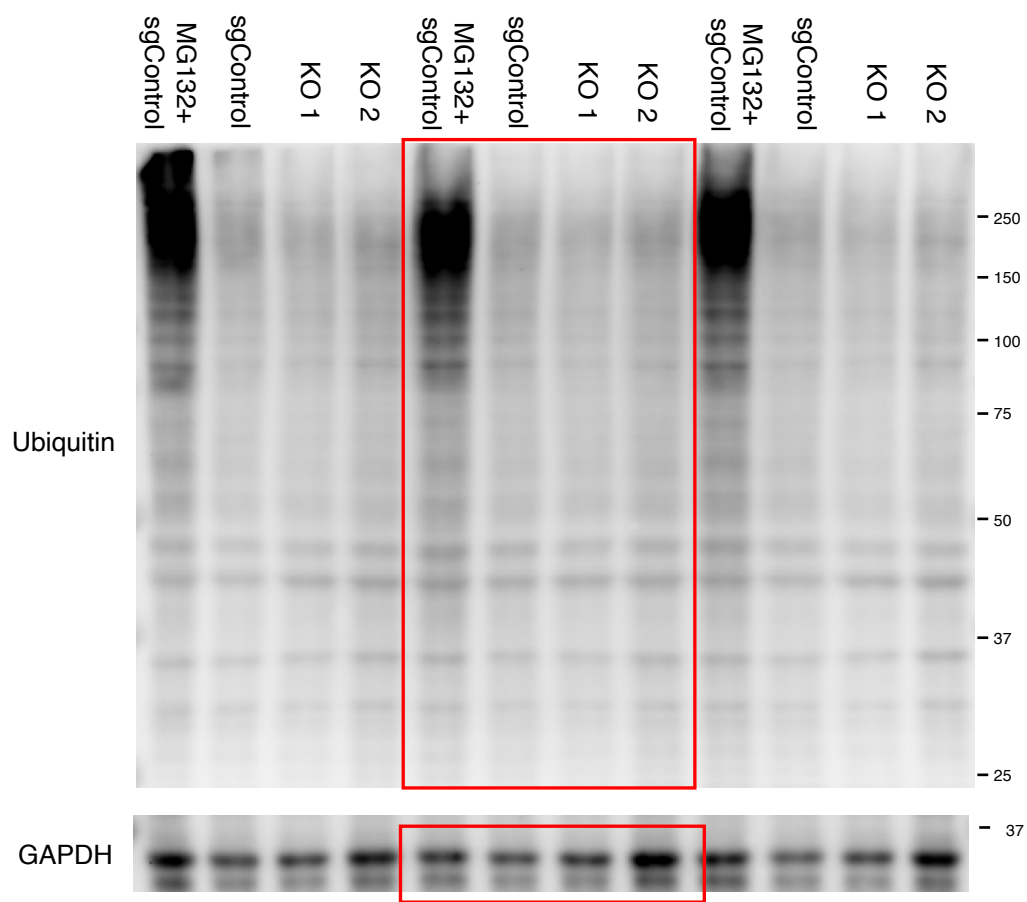

Supplement: Supplementary file 8 — EV and Appendix Figures Source Data [file 44318_2026_759_MOESM8_ESM.zip › SD EV Figureπü«πé│πâÆπéÜπâ╝/Appendix FIgure S10/Unedit immunoblot of Appendix Figure S10.pdf]

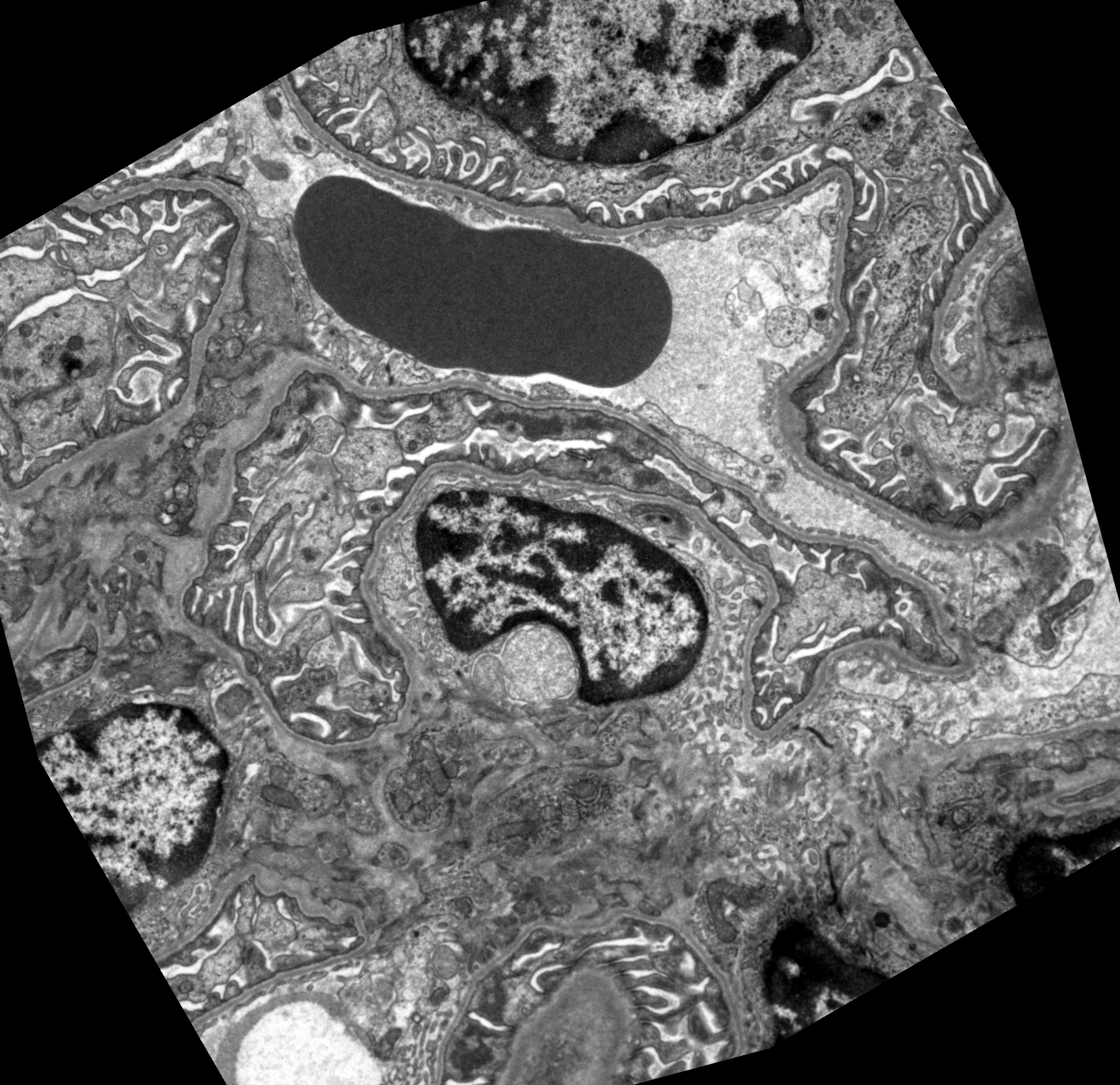

Supplement: Supplementary file 8 — EV and Appendix Figures Source Data [file 44318_2026_759_MOESM8_ESM.zip › SD EV Figureπü«πé│πâÆπéÜπâ╝/Appendix Figure S1/Appendix Figure S1A/Flox.tif]

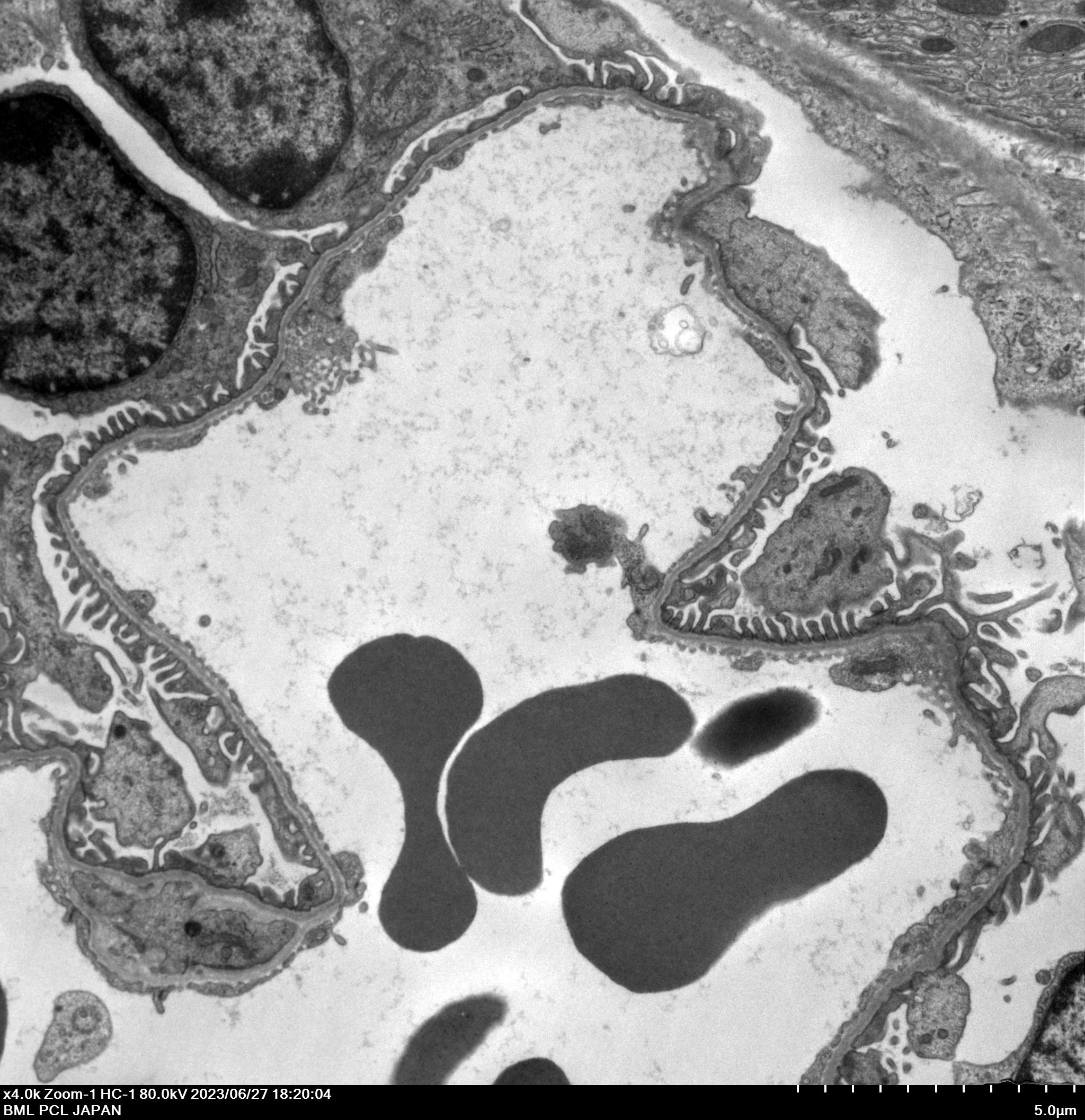

Supplement: Supplementary file 8 — EV and Appendix Figures Source Data [file 44318_2026_759_MOESM8_ESM.zip › SD EV Figureπü«πé│πâÆπéÜπâ╝/Appendix Figure S1/Appendix Figure S1A/Systemic Cdkal1 KO.tif]

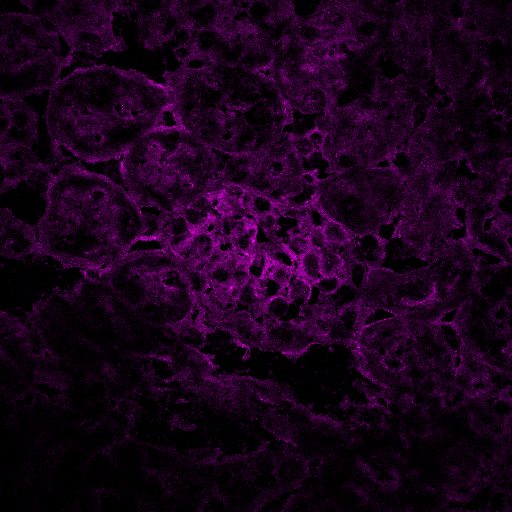

Supplement: Supplementary file 8 — EV and Appendix Figures Source Data [file 44318_2026_759_MOESM8_ESM.zip › SD EV Figureπü«πé│πâÆπéÜπâ╝/Appendix Figure S2/Flox Nephrin-CDKAL1/anti-CDKAL1.tif]

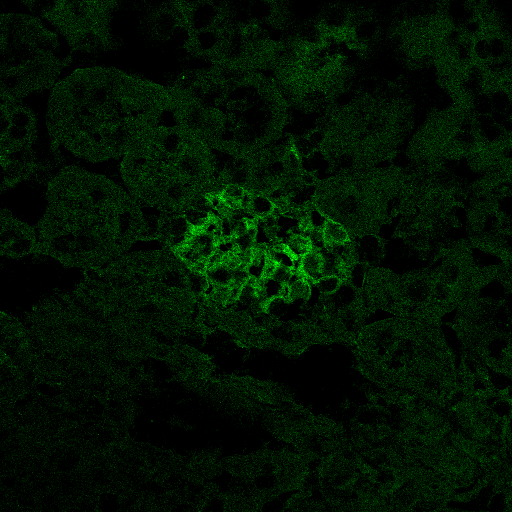

Supplement: Supplementary file 8 — EV and Appendix Figures Source Data [file 44318_2026_759_MOESM8_ESM.zip › SD EV Figureπü«πé│πâÆπéÜπâ╝/Appendix Figure S2/Flox Nephrin-CDKAL1/anti-Nephrin.tif]

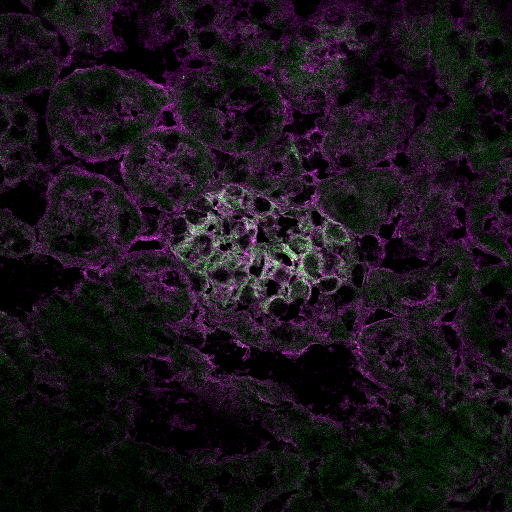

Supplement: Supplementary file 8 — EV and Appendix Figures Source Data [file 44318_2026_759_MOESM8_ESM.zip › SD EV Figureπü«πé│πâÆπéÜπâ╝/Appendix Figure S2/Flox Nephrin-CDKAL1/merge.tif]

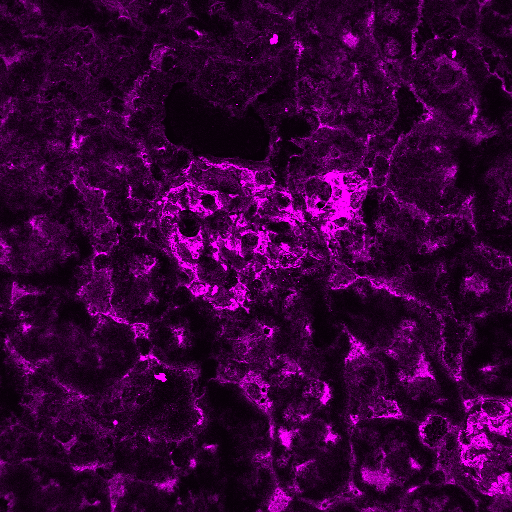

Supplement: Supplementary file 8 — EV and Appendix Figures Source Data [file 44318_2026_759_MOESM8_ESM.zip › SD EV Figureπü«πé│πâÆπéÜπâ╝/Appendix Figure S2/Flox Synaptopodin-CDKAL1/anti-CDKAL1.tif]

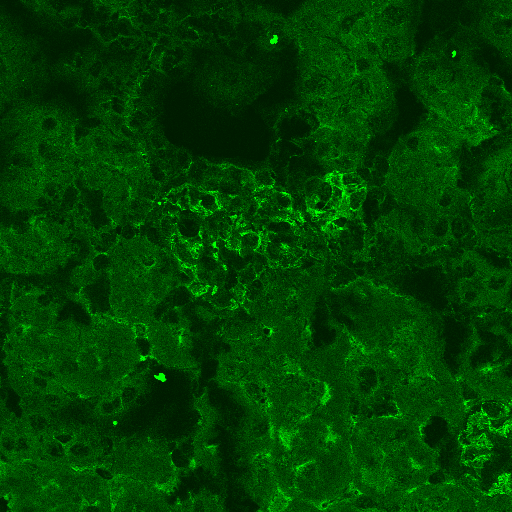

Supplement: Supplementary file 8 — EV and Appendix Figures Source Data [file 44318_2026_759_MOESM8_ESM.zip › SD EV Figureπü«πé│πâÆπéÜπâ╝/Appendix Figure S2/Flox Synaptopodin-CDKAL1/anti-Synaptopodin.tif]

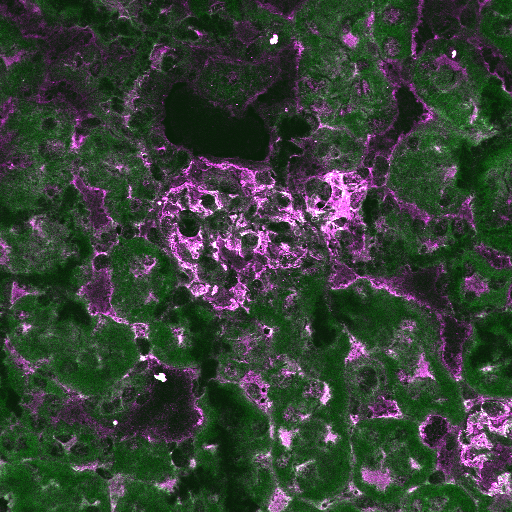

Supplement: Supplementary file 8 — EV and Appendix Figures Source Data [file 44318_2026_759_MOESM8_ESM.zip › SD EV Figureπü«πé│πâÆπéÜπâ╝/Appendix Figure S2/Flox Synaptopodin-CDKAL1/merge.tif]

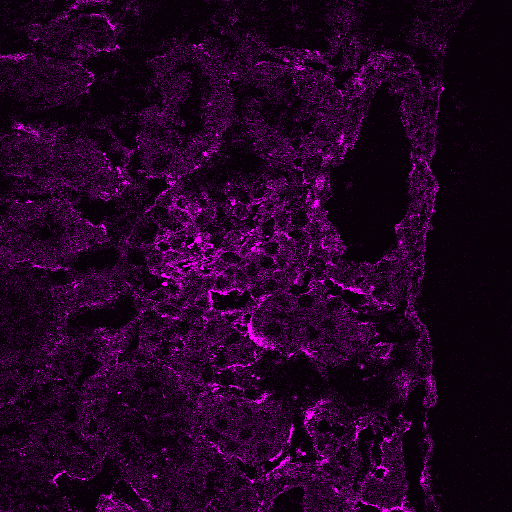

Supplement: Supplementary file 8 — EV and Appendix Figures Source Data [file 44318_2026_759_MOESM8_ESM.zip › SD EV Figureπü«πé│πâÆπéÜπâ╝/Appendix Figure S2/podocyte-specific Cdkal1 KO Synaptopodin-CDKAL1/anti-CDKAL1.tif]

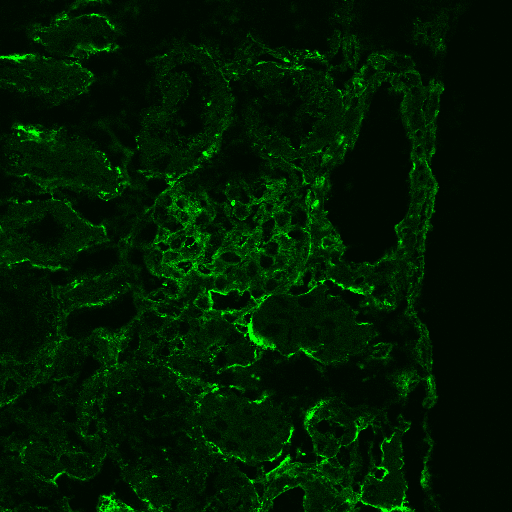

Supplement: Supplementary file 8 — EV and Appendix Figures Source Data [file 44318_2026_759_MOESM8_ESM.zip › SD EV Figureπü«πé│πâÆπéÜπâ╝/Appendix Figure S2/podocyte-specific Cdkal1 KO Synaptopodin-CDKAL1/anti-Synaptopodin.tif]

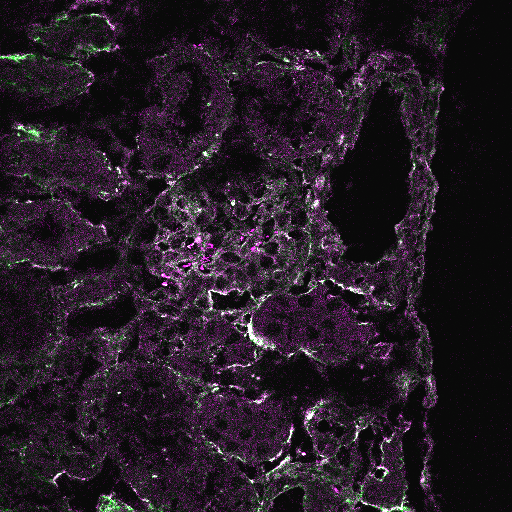

Supplement: Supplementary file 8 — EV and Appendix Figures Source Data [file 44318_2026_759_MOESM8_ESM.zip › SD EV Figureπü«πé│πâÆπéÜπâ╝/Appendix Figure S2/podocyte-specific Cdkal1 KO Synaptopodin-CDKAL1/merge.tif]

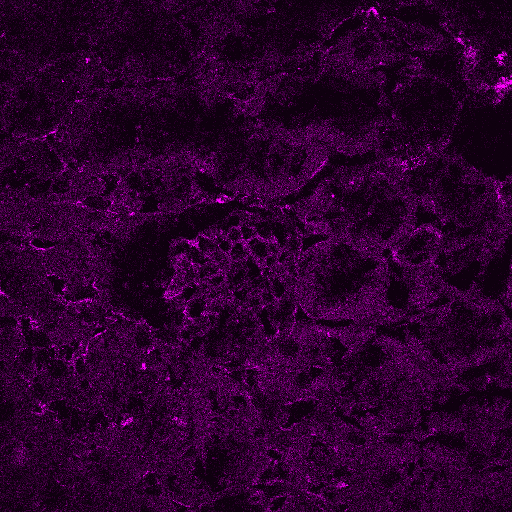

Supplement: Supplementary file 8 — EV and Appendix Figures Source Data [file 44318_2026_759_MOESM8_ESM.zip › SD EV Figureπü«πé│πâÆπéÜπâ╝/Appendix Figure S2/podocyte-specific Cdkal1 KO Nephrin-CDKAL1/anti-CDKAL1.tif]

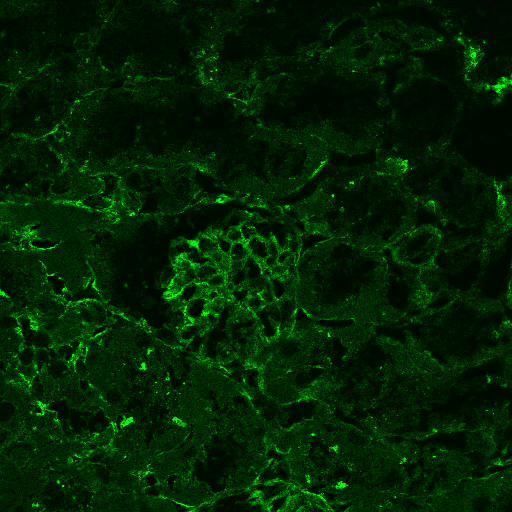

Supplement: Supplementary file 8 — EV and Appendix Figures Source Data [file 44318_2026_759_MOESM8_ESM.zip › SD EV Figureπü«πé│πâÆπéÜπâ╝/Appendix Figure S2/podocyte-specific Cdkal1 KO Nephrin-CDKAL1/anti-Nephrin.tif]

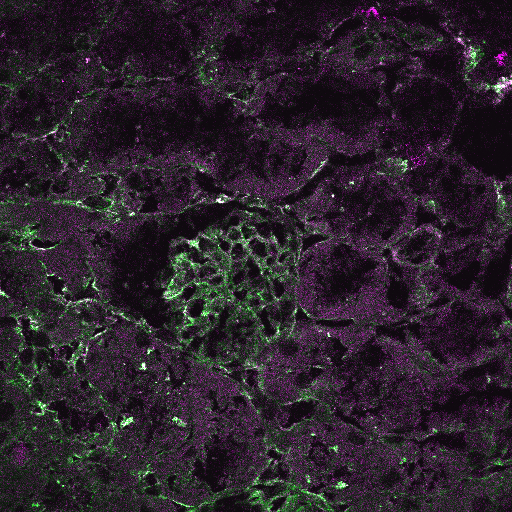

Supplement: Supplementary file 8 — EV and Appendix Figures Source Data [file 44318_2026_759_MOESM8_ESM.zip › SD EV Figureπü«πé│πâÆπéÜπâ╝/Appendix Figure S2/podocyte-specific Cdkal1 KO Nephrin-CDKAL1/merge.tif]

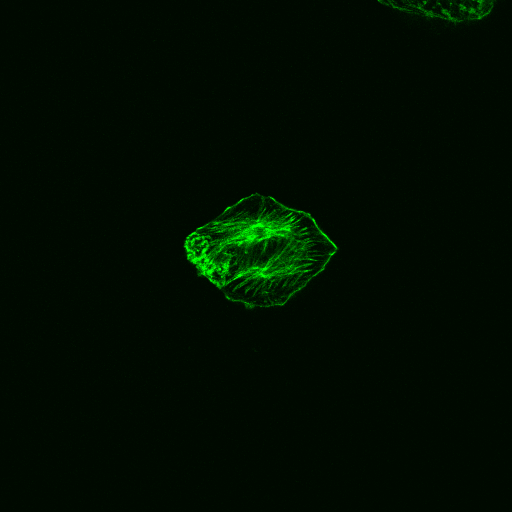

Supplement: Supplementary file 8 — EV and Appendix Figures Source Data [file 44318_2026_759_MOESM8_ESM.zip › SD EV Figureπü«πé│πâÆπéÜπâ╝/Appendix Figure S5/Appendix Figure S5B/SgControl.tif]

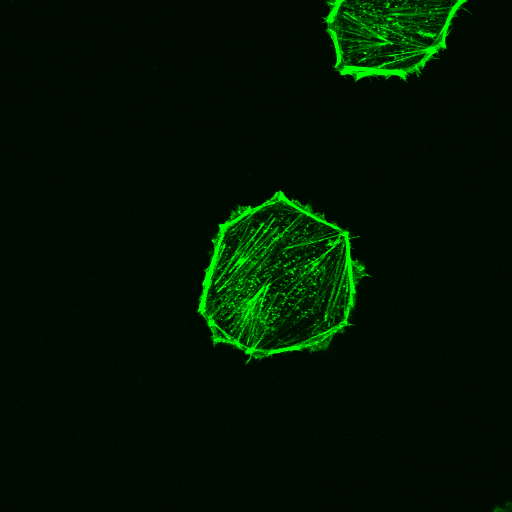

Supplement: Supplementary file 8 — EV and Appendix Figures Source Data [file 44318_2026_759_MOESM8_ESM.zip › SD EV Figureπü«πé│πâÆπéÜπâ╝/Appendix Figure S5/Appendix Figure S5B/KO2.tif]

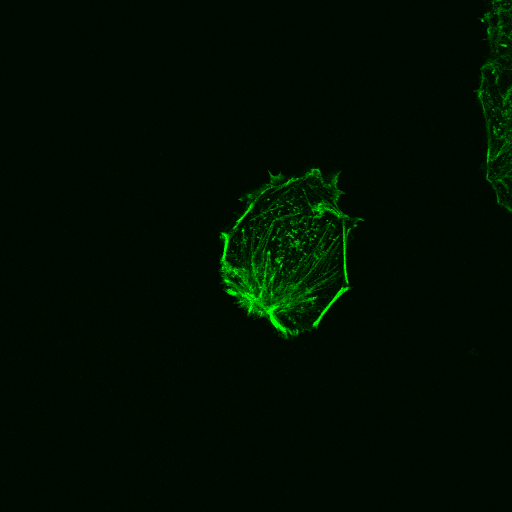

Supplement: Supplementary file 8 — EV and Appendix Figures Source Data [file 44318_2026_759_MOESM8_ESM.zip › SD EV Figureπü«πé│πâÆπéÜπâ╝/Appendix Figure S5/Appendix Figure S5B/KO1.tif]

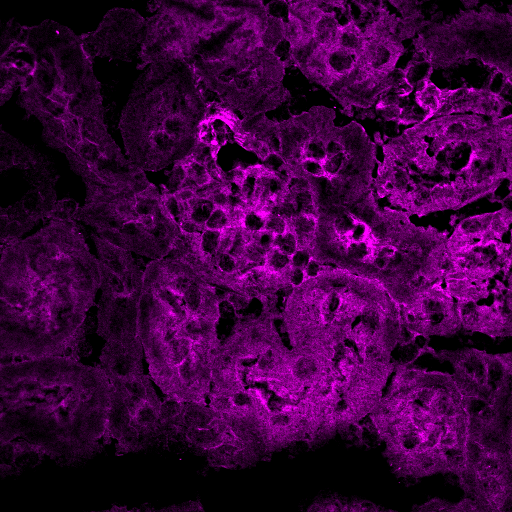

Supplement: Supplementary file 8 — EV and Appendix Figures Source Data [file 44318_2026_759_MOESM8_ESM.zip › SD EV Figureπü«πé│πâÆπéÜπâ╝/SD EV3/EV 3A/Flox.tif]

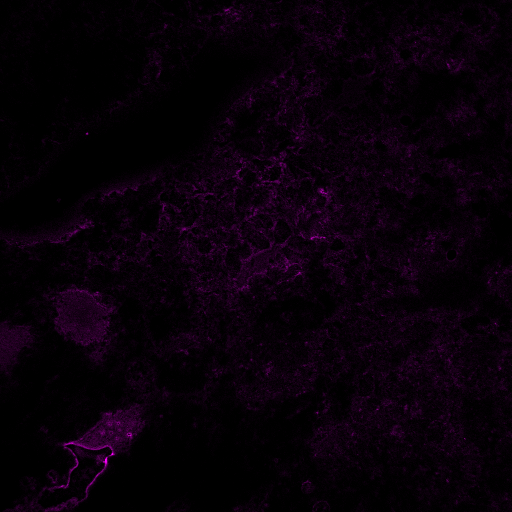

Supplement: Supplementary file 8 — EV and Appendix Figures Source Data [file 44318_2026_759_MOESM8_ESM.zip › SD EV Figureπü«πé│πâÆπéÜπâ╝/SD EV3/EV 3A/Systemic Cdkal1 KO.tif]

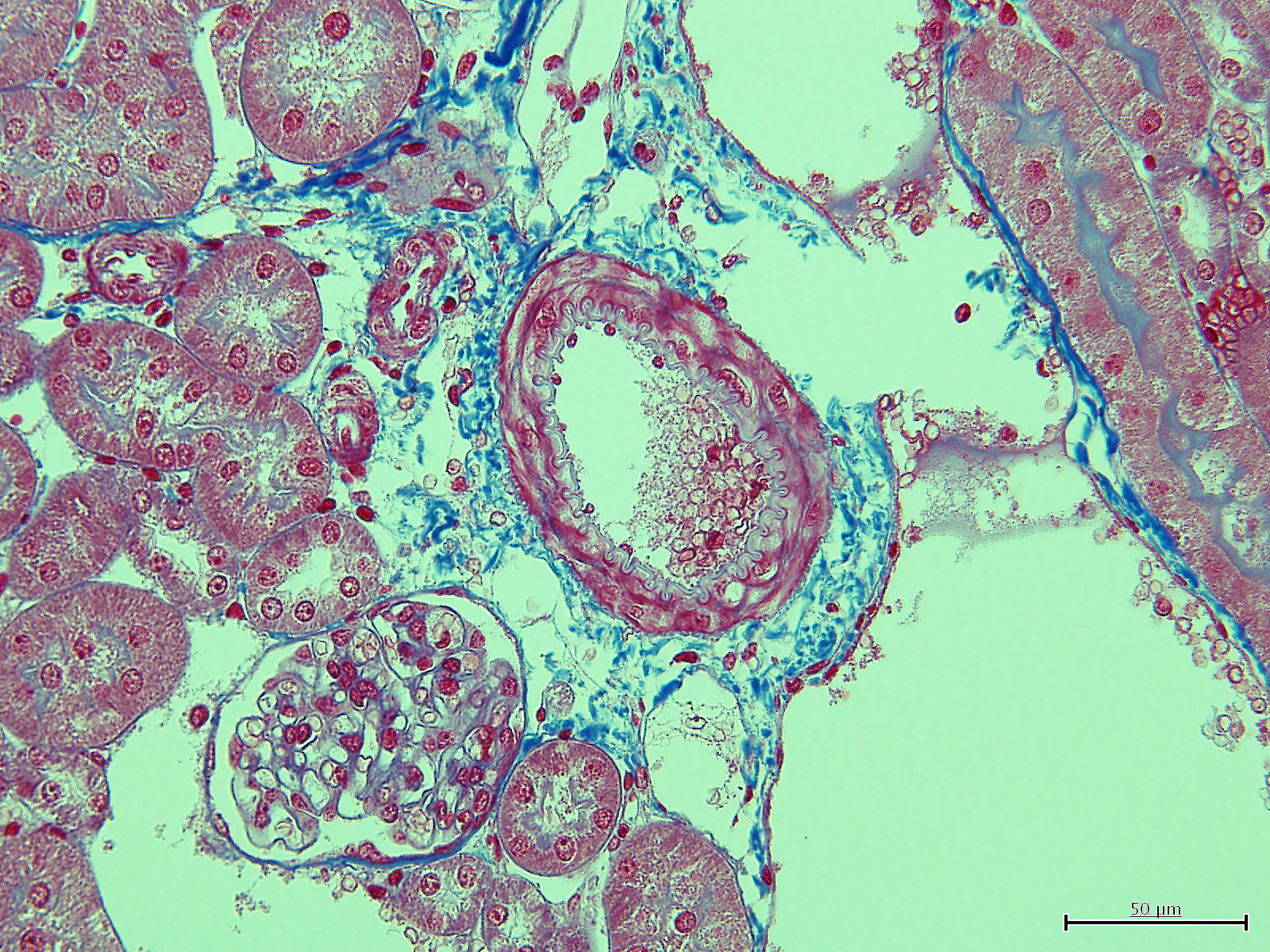

Supplement: Supplementary file 8 — EV and Appendix Figures Source Data [file 44318_2026_759_MOESM8_ESM.zip › SD EV Figureπü«πé│πâÆπéÜπâ╝/SD EV2/EV 2B/Flox_HighMagnification.tif]

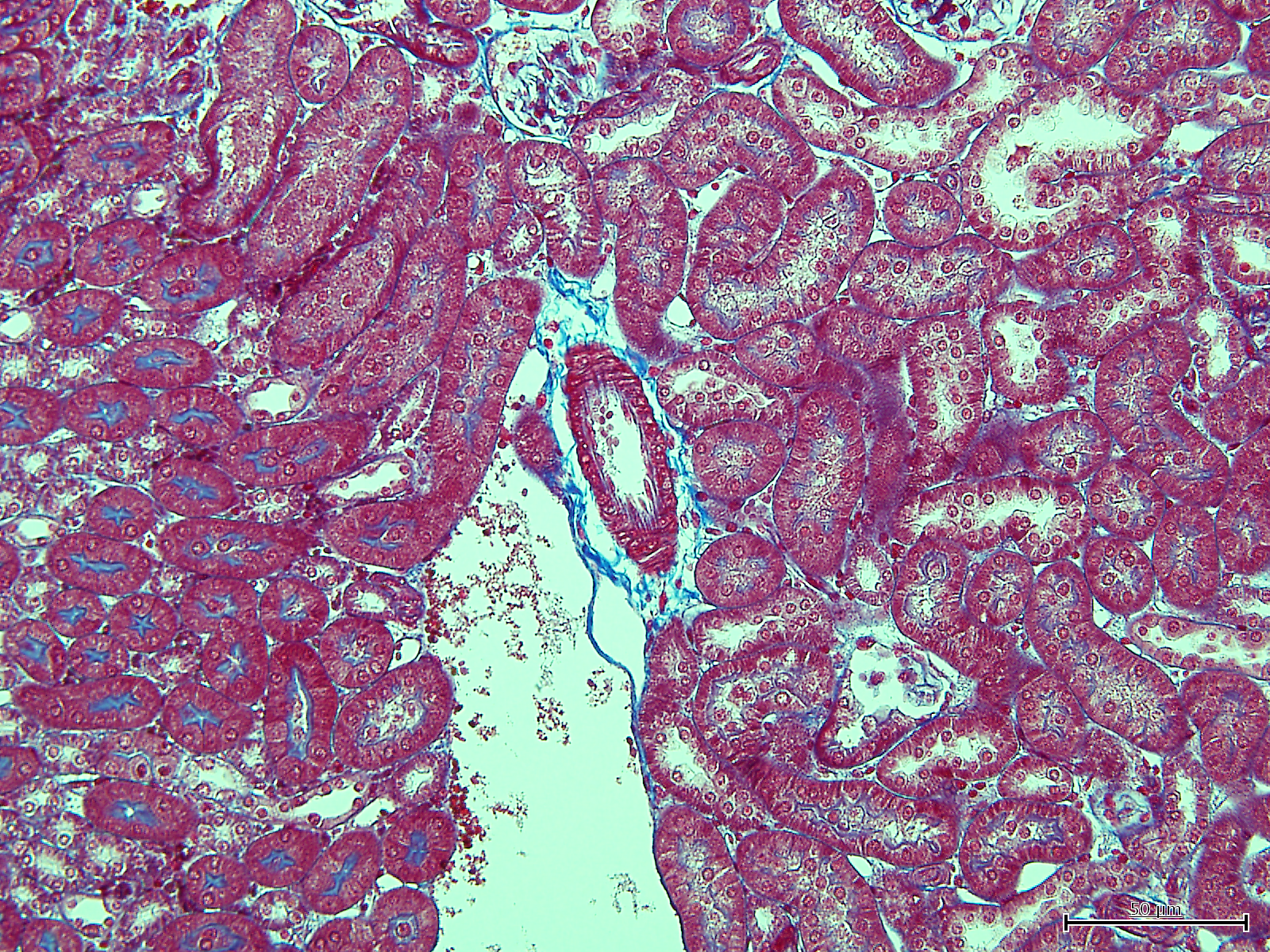

Supplement: Supplementary file 8 — EV and Appendix Figures Source Data [file 44318_2026_759_MOESM8_ESM.zip › SD EV Figureπü«πé│πâÆπéÜπâ╝/SD EV2/EV 2B/Podocyte-specific Cdkal1 KO_HighMagnification.tif]

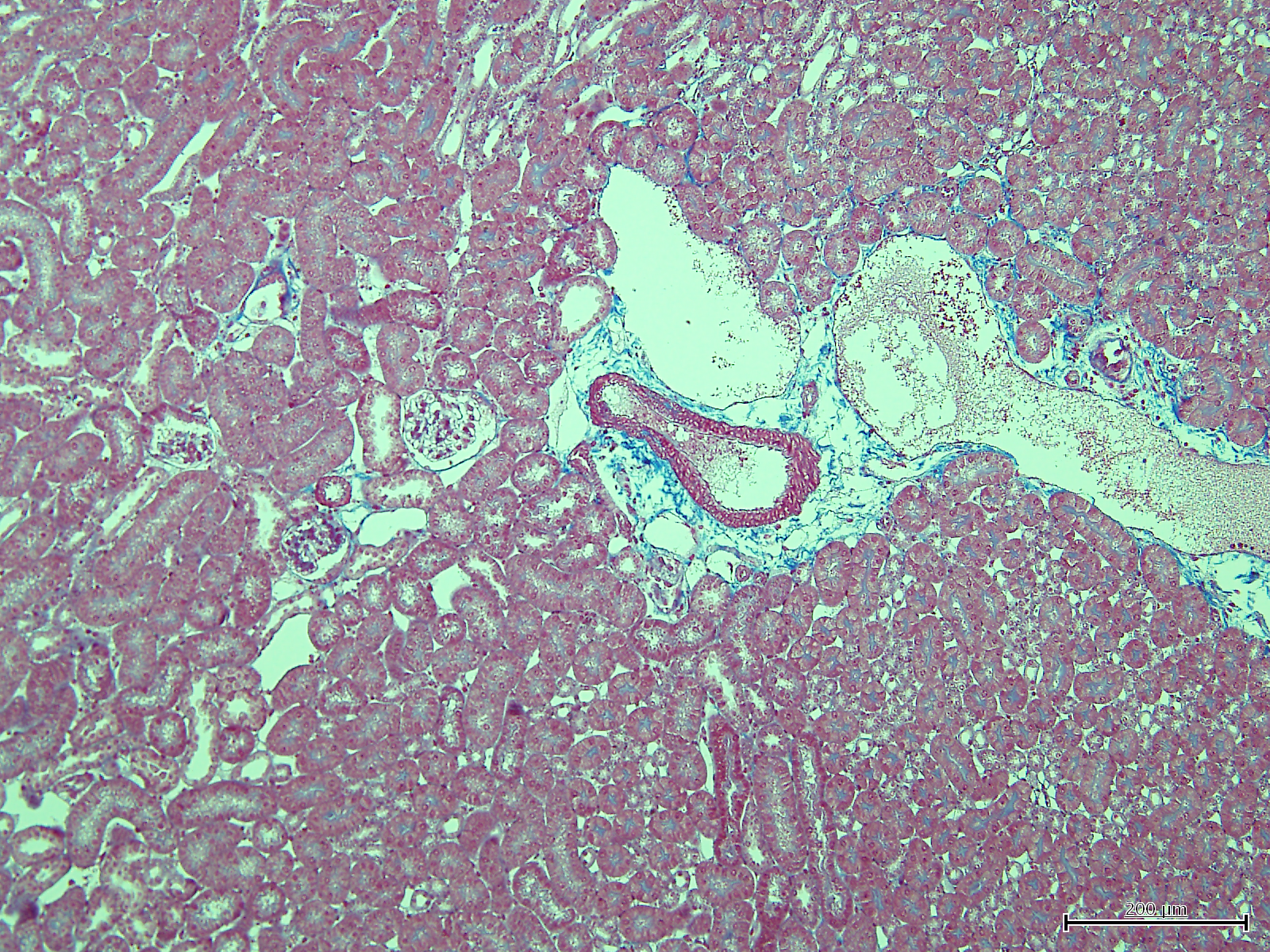

Supplement: Supplementary file 8 — EV and Appendix Figures Source Data [file 44318_2026_759_MOESM8_ESM.zip › SD EV Figureπü«πé│πâÆπéÜπâ╝/SD EV2/EV 2B/Flox_LowMagnification.tif]

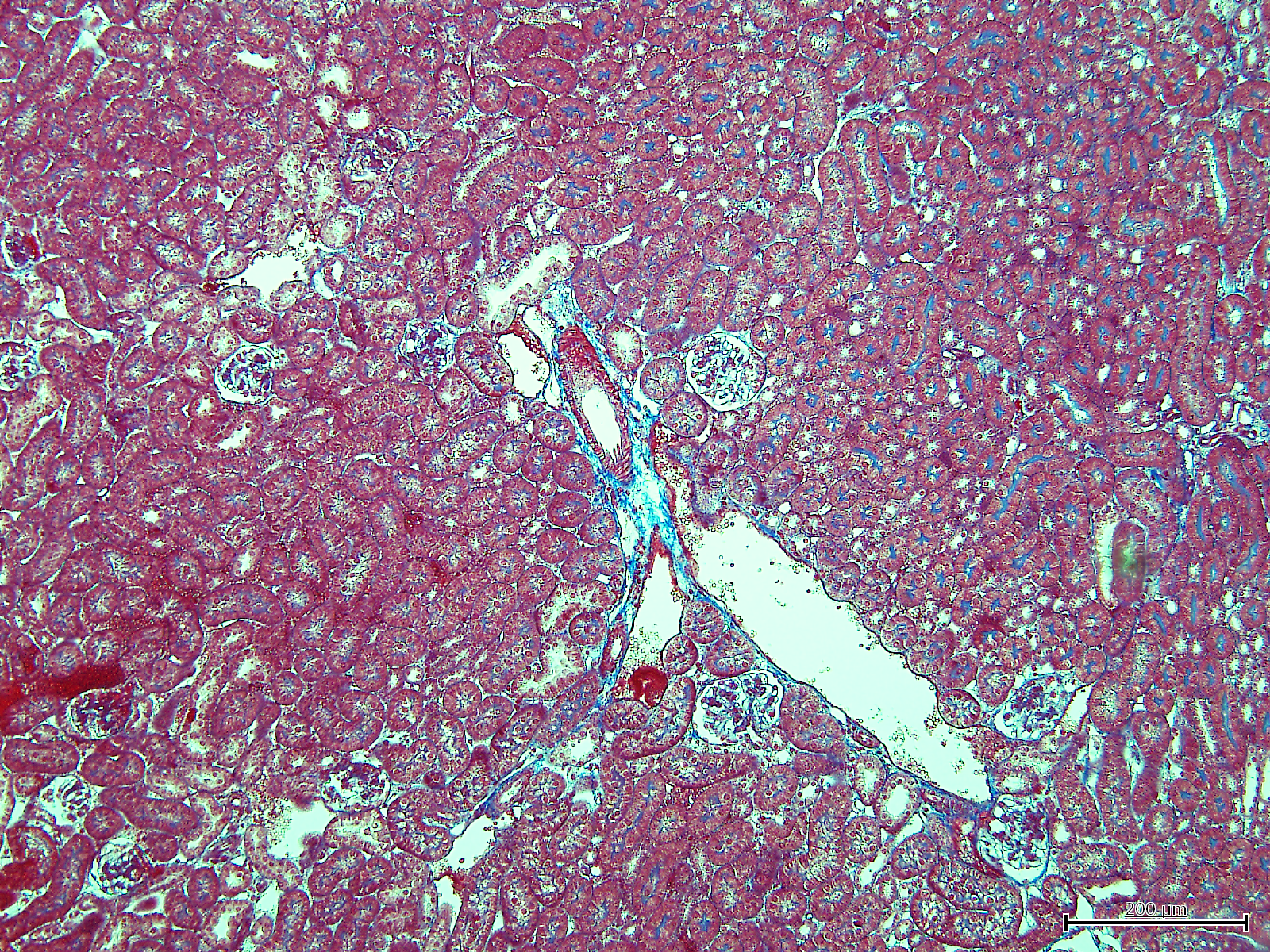

Supplement: Supplementary file 8 — EV and Appendix Figures Source Data [file 44318_2026_759_MOESM8_ESM.zip › SD EV Figureπü«πé│πâÆπéÜπâ╝/SD EV2/EV 2B/Podocyte-specific Cdkal1 KO_LowMagnification.tif]

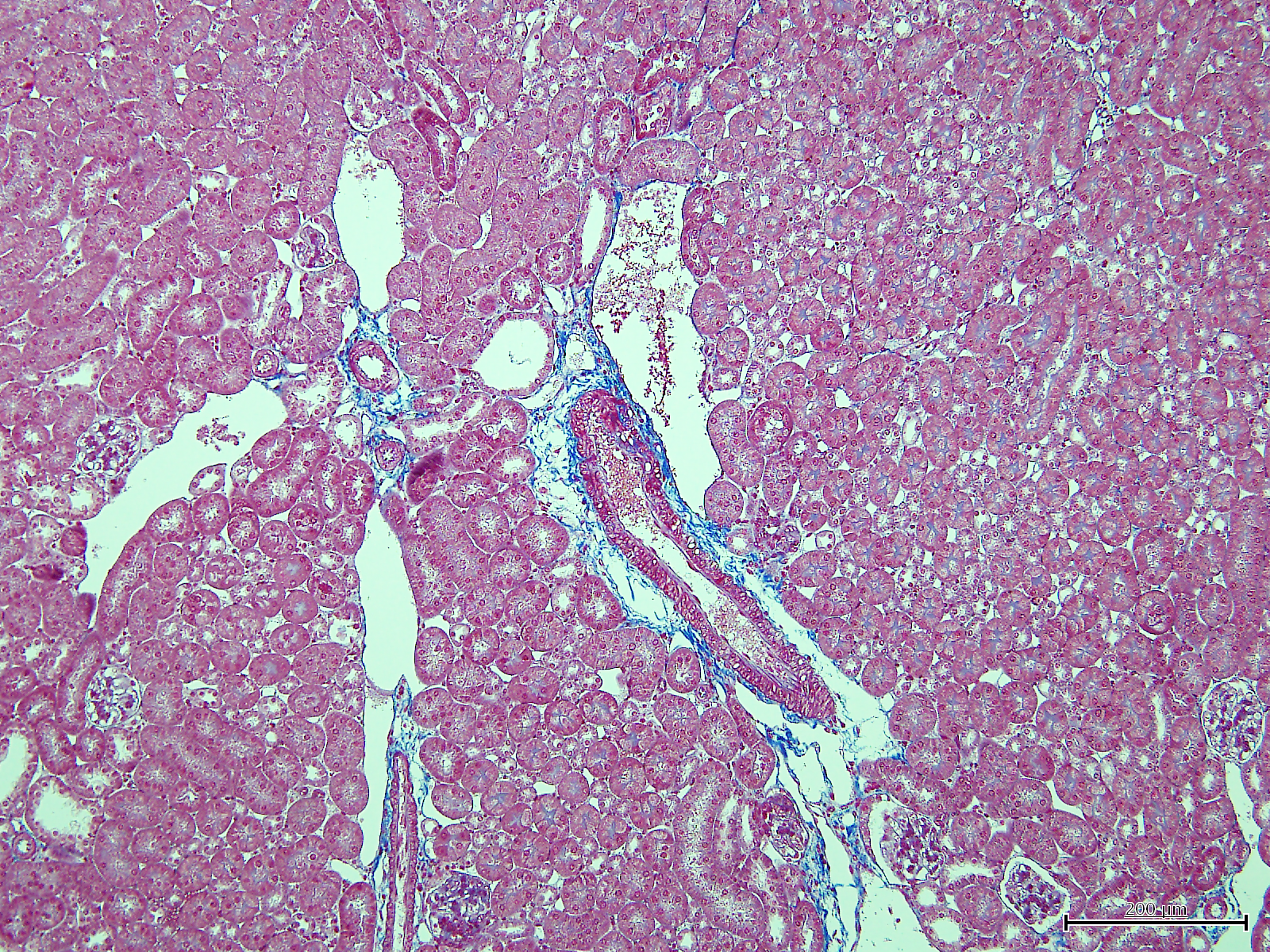

Supplement: Supplementary file 8 — EV and Appendix Figures Source Data [file 44318_2026_759_MOESM8_ESM.zip › SD EV Figureπü«πé│πâÆπéÜπâ╝/SD EV2/EV 2B/KO_LowMagnification.tif]

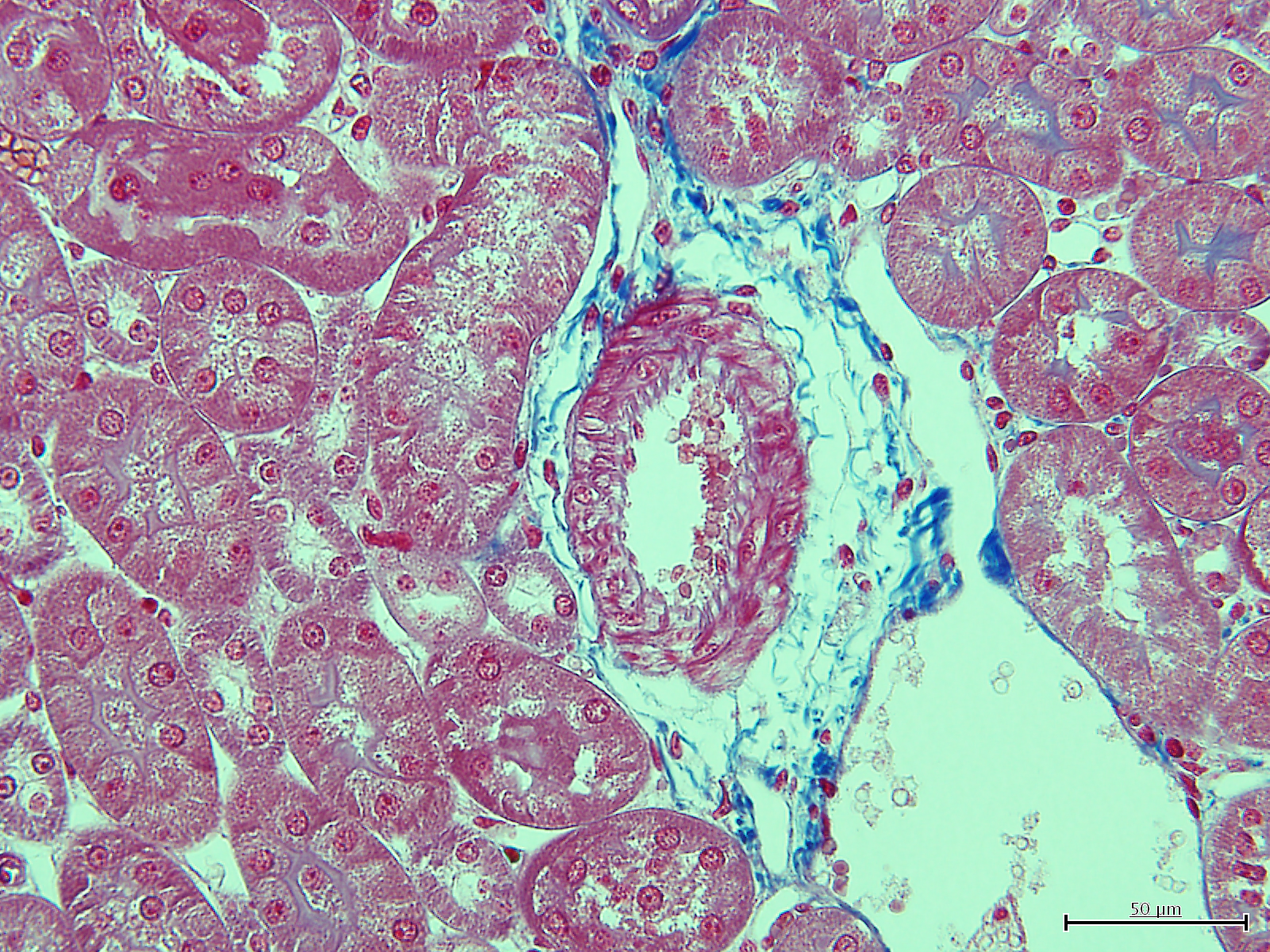

Supplement: Supplementary file 8 — EV and Appendix Figures Source Data [file 44318_2026_759_MOESM8_ESM.zip › SD EV Figureπü«πé│πâÆπéÜπâ╝/SD EV2/EV 2B/KO_HighMagnification.tif]

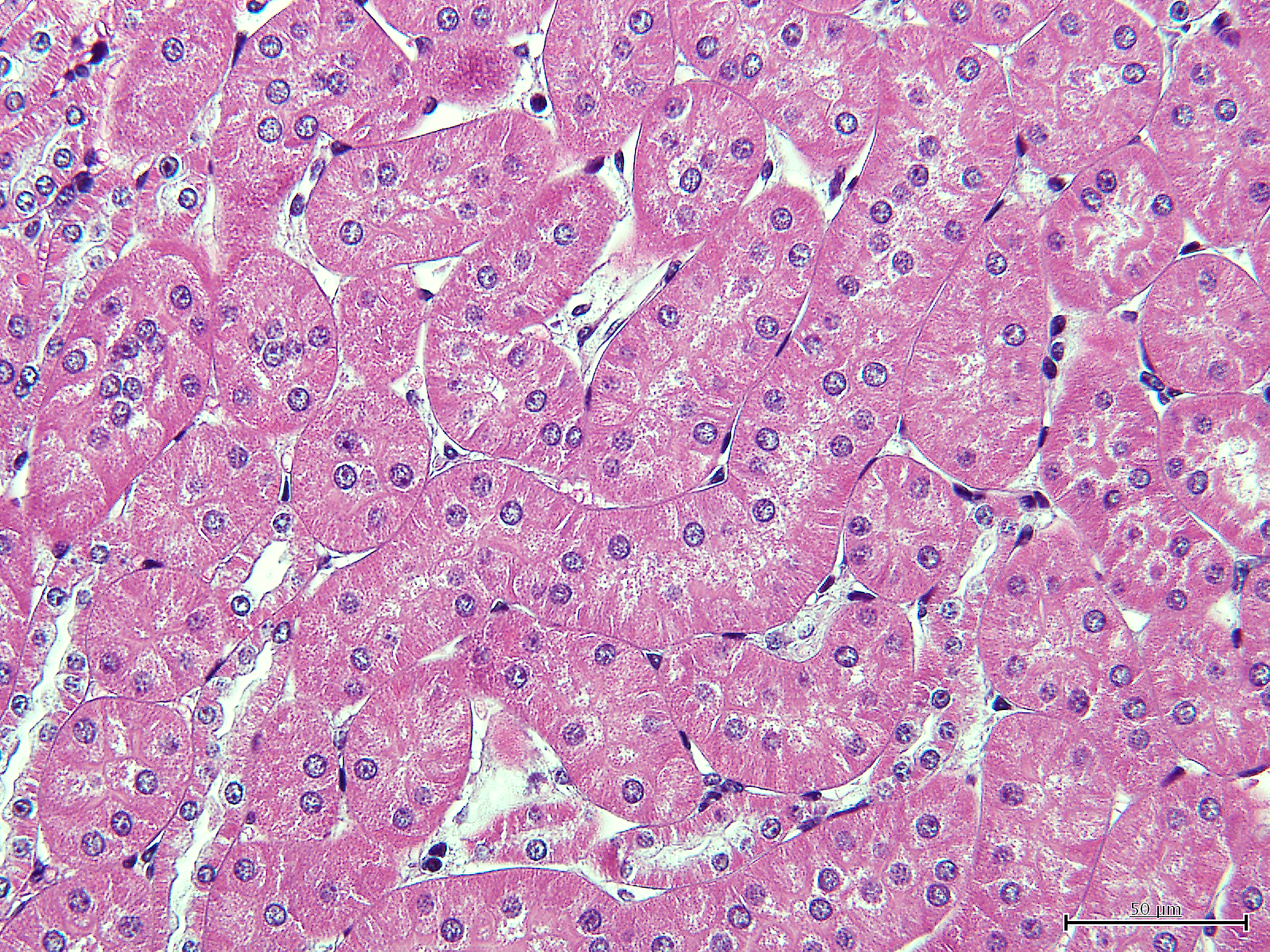

Supplement: Supplementary file 8 — EV and Appendix Figures Source Data [file 44318_2026_759_MOESM8_ESM.zip › SD EV Figureπü«πé│πâÆπéÜπâ╝/SD EV2/EV 2A/Flox_HighMagnification.tif]

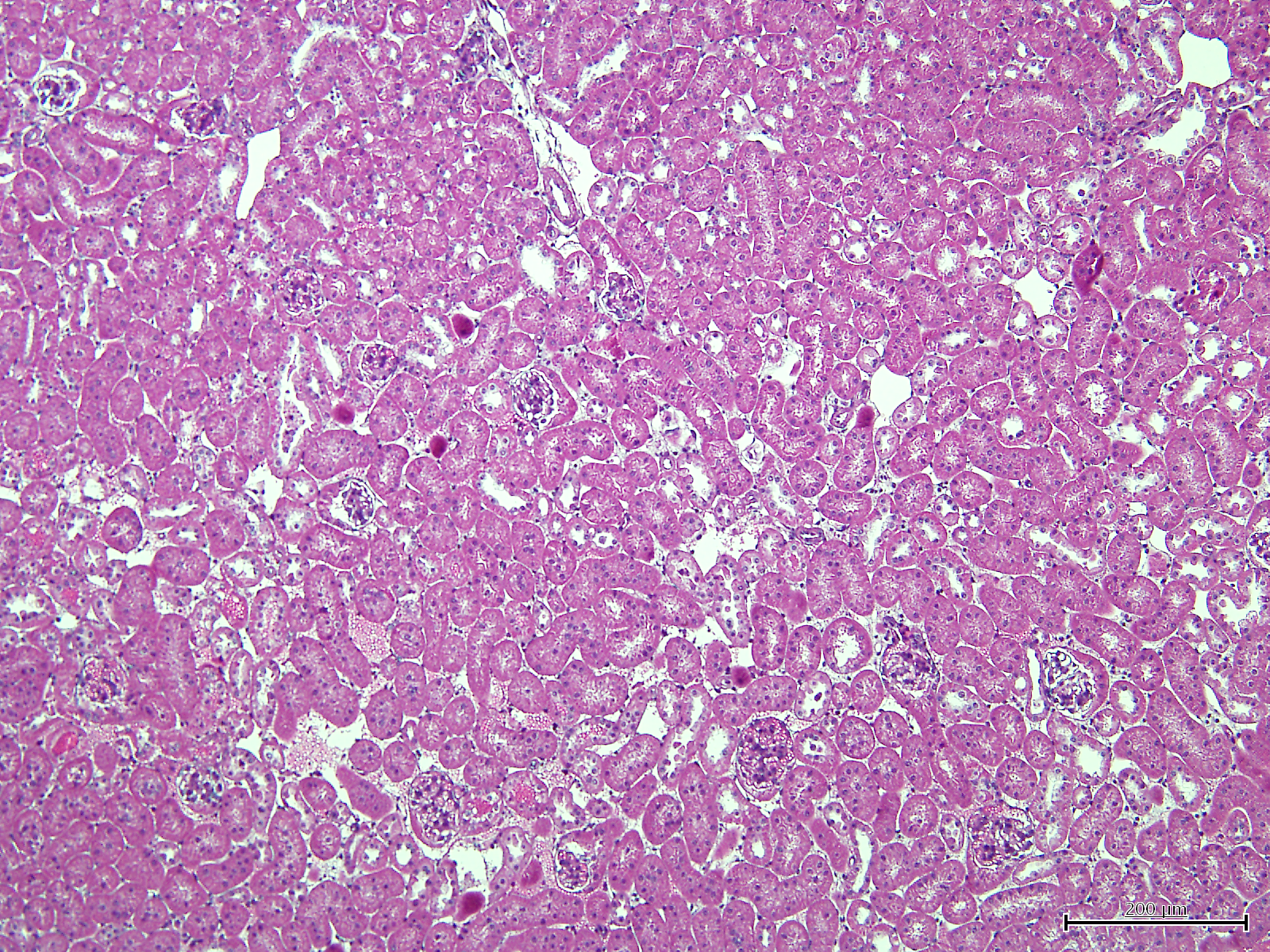

Supplement: Supplementary file 8 — EV and Appendix Figures Source Data [file 44318_2026_759_MOESM8_ESM.zip › SD EV Figureπü«πé│πâÆπéÜπâ╝/SD EV2/EV 2A/KO_LowMaginification.tif]

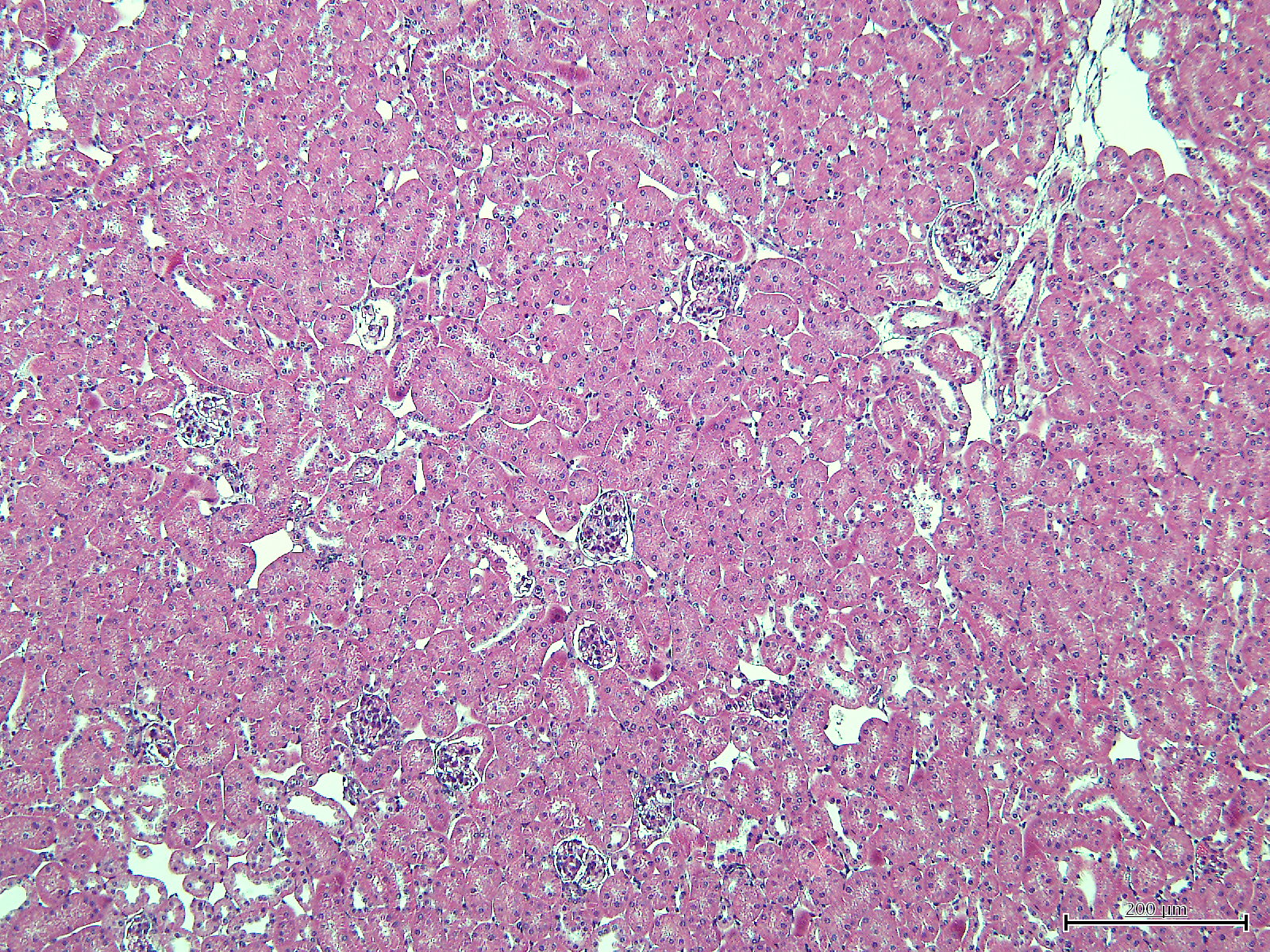

Supplement: Supplementary file 8 — EV and Appendix Figures Source Data [file 44318_2026_759_MOESM8_ESM.zip › SD EV Figureπü«πé│πâÆπéÜπâ╝/SD EV2/EV 2A/Flox_LowMagnification.tif]

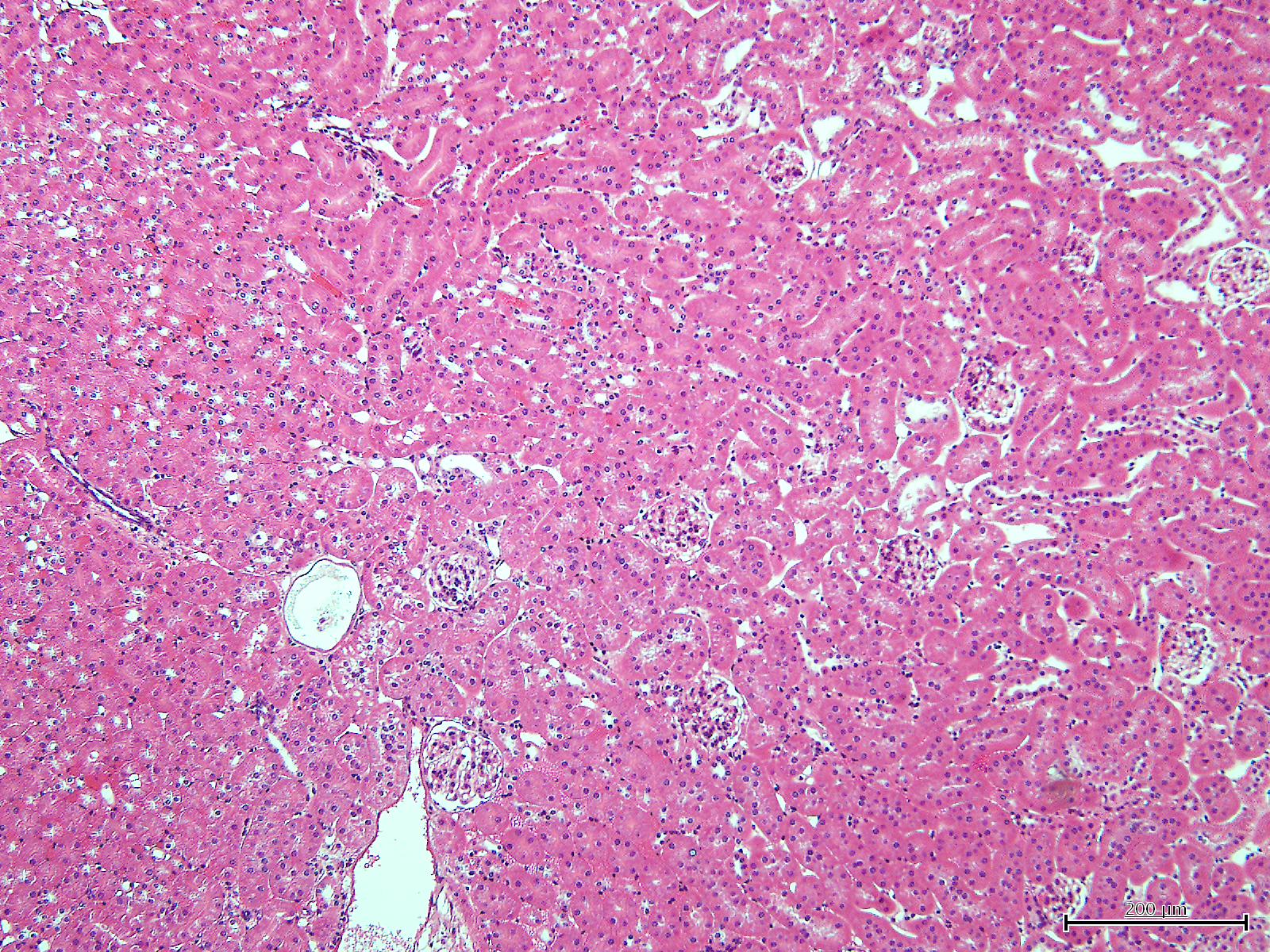

Supplement: Supplementary file 8 — EV and Appendix Figures Source Data [file 44318_2026_759_MOESM8_ESM.zip › SD EV Figureπü«πé│πâÆπéÜπâ╝/SD EV2/EV 2A/Podocyte-specific Cdkal1 KO_LowMagnification.TIF]

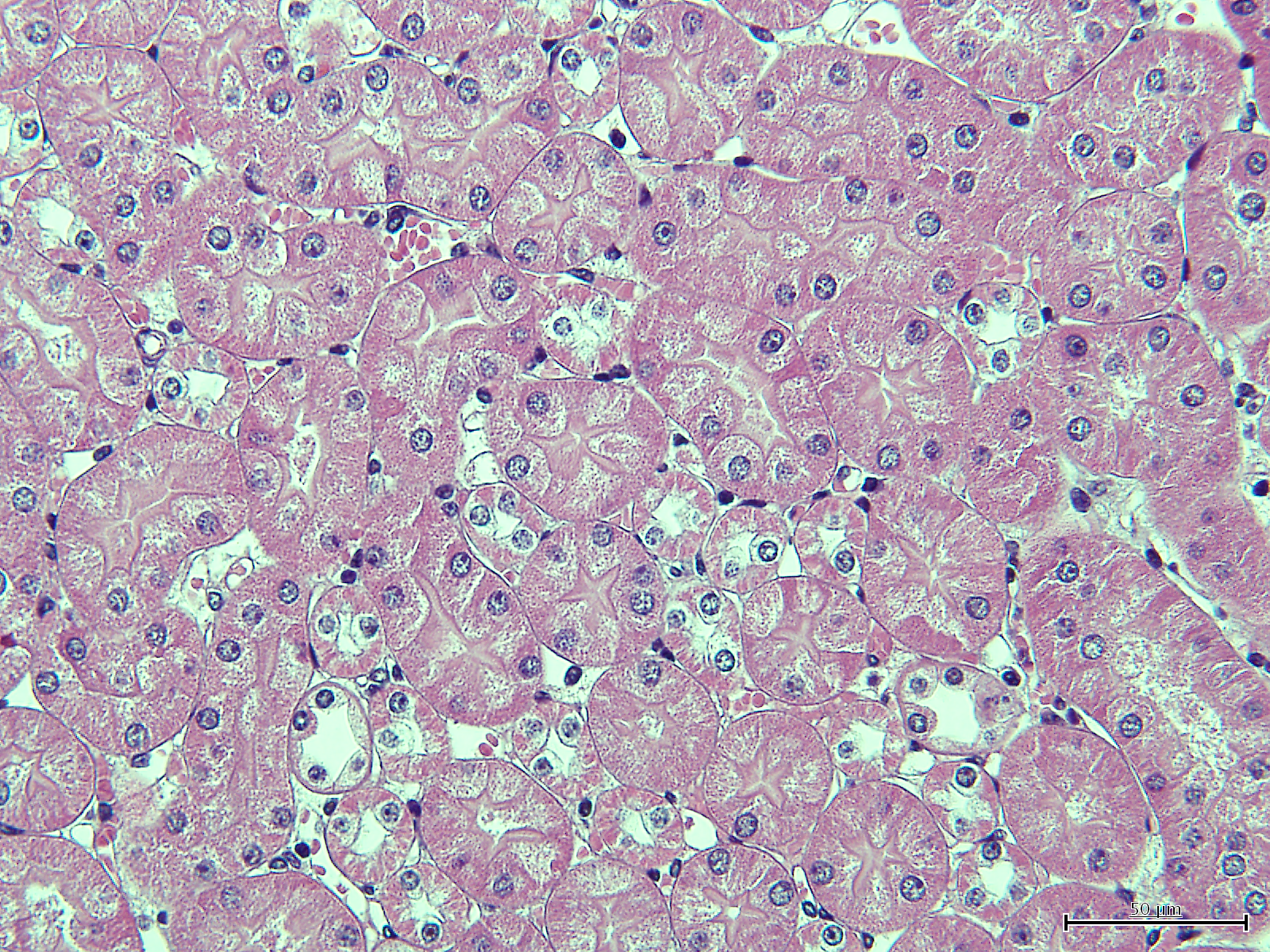

Supplement: Supplementary file 8 — EV and Appendix Figures Source Data [file 44318_2026_759_MOESM8_ESM.zip › SD EV Figureπü«πé│πâÆπéÜπâ╝/SD EV2/EV 2A/KO_HighMagnification.tif]

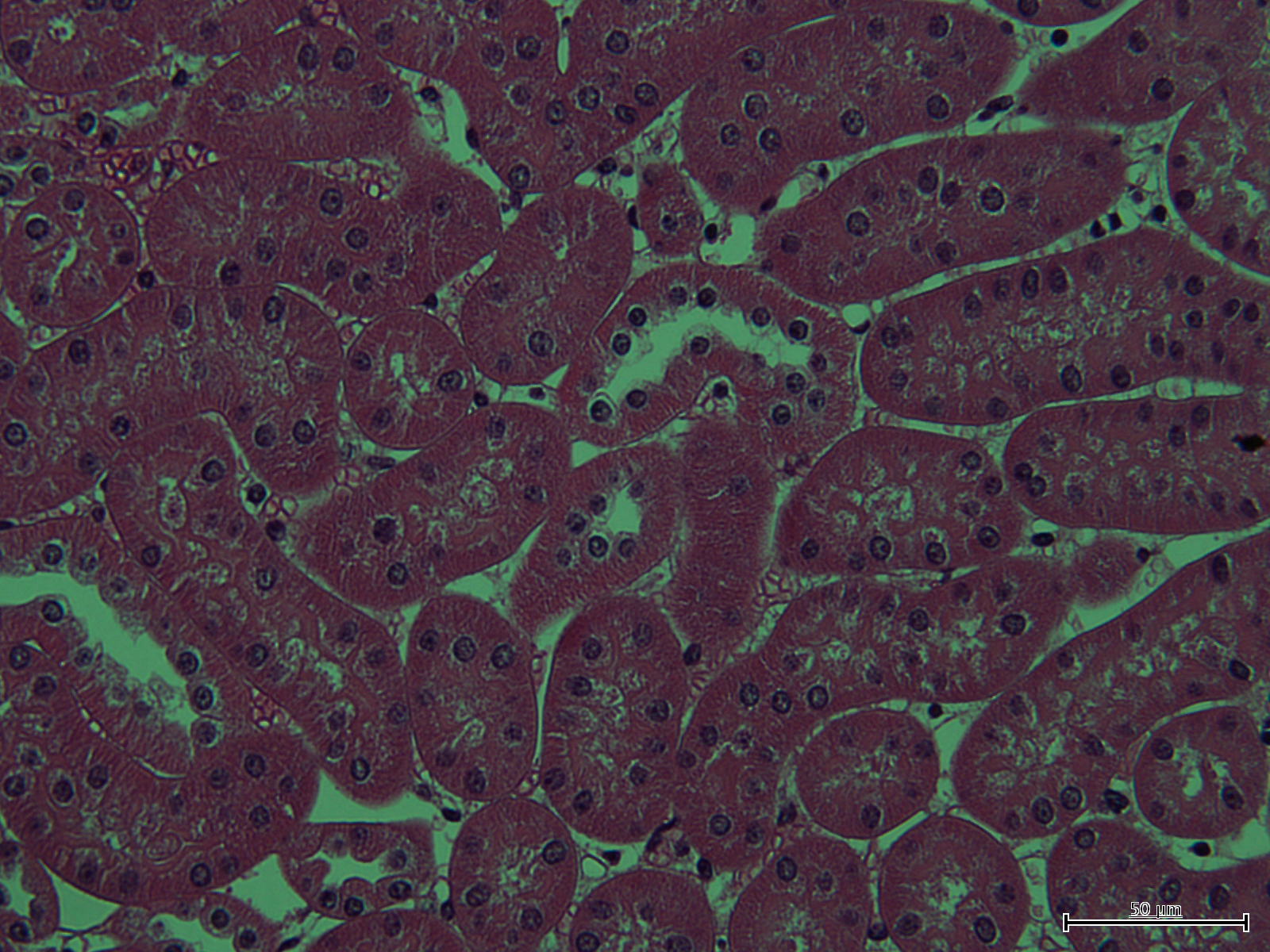

Supplement: Supplementary file 8 — EV and Appendix Figures Source Data [file 44318_2026_759_MOESM8_ESM.zip › SD EV Figureπü«πé│πâÆπéÜπâ╝/SD EV2/EV 2A/Podocyte-specific Cdkal1 KO_HighMagnification..tif]

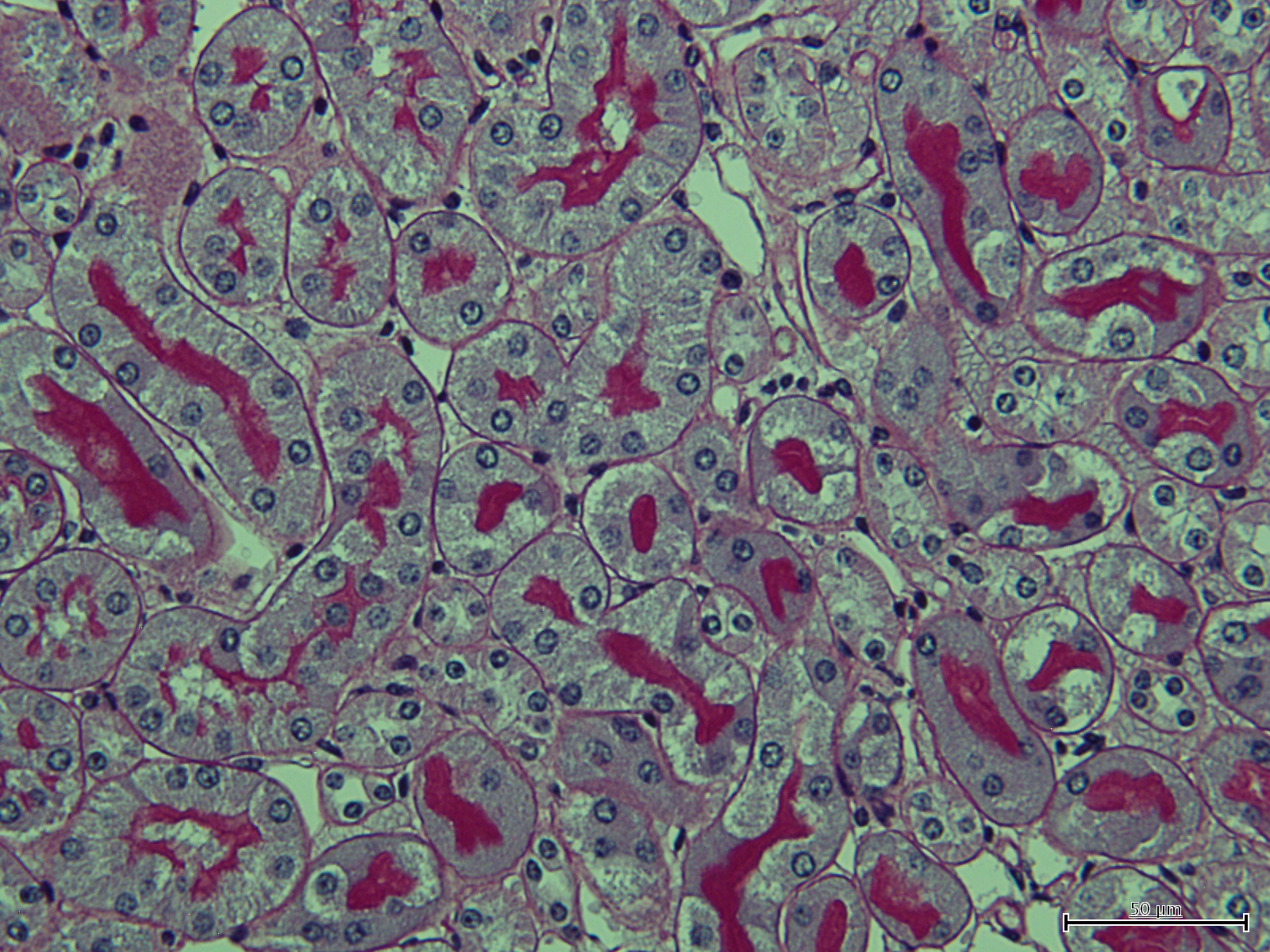

Supplement: Supplementary file 8 — EV and Appendix Figures Source Data [file 44318_2026_759_MOESM8_ESM.zip › SD EV Figureπü«πé│πâÆπéÜπâ╝/SD EV3/EV 3C/Systemic Cdkal1 KO/KO_HighMagnification2.TIF]

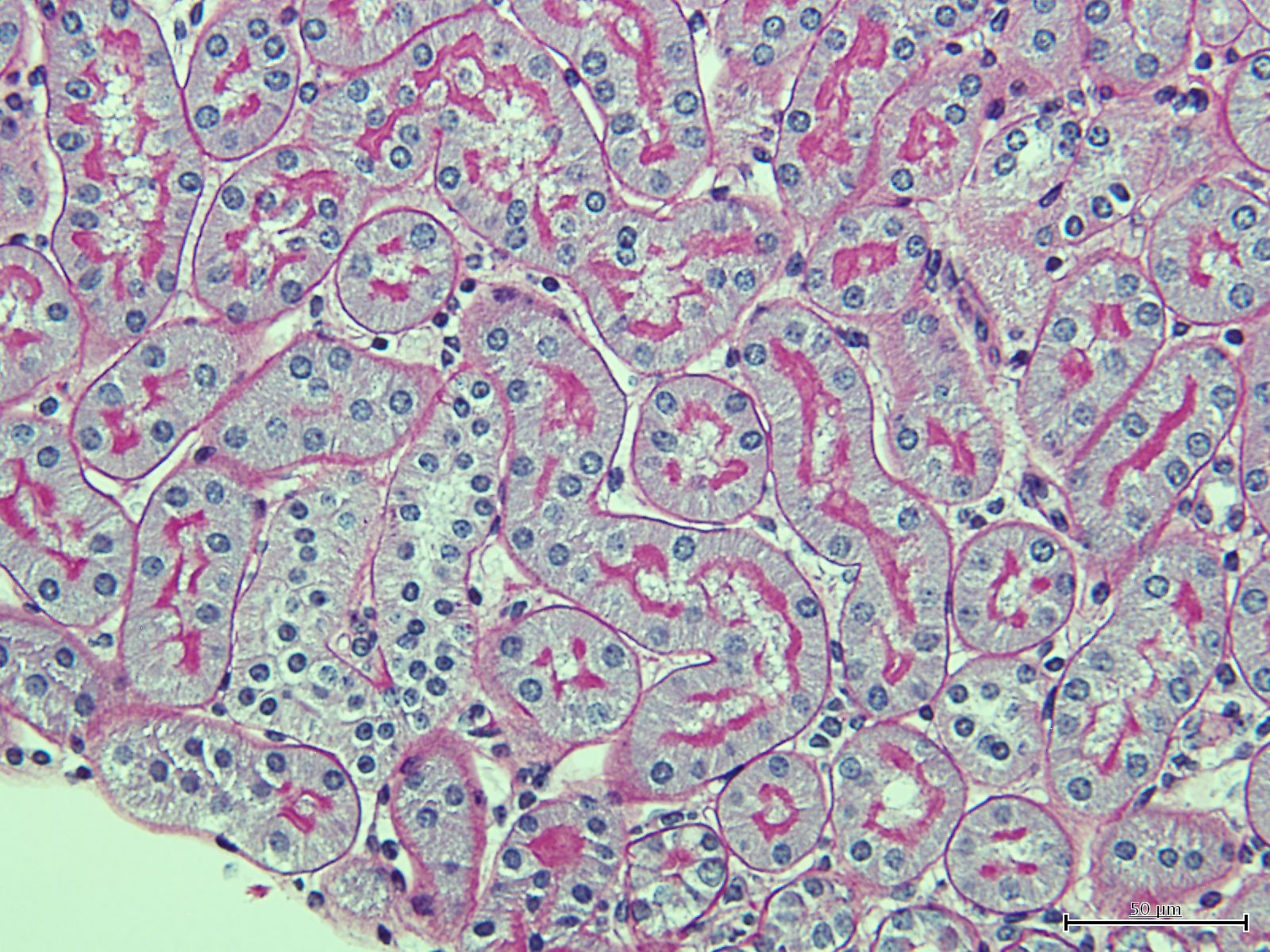

Supplement: Supplementary file 8 — EV and Appendix Figures Source Data [file 44318_2026_759_MOESM8_ESM.zip › SD EV Figureπü«πé│πâÆπéÜπâ╝/SD EV3/EV 3C/Systemic Cdkal1 KO/KO_HighMagnification1.TIF]

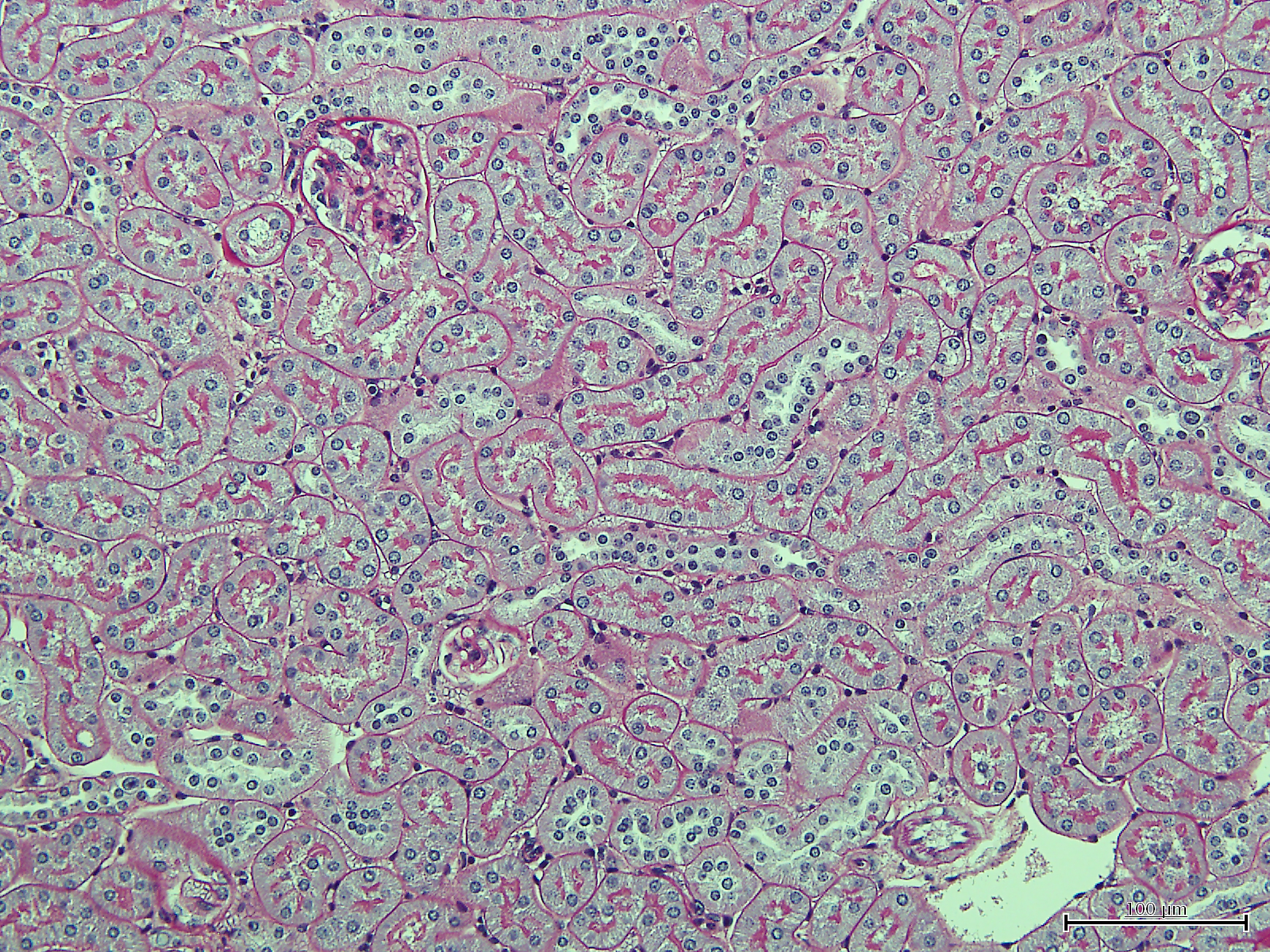

Supplement: Supplementary file 8 — EV and Appendix Figures Source Data [file 44318_2026_759_MOESM8_ESM.zip › SD EV Figureπü«πé│πâÆπéÜπâ╝/SD EV3/EV 3C/Systemic Cdkal1 KO/KO_MediumMagnification.TIF]

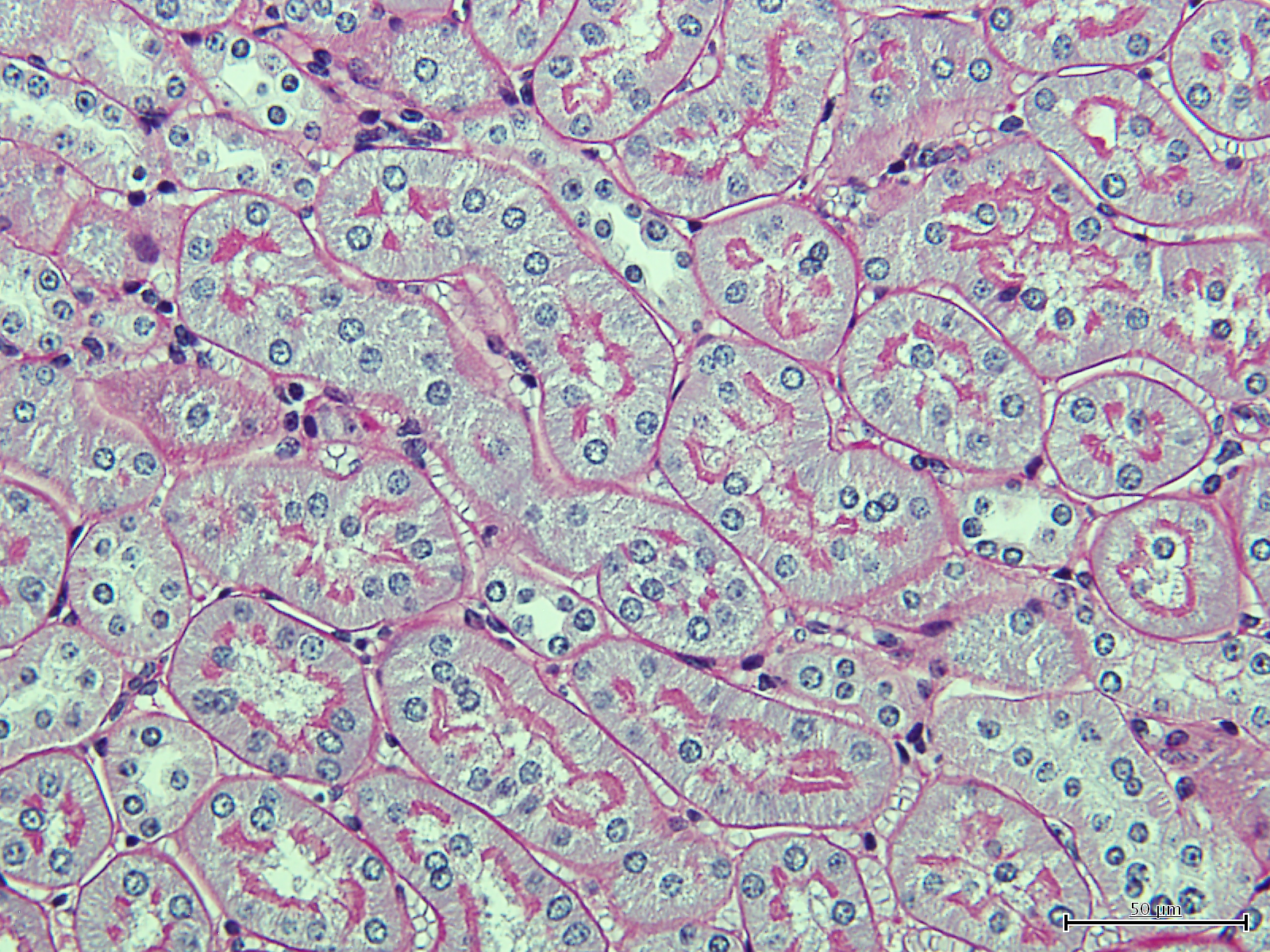

Supplement: Supplementary file 8 — EV and Appendix Figures Source Data [file 44318_2026_759_MOESM8_ESM.zip › SD EV Figureπü«πé│πâÆπéÜπâ╝/SD EV3/EV 3C/Flox/Flox_HighMagnification2.TIF]

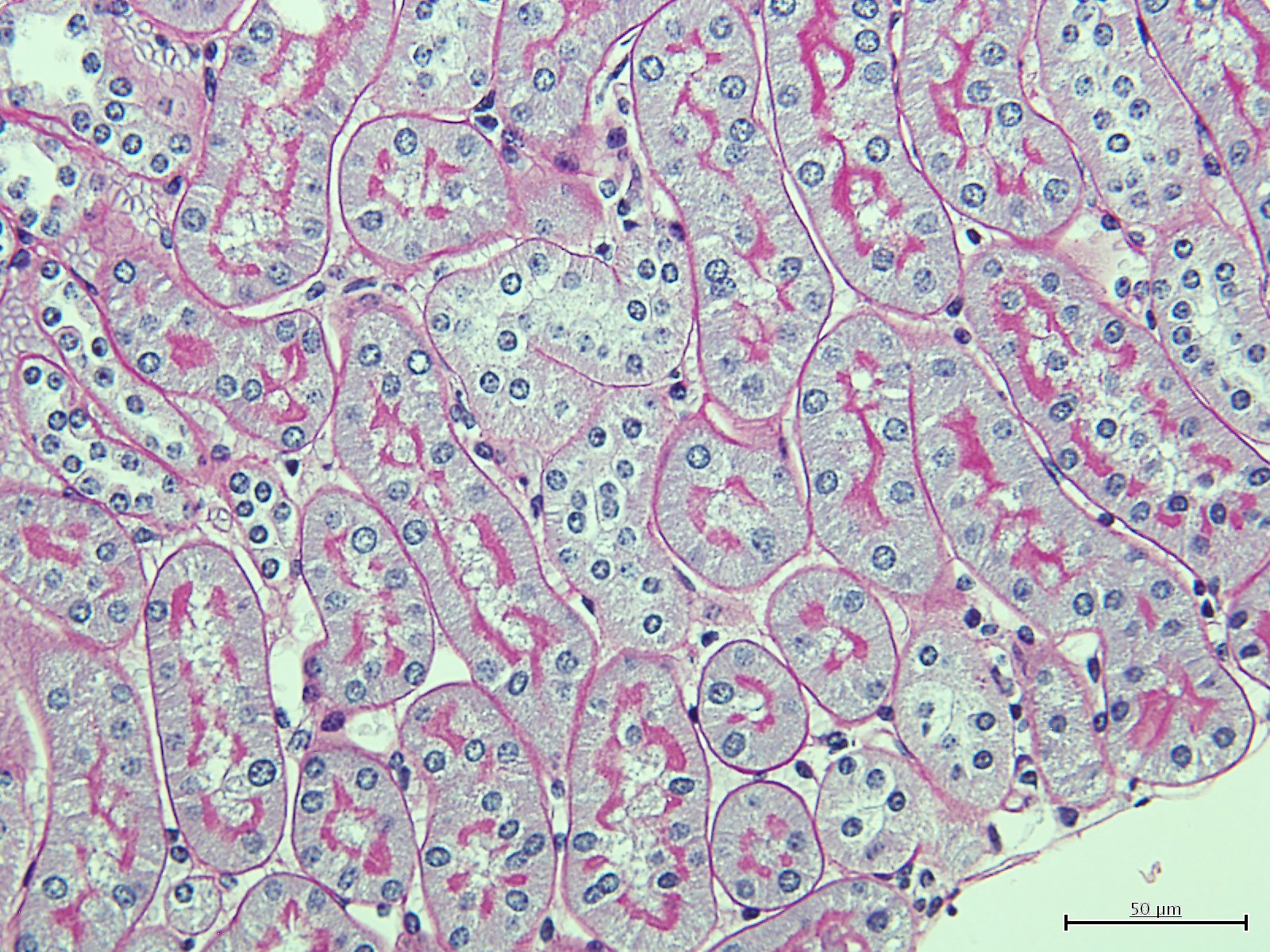

Supplement: Supplementary file 8 — EV and Appendix Figures Source Data [file 44318_2026_759_MOESM8_ESM.zip › SD EV Figureπü«πé│πâÆπéÜπâ╝/SD EV3/EV 3C/Flox/Flox_HighMagnification1.TIF]
