# Supplementary figures and images for: CDKAL1 dysfunction impairs lysine codon translation in podocytes and accelerates chronic kidney disease (part 2 of 2)
Source: EMBO J. 2026 Mar 28;45(9):3206–29. doi: 10.1038/s44318-026-00759-3 (PMC13144697; doi:10.1038/s44318-026-00759-3)

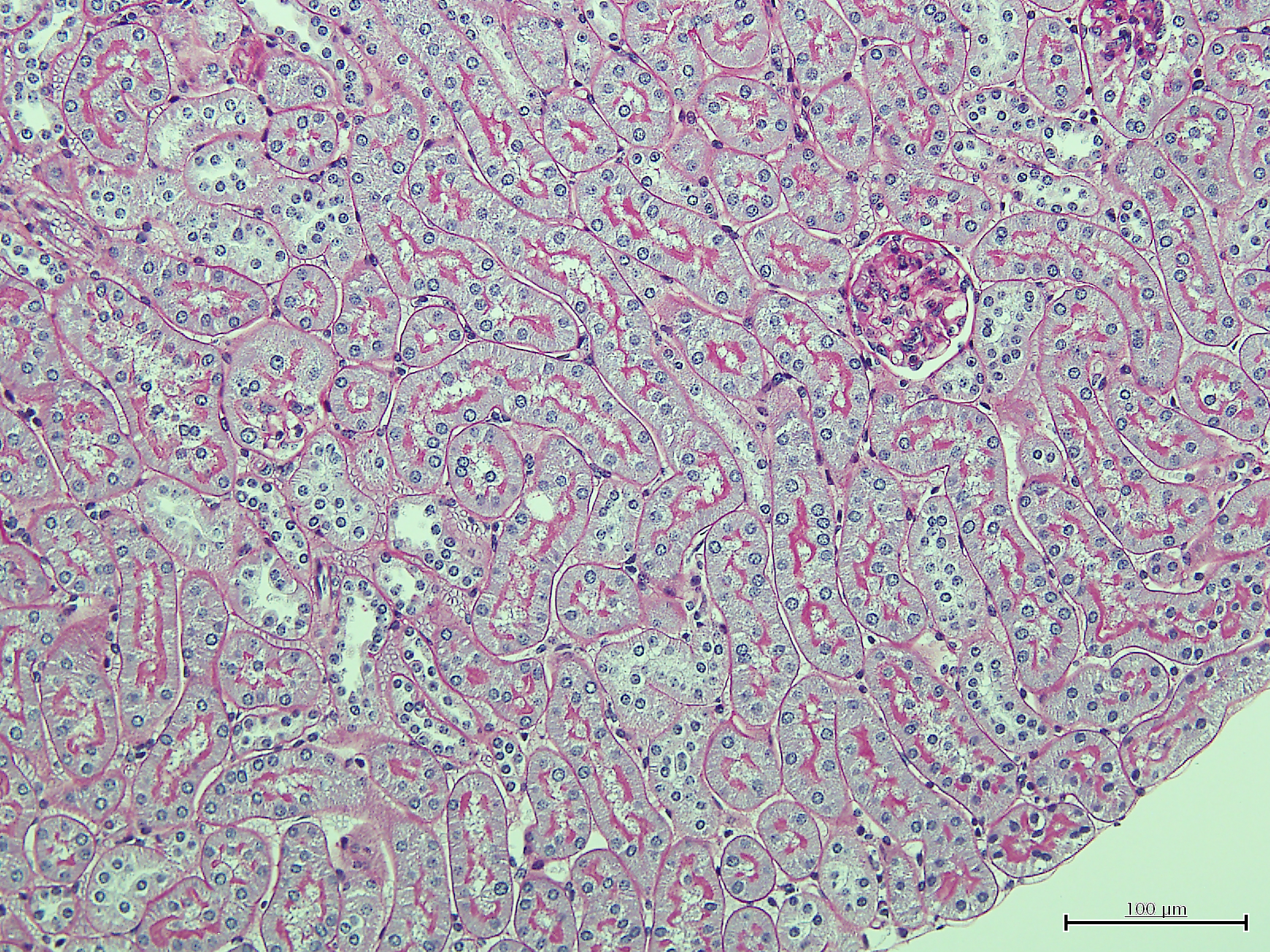

Supplement: Supplementary file 8 — EV and Appendix Figures Source Data [file 44318_2026_759_MOESM8_ESM.zip › SD EV Figureπü«πé│πâÆπéÜπâ╝/SD EV3/EV 3C/Flox/Flox_MediumMagnification.TIF]

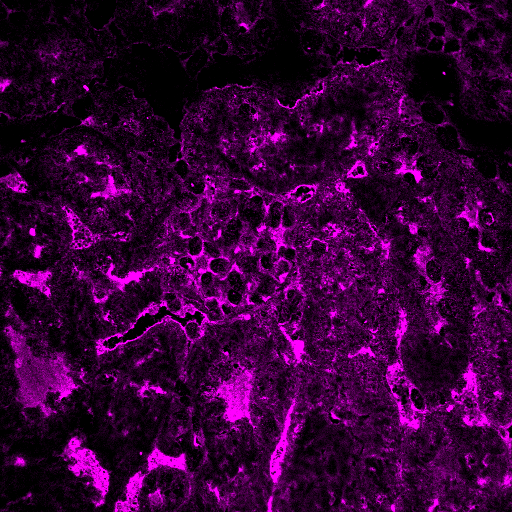

Supplement: Supplementary file 8 — EV and Appendix Figures Source Data [file 44318_2026_759_MOESM8_ESM.zip › SD EV Figureπü«πé│πâÆπéÜπâ╝/SD EV3/EV 3B/╬▒SMA_Cdkal1/Cdkal1.tif]

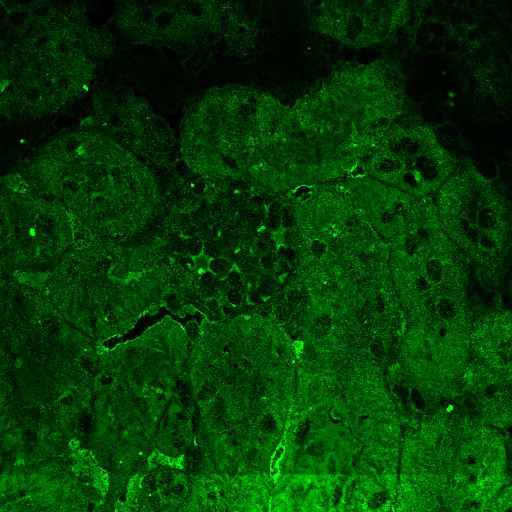

Supplement: Supplementary file 8 — EV and Appendix Figures Source Data [file 44318_2026_759_MOESM8_ESM.zip › SD EV Figureπü«πé│πâÆπéÜπâ╝/SD EV3/EV 3B/╬▒SMA_Cdkal1/╬▒SMA.tif]

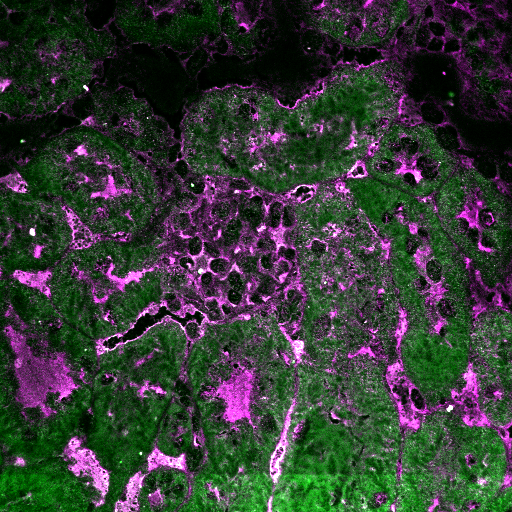

Supplement: Supplementary file 8 — EV and Appendix Figures Source Data [file 44318_2026_759_MOESM8_ESM.zip › SD EV Figureπü«πé│πâÆπéÜπâ╝/SD EV3/EV 3B/╬▒SMA_Cdkal1/merge.tif]

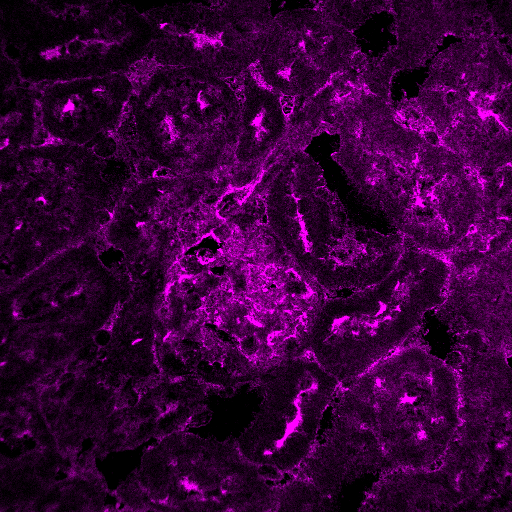

Supplement: Supplementary file 8 — EV and Appendix Figures Source Data [file 44318_2026_759_MOESM8_ESM.zip › SD EV Figureπü«πé│πâÆπéÜπâ╝/SD EV3/EV 3B/CD31_Cdkal1/Cdkal1.tif]

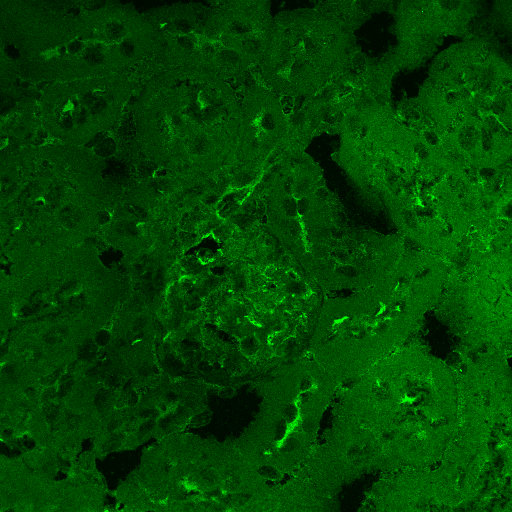

Supplement: Supplementary file 8 — EV and Appendix Figures Source Data [file 44318_2026_759_MOESM8_ESM.zip › SD EV Figureπü«πé│πâÆπéÜπâ╝/SD EV3/EV 3B/CD31_Cdkal1/CD31.tif]

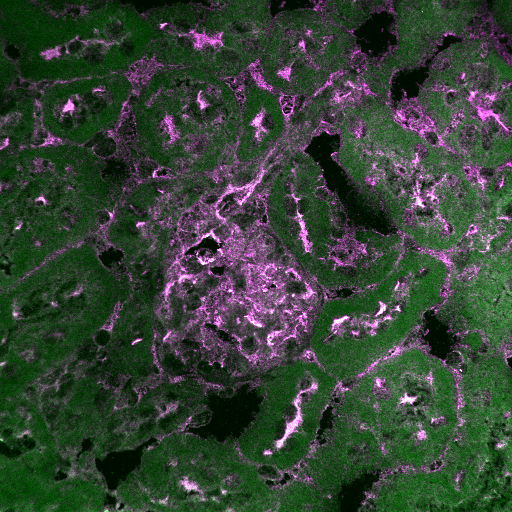

Supplement: Supplementary file 8 — EV and Appendix Figures Source Data [file 44318_2026_759_MOESM8_ESM.zip › SD EV Figureπü«πé│πâÆπéÜπâ╝/SD EV3/EV 3B/CD31_Cdkal1/merge.tif]

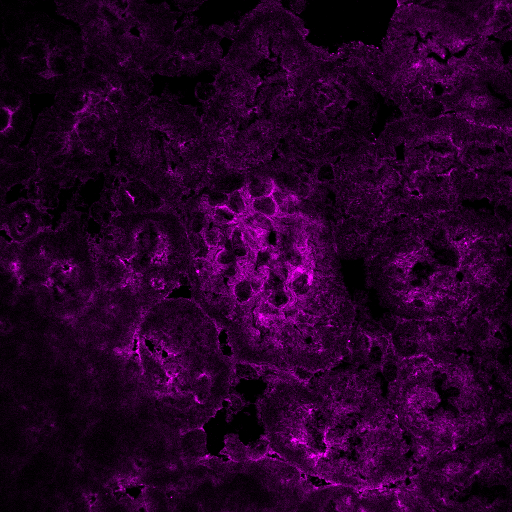

Supplement: Supplementary file 8 — EV and Appendix Figures Source Data [file 44318_2026_759_MOESM8_ESM.zip › SD EV Figureπü«πé│πâÆπéÜπâ╝/SD EV3/EV 3B/Podocin_Cdkal1/Cdkal1.tif]

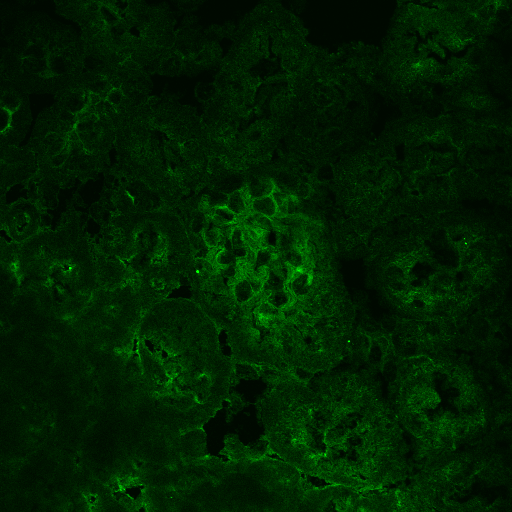

Supplement: Supplementary file 8 — EV and Appendix Figures Source Data [file 44318_2026_759_MOESM8_ESM.zip › SD EV Figureπü«πé│πâÆπéÜπâ╝/SD EV3/EV 3B/Podocin_Cdkal1/podocin.tif]

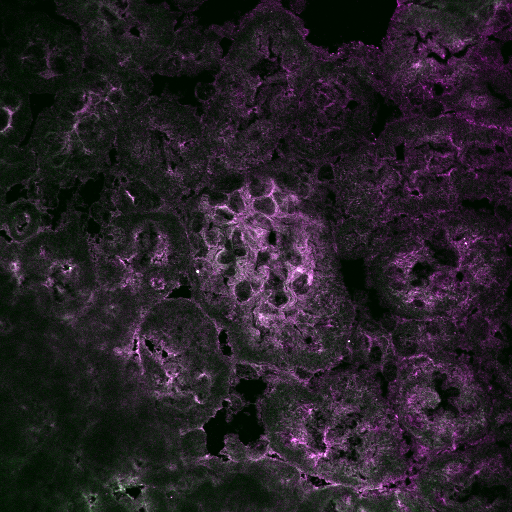

Supplement: Supplementary file 8 — EV and Appendix Figures Source Data [file 44318_2026_759_MOESM8_ESM.zip › SD EV Figureπü«πé│πâÆπéÜπâ╝/SD EV3/EV 3B/Podocin_Cdkal1/merge.tif]
