# Supplementary material for: CDKAL1 dysfunction impairs lysine codon translation in podocytes and accelerates chronic kidney disease
Source: EMBO J. 2026 Mar 28;45(9):3206–29. doi: 10.1038/s44318-026-00759-3 (PMC13144697; doi:10.1038/s44318-026-00759-3)
Supplement: Supplementary file 9 — Expanded View Figures [file 44318_2026_759_MOESM9_ESM.pdf]

Expanded View Figures

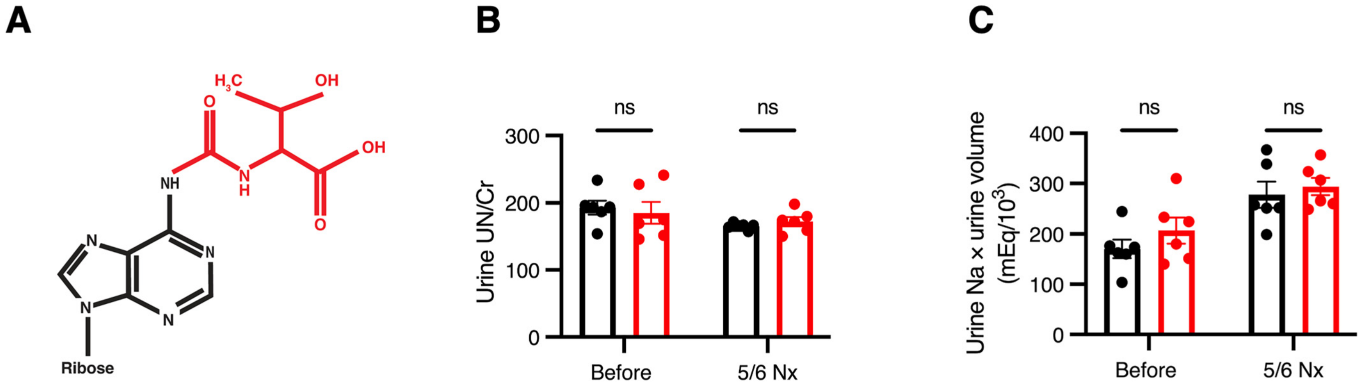

Figure EV1. Lack of tubular injury after 5/6 nephrectomy in systemic *Cdkal1* KO mice.

(**A**) Chemical structure of N^6-threonylcarbamoyladenosine (t^6A). Modified residue is shown in red. (**B**, **C**) Urinary urea nitrogen (UN) level normalized by urine creatinine (Cr) (**B**) and daily sodium excretion (**C**) in systemic *Cdkal1* KO and floxed mice before (8-week-old) and after 5/6 nephrectomy (12-week-old). $n = 6$ each. Data are presented as the mean ± SEM. n.s., not significant by two-way ANOVA followed by Sidak post-hoc test.

© The Author(s)

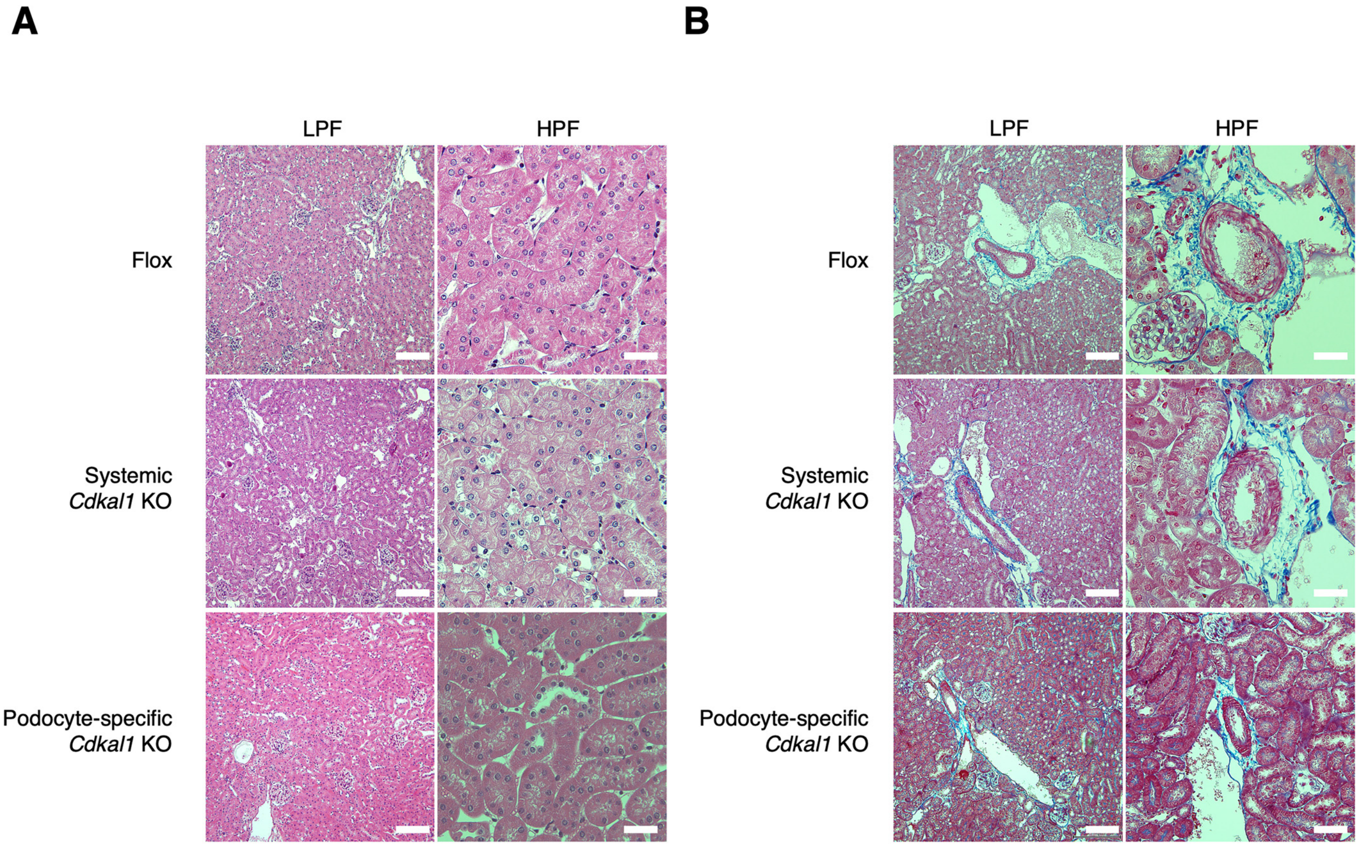

Figure EV2. Morphology of the renal tubules and vessels of the floxed mice, systemic *Cdkal1* KO mice, and podocyte-specific *Cdkal1* KO mice.

(**A**) HE stained renal tubules. (**B**) Azan–Mallory stained renal vessels of systemic *Cdkal1* KO mice, podocyte-specific *Cdkal1* KO, and floxed mice. Scale bars, 200 μm (low-power fields, LPF) and 50 μm (high-power fields, HPF).

© The Author(s)

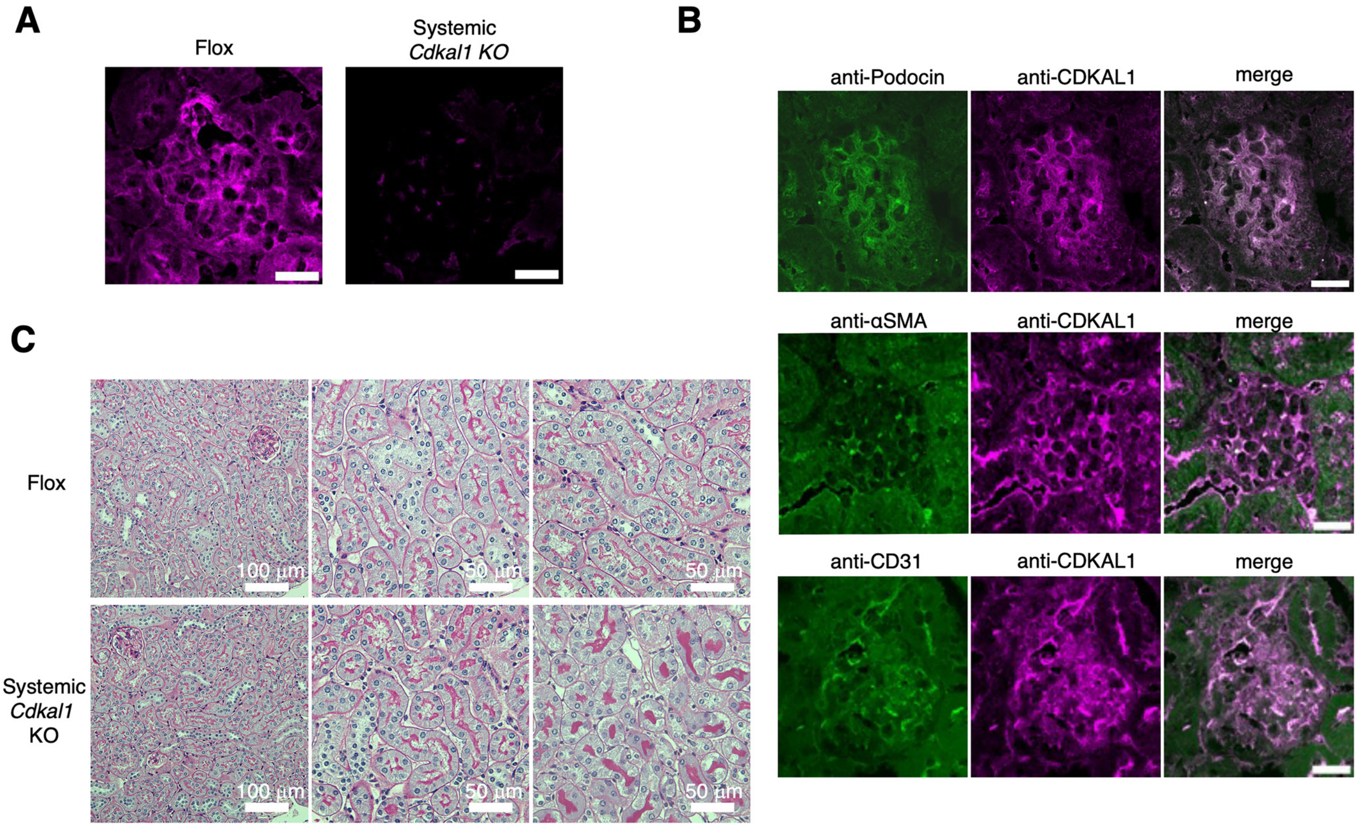

Figure EV3. Validation, localization and renal phenotype of CDKAL1 in mouse kidneys.

(**A**) Immunostaining with anti-CDKAL1 antibody shows no signal in glomeruli from systemic *Cdkal1* knockout (KO) mice, confirming the antibody's specificity. Scar bar, 20 µm. (**B**) Double immunofluorescence staining for podocin, αSMA or CD31 (green) with CDKAL1 (magenta) of the glomeruli of 20-week-old male mice. αSMA and CD31 are markers of mesangial cells and endothelial cells, respectively. Scale bars, 20 µm. (**C**) Representative PAS-stained renal tubules of 50-week-old systemic *Cdkal1* KO or floxed mice. Scale bars, 100 µm (left), 50 µm (center, right).

 © The Author(s)

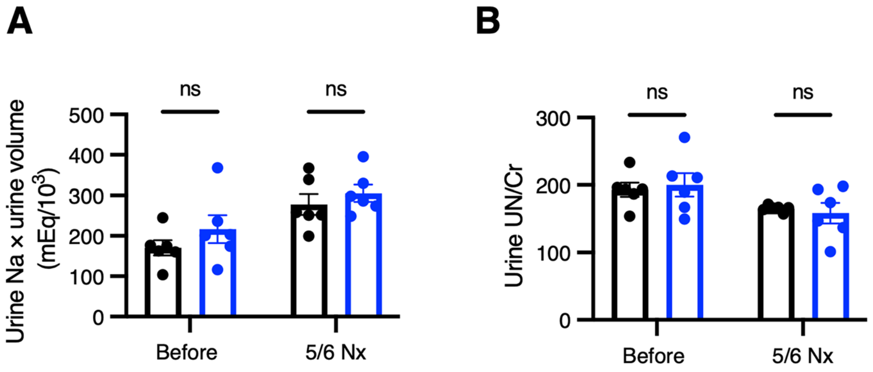

Figure EV4. No tubular injury in podocyte-specific *Cdkal1* knockout mice after 5/6 nephrectomy.

(**A**, **B**) Measurement of urinary urea nitrogen (UN) normalized by urine creatinine (Cr) (**A**) and daily sodium excretion (**B**) in podocyte-specific *Cdkal1* KO and floxed mice before (8-week-old) and after 5/6 nephrectomy (16-week-old). $n = 6$ each. Data are presented as mean ± SEM; n.s., not significant by two-way ANOVA followed by Sidak post-hoc test.

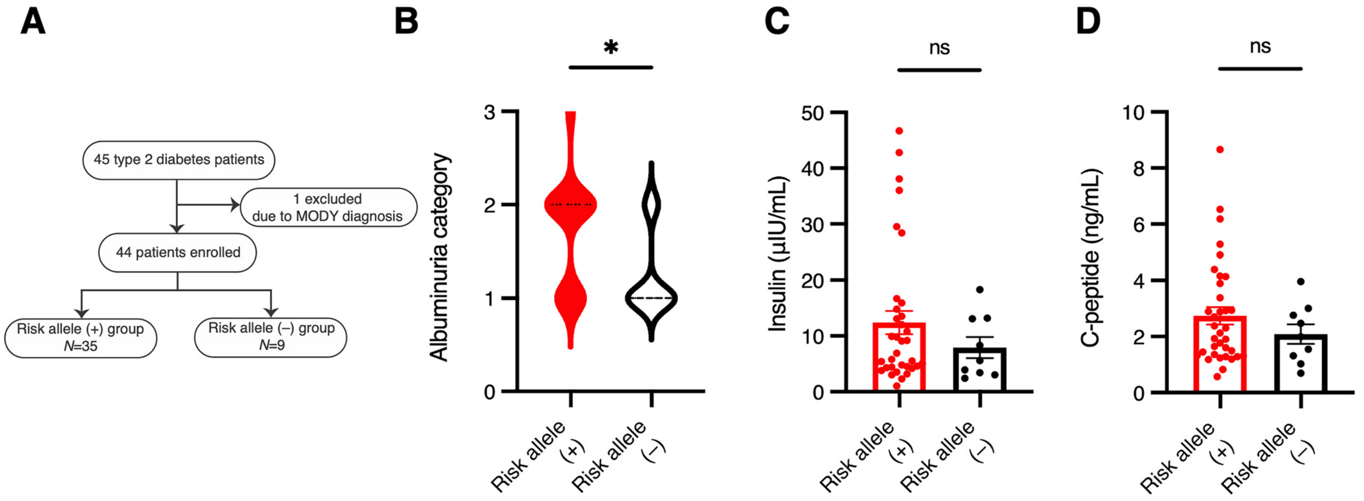

Figure EV5. Earlier onset of albuminuria in patients with type 2 DM and a *CDKAL1* SNP than in those without.

(**A**) Enrollment of patients in the clinical study. Out of 45 initial participants, one was excluded, resulting in 44 patients being analyzed. MODY, maturity onset diabetes of the young. (**B–D**) Albuminuria categories (**B**), plasma insulin levels (**C**), serum C-peptide levels (**D**) in patients with ($n = 35$) or without ($n = 9$) the risk allele. Albuminuria categories according to the KDIGO 2022 Clinical Practice Guideline (KDIGO, 2022). Data are presented as mean ± SEM. Statistical significance was determined by Mann–Whitney *U* test: *$P = 0.0264$ (**B**); n.s., not significant (**C**, **D**).

 © The Author(s)
